# The cycloaspeptides: uncovering a new model for methylated nonribosomal peptide biosynthesis†
†Electronic supplementary information (ESI) available. See DOI: 10.1039/c8sc00717a


**DOI:** 10.1039/c8sc00717a

**Published:** 2018-04-10

**Authors:** Kate M. J. de Mattos-Shipley, Claudio Greco, David M. Heard, Gemma Hough, Nicholas P. Mulholland, Jason L. Vincent, Jason Micklefield, Thomas J. Simpson, Christine L. Willis, Russell J. Cox, Andrew M. Bailey

**Affiliations:** a School of Chemistry , University of Bristol , Cantock's Close , Bristol , BS8 1TS , UK . Email: kd4495@bris.ac.uk; b Syngenta Ltd. , Jealott's Hill International Research Centre Bracknell , Berkshire , RG42 6EY , UK; c School of Chemistry , University of Manchester , Oxford Road , Manchester , M1 7DN , UK; d Institute für Organsche Chemie , Leibniz Universität Hannover , Schneiderberg 1A , 30167 Hannover , Germany; e BMWZ , Leibniz Universität Hannover , Schneiderberg 38 , 30167 Hannover , Germany; f School of Biological Sciences , University of Bristol , Life Sciences Building, 24 Tyndall Avenue , Bristol , BS8 1TQ , UK . Email: andy.bailey@bris.ac.uk

## Abstract

The cycloaspeptides are bioactive pentapeptides produced by various filamentous fungi, which have garnered interest from the agricultural industry due to the reported insecticidal activity of the minor metabolite, cycloaspeptide E. Genome sequencing, bioinformatics and heterologous expression confirmed that the cycloaspeptide gene cluster contains a minimal 5-module nonribosomal peptide synthetase (NRPS) and a new type of *trans*-acting *N*-methyltransferase (*N*-MeT). Deletion of the *N*-MeT encoding gene and subsequent feeding studies determined that two modules of the NRPS preferentially accept and incorporate *N*-methylated amino acids. This discovery allowed the development of a system with unprecedented control over substrate supply and thus output, both increasing yields of specific metabolites and allowing the production of novel fluorinated analogues. Furthermore, the biosynthetic pathway to ditryptophenaline, another fungal nonribosomal peptide, was shown to be similar, in that methylated phenylalanine is accepted by the ditryptophenaline NRPS. Again, this allowed the directed biosynthesis of a fluorinated analogue, through the feeding of a mutant strain. These discoveries represent a new paradigm for the production of *N*-methylated cyclic peptides *via* the selective incorporation of *N*-methylated free amino acids.

## Abstract 2

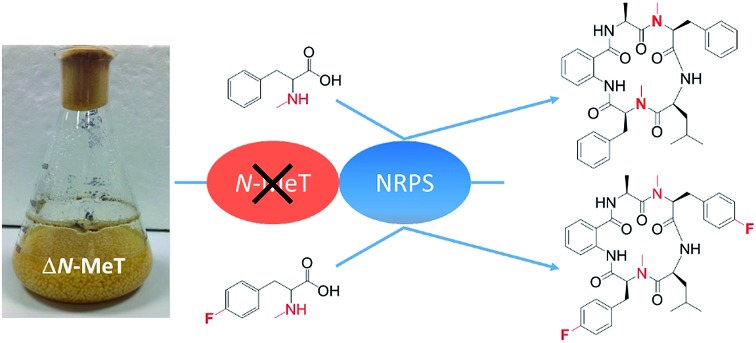
The cycloaspeptide gene cluster includes a pathway-specific *N*-methyl transferase. Its disruption allowed incorporation of *N*-methylated amino acids provided in the culture medium, allowing efficient production of cycloaspeptide E and novel related products.

## Introduction

**Chart 1:**
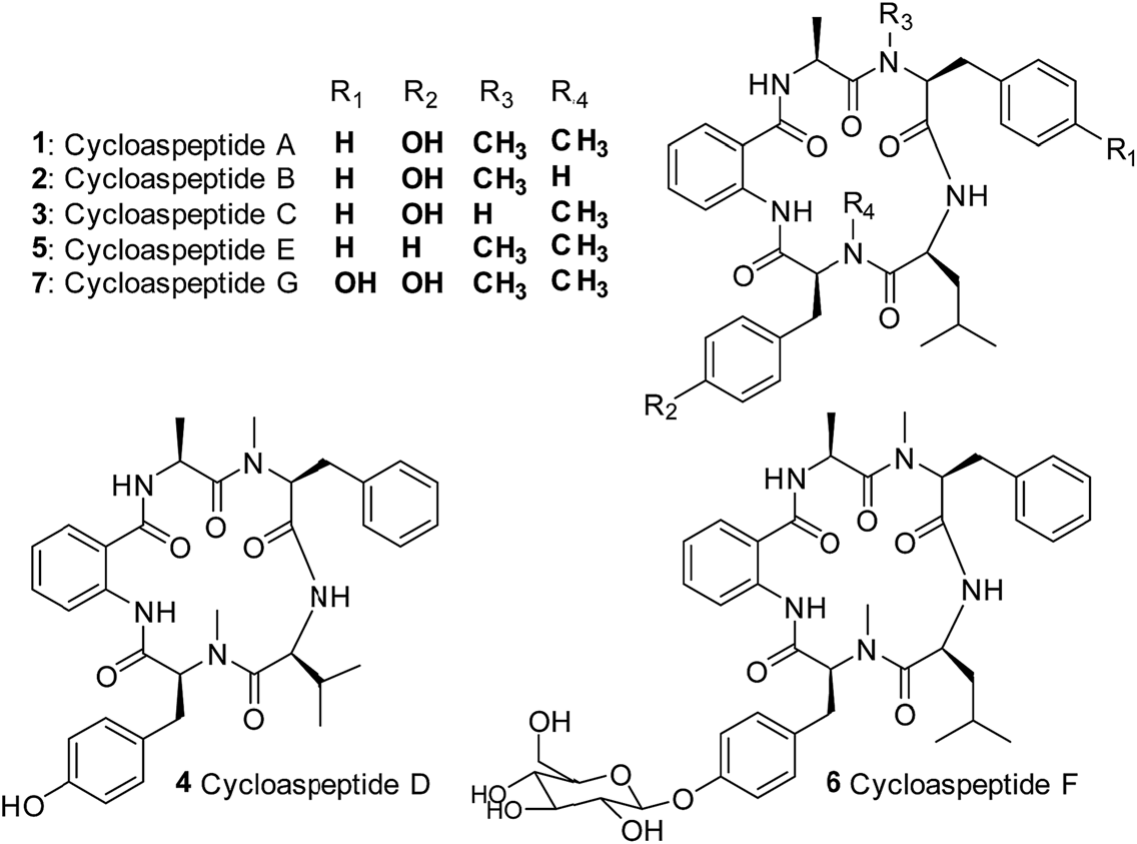
Structures of the cycloaspeptides – isolated from various filamentous fungi.

Cycloaspeptides are bioactive cyclic pentapeptides which were originally identified from an unclassified *Aspergillus* species in 1987.^1^ Cycloaspeptides A–C **1–3** contain anthranilic acid (position p1), l-alanine (p2), l-phenylalanine (p3), l-leucine (p4) and l-tyrosine (p5) residues, and differ only in the *N*-methylation pattern of the phenylalanine and tyrosine moieties (Chart 1). A later screen of the *Aspergillus* genus failed to identify any cycloaspeptide producers, but multiple cycloaspeptide-producing *Penicillium* species have since been identified.^2^–^4^ Cycloaspeptide D **4**, which contains a valine at p4, was isolated along with cycloaspeptide A **1** from *Penicillium ribeum* and *P. algidum*, in work which also identified these cycloaspeptides as being active against the malarial parasite *Plasmodium falciparum*.^3^,^4^

In 2006, cycloaspeptide E **5** was isolated as a minor metabolite from several *Penicillium* species and one *Trichothecium* species, and reported to exhibit insecticidal activity against lepidoptera.^5^**5** differs from **1** in having phenylalanine instead of tyrosine at p5 (Chart 1), and was proposed to have a neurotoxic mode of action. There is significant interest in natural products with insecticidal properties due to the huge economic and social burden created by insect damage to crop species worldwide.^6^

Further research into the bioactivity of cycloaspeptide E **5** has been hampered by the fact that yields are only approximately 2% of the main metabolite; cycloaspeptide A **1**, meaning that purification for bioactivity screening is problematic and the natural titres are too low to be commercially viable. Most recently cycloaspeptides F **6** and G **7** from the entomopathogenic fungus *Isaria farinosa*, have been isolated and characterized. These compounds have cytotoxic properties against tumour cell lines.^7^

In order to investigate cycloaspeptide biosynthesis, with the key aim of improving the yields of **5**, we set out to discover and manipulate the cycloaspeptide biosynthetic pathway, using two publicly available cycloaspeptide producers, *Penicillium soppii* (CBS 869.70) and *Penicillium jamesonlandense* (CBS 102888).^2^ Our original working hypothesis was that the cycloaspeptides would fit the accepted paradigm for methylated cyclic peptide biosynthesis, and as such would consist of a traditional 5-module NRPS containing the two requisite *N*-methyltransferase (*N*-MeT) domains. We reasoned that substrate promiscuity of the NRPS could account for the range of cycloaspeptides reported, whereas we considered a RiPP system unlikely due to the fidelity of ribosomally synthesised peptides to their encoded precursor peptide sequence. A combination of approaches including bioinformatic analyses, heterologous expression, gene deletions and feeding experiments have demonstrated that indeed, an NRPS is responsible for the biosynthesis of the cycloaspeptides. However, rather than containing *N*-Met domains, the NRPS requires a pathway-specific *trans*-acting methyltransferase to provide the necessary substrates and preferentially incorporates methylated amino acids at two positions. This discovery unlocked a new avenue for pathway engineering through synthetic biology, by allowing unprecedented control over substrate availability.

## Results and discussion

*P. soppii* and *P. jamesonlandense* were cultured on a range of solid media and screened by LCMS for the production of cycloaspeptides. A compound with the correct mass for cycloaspeptide A **1** could be reliably detected by LCMS, from both species and on all media after 2 weeks. This was confirmed by purification and NMR analysis (Table S8†). *P. soppii* was also grown in three different liquid media (CYB, MEB and YEB), and again **1** could be detected in all, with the highest concentrations being achieved with CYB (Fig. S21†). A compound with the correct mass for cycloaspeptide E **5** could be detected in certain cultures, but only in trace amounts and not reliably. Analysis using the highly sensitive UPLC-Orbitrap mass analyser system confirmed that the accurate masses matched the chemical formulae of the predicted compounds (Fig. S22 and Table S6†). The sensitivity of this system also allowed the detection of a compound with the correct predicted formula to be cycloaspeptide G **7**, a compound which has not been detected in a *Penicillium* species before.

Cycloaspeptide A **1** could also be easily detected by LCMS in MEB cultures of *P. jamesonlandense* (Fig. S23, S26 and Table S7†). There were also compounds present with the correct masses to be cycloaspeptides D **4**, E **5** and G **7** (Table S7†). Unfortunately, the yields of **5** were too low to purify for NMR and structural validation.

Paired-end genomic sequence data was generated for both species and assembled to produce draft genomes (Table S2†), which were analysed using AntiSMASH^8^ to identify putative biosynthetic gene clusters (BGCs). A total of 82 and 83 BGCs were detected for *P. soppii* and *P. jamesonlandense* respectively, demonstrating the rich metabolic potential of these species (Table S3†).

*P. soppii* contains 20 NRPS clusters whereas *P. jamesonlandense* contains 17, with 8 such clusters being common to both species. The domain architecture of each predicted NRPS was analysed using the NCBI conserved domain database (CDD) (Table S4†). Only one was found to contain the five modules that would be expected for pentapeptides such as the cycloaspeptides (Fig. S7†). Unexpectedly this NRPS lacked any integral *N*-methyltransferase (*N*-MeT) domains, which appeared inconsistent with cycloaspeptide biosynthesis, as *N*-methylation, if present, is normally performed by such domains embedded within the NRPS. However, an adjacent gene present in both species is predicted to encode a methyltransferase, so might perform this activity *in trans*. Additionally, the NRPS appears to contain a final condensation-like (CT) domain, where rather than having the highly conserved HHXXXDXXS/T motif found in condensation domains, it has the modified motif SHXXXDXXS/T (5377SHAQYDGVS5385). CT domains are known to catalyse the macrocyclisation of cyclic peptides in filamentous fungi^9^ – a role analogous to that of the final thiolesterase in bacterial cyclic peptide biosynthesis.

**Fig. 1:**
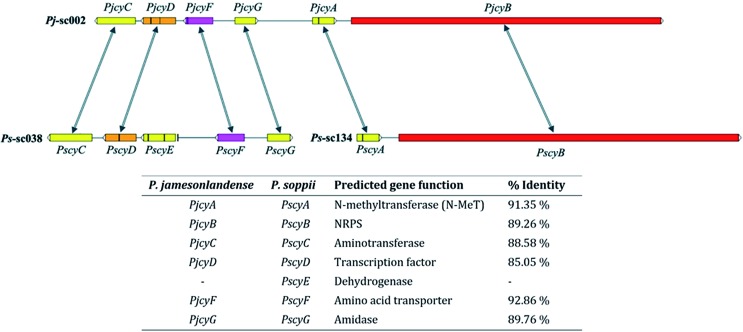
Cycloaspeptide gene clusters from *P. jamesonlandense* (top) and *P. soppii* (bottom). The *N*-methyltransferase (*PjcyA* and *PscyA*) and NRPS (*PjcyB* and *PscyB*) genes are adjacent to one another in both species. Additional genes identified at the same locus in *P. jamesonlandense*, which could potentially be involved in secondary metabolite biosynthesis, such as an amino acid transporter and transcription factor were identified on a separate scaffold in *P. soppii*. Arrows denote homologous genes; vertical black bars within genes denote introns.

In *P. jamesonlandense*, adjacent to the NRPS and putative *N*-MeT encoding genes are various additional predicted genes that could potentially be involved in cycloaspeptide production. These encode an amino acid transporter, a Zn_2_Cys_6_ transcription factor and an aminotransferase. In *P. soppii* however, these genes are translocated on another scaffold (sc038, Fig. 1). It is still possible that they are involved in cycloaspeptide biosynthesis, as split secondary metabolite clusters have been observed in fungi multiple times.^10^ However, it is also possible that the NRPS and *N*-MeT are the only proteins required for the production of cycloaspeptides.

Bioinformatic analysis was conducted for the adenylation (A) domains of the NRPS. This focused on the predicted presence of an anthranilic acid adenylating domain, due to their distinct nature.^11^ The online NRPS predictor tool^12^ was used to generate the 10 amino acid code for each domain (Table 1), and these were compared with those of known fungal NRPS enzymes.^11^ The presence of a glycine in the first position of the 10 amino acid code for domain 1, rather than the aspartic acid typically seen, is indicative of domain 1 being an anthranilic acid adenylating domain. This is consistent with the fact that all known fungal anthranilic acid A-domains are positioned within the first module of the synthetase.^11^ It then follows that the second module incorporates an alanine, the third a phenylalanine, the fourth a leucine, and the fifth, either a tyrosine (to produce **1**) or a phenylalanine (to produce **5**). Evidence supporting this assignment is the similarity of the third and fifth modules, which are predicted to incorporate Phe and Phe/Tyr respectively. In *P. soppii* these domains have 80% sequence similarity when comparing the 10 amino acid code, and 85% sequence similarity when comparing the 34 extracted residues. Interestingly, it was not possible to identify homologous gene clusters in publicly available fungal genomes, even when specifically searching *Aspergillus*, *Penicillium*, *Trichothecium* or *Isaria* genomes for homologues of *PscyA* and *PscyB*. This suggests that although the cycloaspeptides have been reported from various fungal genera, the gene cluster is not particularly wide-spread and cycloaspeptide biosynthesis may be limited to a fairly small fungal clade, or clades.

**Table 1:** The 10 amino acid codes for the *P. soppii* cycloaspeptide NRPS (*PscyB*) A domains, analysed using the online tool NRPSpredictor2 12

Module	Pos1 (235)	Pos2 (236)	Pos3 (239)	Pos4 (278)	Pos5 (299)	Pos6 (301)	Pos7 (322)	Pos8 (330)	Pos9 (331)	Pos10 (517)	Predicted substrate
1	G	V	I	F	I	A	A	G	I	K	Ant
2	D	V	F	F	V	V	G	V	L	K	Ala
3	D	A	Y	A	V	G	G	I	C	K	Phe
4	D	L	M	L	V	G	A	V	I	K	Leu
5	D	A	Y	T	S	G	G	I	C	K	Tyr/Phe

**Fig. 2:**
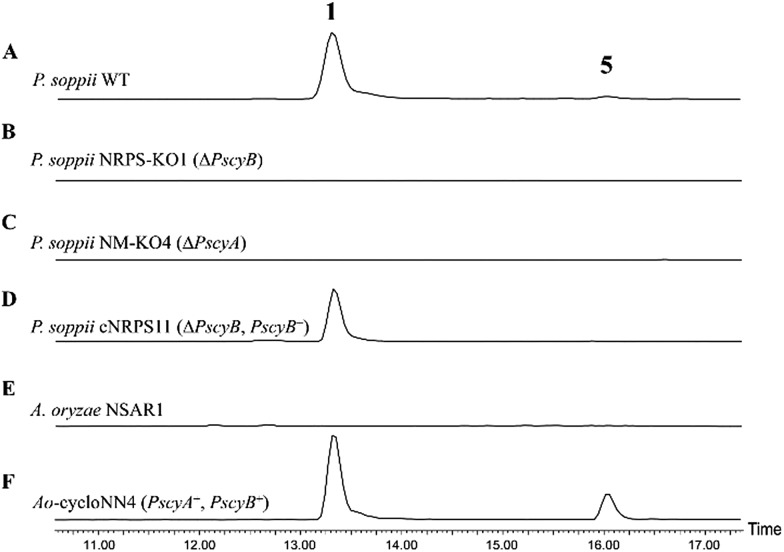
ES+ extracted ion chromatograms for **1** ([M + H]^+^: 642.7) and **5** ([M + H]^+^: 627.7) in various fungal cultures: (A) wild-type *P. soppii* produces both **1** and **5**; (B) *P. soppii* Δ*PscyB* strain, NRPS-KO1, produces no cycloaspeptides; (C) *P. soppii* Δ*PscyA* strain, NM-KO1, produces no cycloaspeptides; (D) NRPS-KO1 complemented with a wild-type copy of the cycloaspeptide NRPS (Δ*PscyB*, *PscyB*+), demonstrates a restoration in cycloaspeptide biosynthesis; (E) wild-type *A. oryzae* NSAR1; (F) strain Ao-cycloasNN4, which is an *A. oryzae* NSAR1 transformant co-expressing *PscyA* and *PscyB*, produces both cycloaspeptide A and cycloaspeptide E.

To allow an investigation of gene function in *P. soppii*, a protoplast-based transformation system was developed, using either hygromycin or geneticin resistance as selectable markers. This transformation system was optimized using eGFP as a reporter gene (Fig. S9†) and a bipartite knock-out strategy^13^ was then used to disrupt specific genes. The disruption of either the NRPS (*PscyB*) or the *N*-MeT (*PscyA*) led to a complete loss of production of both cycloaspeptide A **1** and E **5**, (Fig. 2A–C) confirming that both genes are required for cycloaspeptide biosynthesis and that **1** and **5** are synthesized by the same pathway.

The disruption of the transcription factor (*PscyD*) led to a marked reduction in cycloaspeptide yields, suggesting that although it is translocated in *P. soppii*, it is still involved in the regulation of cycloaspeptide biosynthesis (Fig. S24†).

To further confirm the identity of the gene cluster and conclusively demonstrate that the loss of production was not an indirect consequence of the transformation system, strain NRPS-KO1 (Δ*PscyB*) was complemented by transformation with the plasmid pTYGen–*N*-MeT–NRPS which contains *PscyA* and *PscyB* under the control of P*adh* (the alcohol dehydrogenase promoter from *A. oryzae*) and P*tub* (the tubulin promoter from *P. soppii*) respectively. Selection of geneticin resistant transformants led to the restoration of cycloaspeptide production (Fig. 2D).

Heterologous production of both **1** and **5** was achieved in *Aspergillus oryzae* NSAR1 *via* the co-expression of *PscyA* and *PscyB* from a multi-gene expression vector with arginine prototrophy selection (Fig. 2E and F).^14^ This demonstrates that *PscyA* and *PscyB* are the only two structural genes required for cycloaspeptide biosynthesis. Interestingly, the ratio of cycloaspeptide A **1** to E **5** appeared to differ in *A. oryzae* when compared to the natural producers, with cycloaspeptide E **5** being easily detectable in initial screens of the *A. oryzae* transformants. An apparent decrease in fitness and growth rate was observed in *A. oryzae* transformants producing cycloaspeptides, and production was not stable. A loss of production occurring after subculturing prevented quantification of titres. In an attempt to push the ratio of 5 : 1 further in favour of cycloaspeptide E **5** biosynthesis, a full A domain swap was conducted. The seemingly promiscuous A domain from module 5 (Tyr/Phe) was replaced with a second copy of the A domain from module 3 (Phe). This engineered NRPS was expressed both in *A. oryzae* and the *P. soppii* Δ*PscyB* strain but the NRPS was dysfunctional, with no cycloaspeptides being detected in either strain (data not shown).

**Fig. 3:**
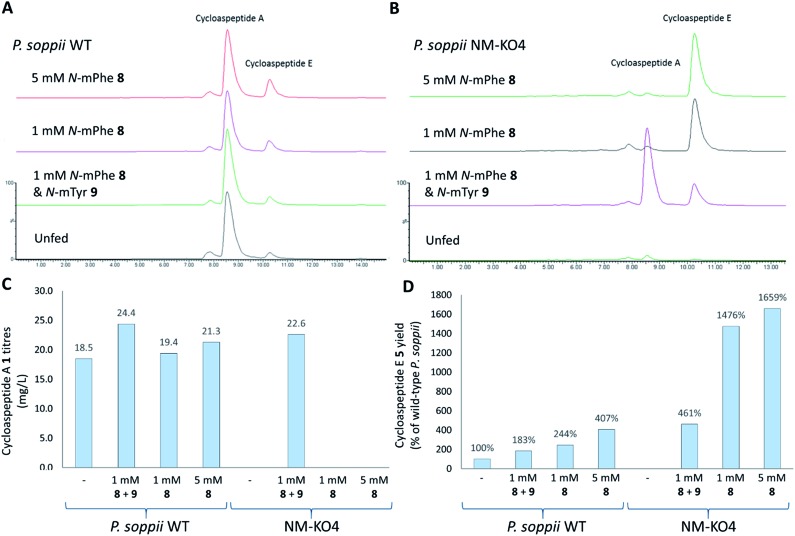
Feeding *P. soppii* cultures with *N*-methylated phenylalanine **8** and *N*-methylated tyrosine **9** was shown to impact cycloaspeptide biosynthesis, increasing titres in wild-type *P. soppii* and restoring cycloaspeptide production in an *N*-MeT knock-out strain (NMKO4: Δ*PscyA*). (A) ES+ extracted ion chromatograms for cycloaspeptide A **1** ([M + H]^+^: 642.7) and cycloaspeptide E **5** ([M + H]^+^: 627.7). Wild-type cultures, from bottom to top: unfed control. Fed with 1 mM each of **8** and **9**. Fed with 1 mM **8**. Fed with 5 mM **8**. (B) NMKO4 (Δ*PscyA*) cultures, from bottom to top: unfed control. Fed with 1 mM each of **8** and **5**. Fed with 1 mM **8**. Fed with 5 mM **8**. (C) Absolute titres of **1** (mg L^–1^) in various fed cultures. Some differences were observed for the fed wild-type cultures and complete restoration was seen for the *N*-MeT knock-out strain. (D) Relative titres of **5** (% of wild-type) in the various cultures demonstrating an increase in **5** with feeding, particularly in the knock-out strain NM-KO4. Quantification was not possible for **5** due to a lack of cycloaspeptide E standard, so relative titres were calculated.

To ascertain the order of events in the cycloaspeptide biosynthetic pathway, LCMS chromatograms for the *N*-MeT knock-out strains were searched for any putative un-methylated intermediates. The absence of any such compounds suggested that rather than the *N*-methylations serving to decorate the product of the NRPS, the *N*-MeT may act first, providing methylated amino acids for incorporation by the NRPS. To test this, *N*-MeT knock-out strain 4 (NM-KO4) was fed with 1 mM of both *N*-methylated tyrosine (*N*-mTyr **8**) and *N*-methylated phenylalanine (*N*-mPhe **9**). This fully restored cycloaspeptide biosynthesis (Fig. 3B), demonstrating that the NRPS accepts free methylated amino acids, a feature not previously observed in non-ribosomal peptide systems in either fungi or bacteria.

In further experiments, wild-type *P. soppii* and NM-KO4 cultures were fed with either a 1 : 1 mixture of *N*-mPhe **8** and *N*-mTyr **9** (1 mM each), or with *N*-mPhe alone (either 1 mM or 5 mM final culture concentration) (Fig. 3). Increases in the cycloaspeptide yields when the WT strain was fed with the *N*-methylated amino acids suggests that substrate availability is a limiting factor in this system (Fig. 3C). The yields of the cycloaspeptides in the fed NM-KOS cultures were most striking. In addition to producing wild-type yields of cycloaspeptide A **1**, cultures fed with both *N*-mPhe **8** and *N*-mTyr **9** produced over four times more cycloaspeptide E **5** than the unfed wild-type strain (Fig. 3D). When supplemented with either 1 mM or 5 mM *N*-mPhe **8** cycloaspeptide E **5** titres were increased further, to approximately 14.5 and 16.5 times respectively (Fig. 3D).

**Fig. 4:**
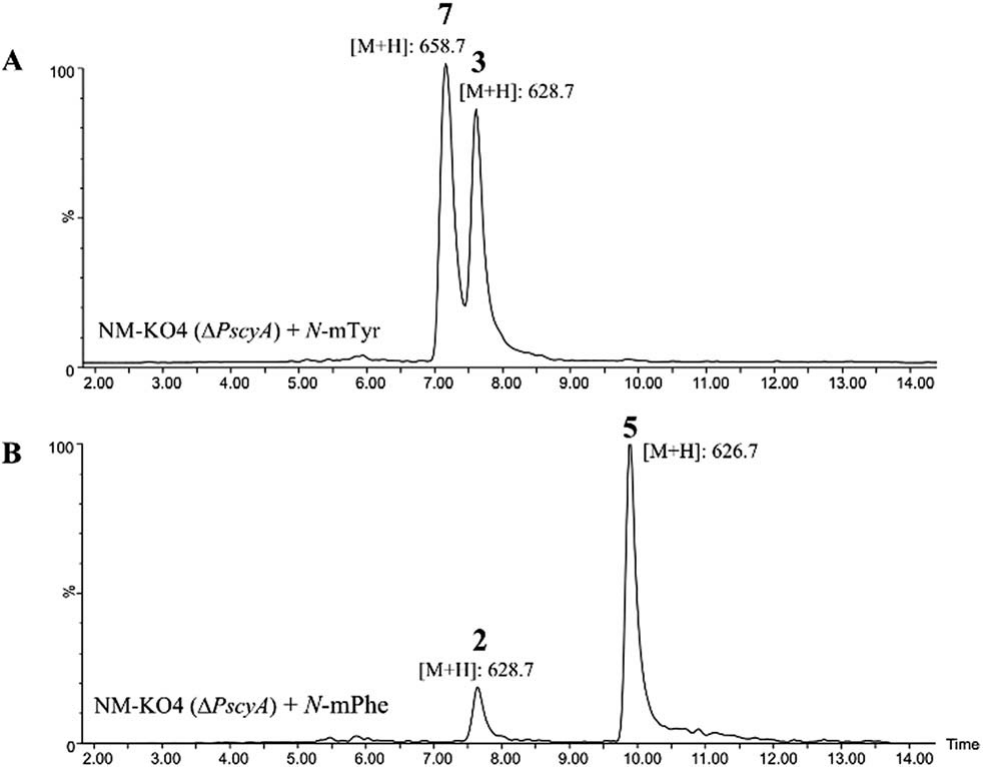
(A) ES+ extracted ion chromatogram for masses of 628.7, which corresponds to cycloaspeptide C **3** ([M + H]^+^) and 658.7 which corresponds to cycloaspeptide G **7** ([M + H]^+^); (B) ES+ extracted ion chromatogram for masses of 628.7 which corresponds to cycloaspeptide B **2** ([M + H]^+^) and 626.7 which corresponds to compound cycloaspeptide E **5** ([M + H]^+^). These compounds are not present in NM-KO4 (Δ*PscyA*), unless cultures are fed with methylated amino acids.

Such increased yields allowed purification of cycloaspeptide E **5** for structural confirmation by NMR (Table S9 and Fig. S23 and S24†). In addition to **5**, a compound could be detected in NM-KO4 cultures fed with *N*-mPhe **8** that has the expected mass and UV to be cycloaspeptide B **2** (Fig. 4). **2** was identified from the original *Aspergillus* sp. NE-45,^1^ and has a methylated phenylalanine at p3, but an unmethylated tyrosine at p5. This compound has not been reported in *Penicillium* species before.

A minor metabolite with the correct accurate mass to be cycloaspeptide G **7**, which has methylated tyrosine at both p3 and p5, was detected in wild-type *P. soppii* cultures using an UPLC-Orbitrap mass analyser. To determine whether the titres of this compound could be increased, NM-KO4 cultures were fed with *N*-mTyr alone.

This resulted in the easy detection of **7** in all fed cultures using standard LC-MS methods (Fig. 4). Feeding with *N*-mTyr also led to the production of a compound not detected in the wild-type cultures which has the expected mass to be cycloaspeptide C **3**. **3** has un-methylated phenylalanine at p3 and *N*-mTyr at p5. As with **5**, this compound has not been previously observed in *Penicillium* strains. These results indicate that the third and fifth modules of the NRPS have a strong preference for methylated amino acids, but also the ability to accept and incorporate un-methylated amino acids.

**Chart 2:**
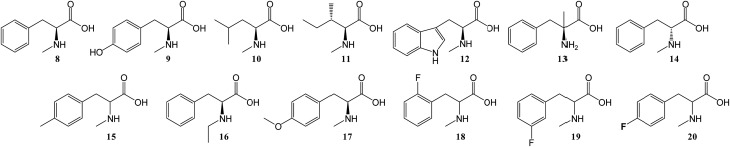
Various amino acid analogues fed to *P. soppii* NM-KO4 (Δ*PscyA*).

**Scheme 1:**
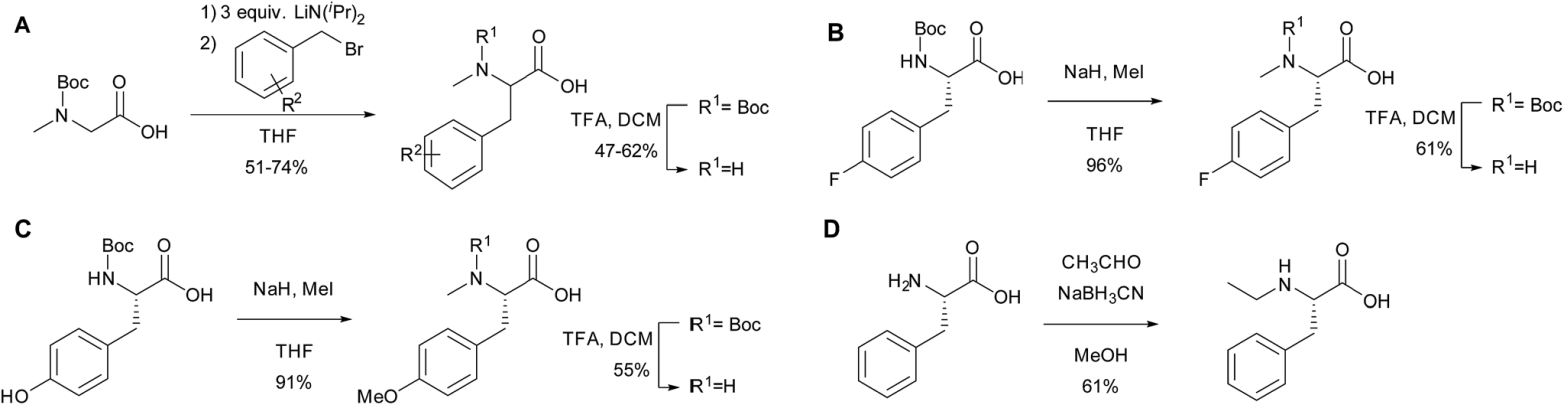
The synthesis of amino acid analogues.

To further investigate the substrate selectivity of the NRPS, a range of alternative amino acids were fed to strain NM-KO4. *N*-Methyl-leucine **10**, *N*-methyl-isoleucine **11**, *N*-methyl-tryptophan **12**, α-methyl-phenylalanine **13** and *N*-methyl-d-phenylalanine **14** were obtained commercially (Chart 2). Racemic *p*-methyl-*N*-mPhe **15**, and a range of fluorinated *N*-mPhe analogues (with the fluorine at the *ortho*, *meta* and *para* positions **18–20**) were synthesised by alkylation of the Boc–*N*-methylglycine dianion with the appropriate benzyl bromides, followed by Boc deprotection with trifluoroacetic acid (TFA) (Scheme 1A). *N*,*O*-Dimethyl-l-Tyr **17** was synthesised by double deprotonation of Boc-l-tyrosine with sodium hydride, followed by dimethylation with methyl iodide (Scheme 1C). Boc deprotection was again performed with TFA. *N*-Ethyl-l-Phe **16** was synthesised by reductive amination of l-phenylalanine with acetaldehyde and sodium cyanoborohydride (Scheme 1D).

**Fig. 5:**
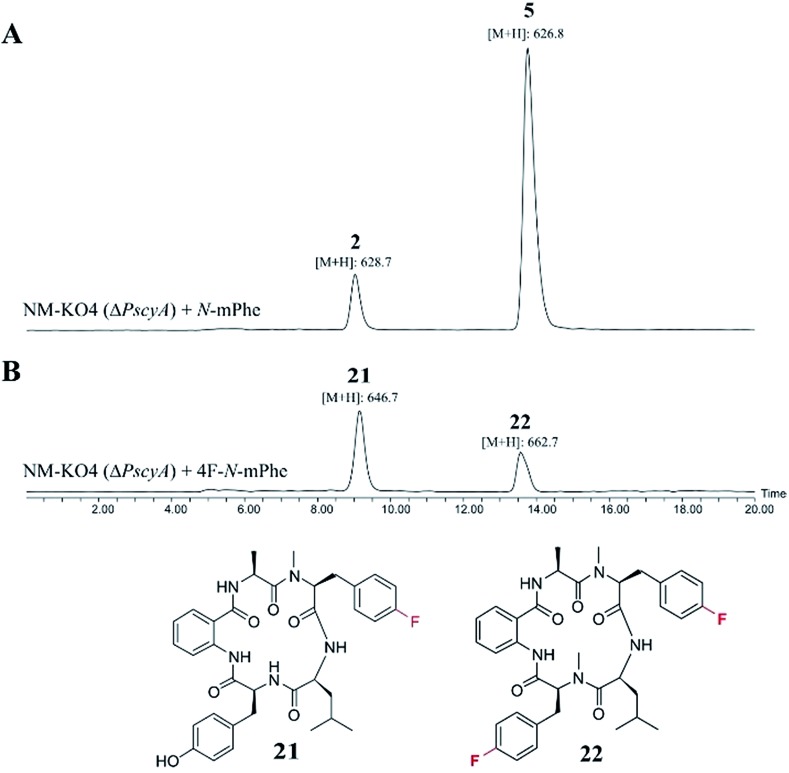
ES+ extracted ion chromatograms ([M + H]^+^) for cycloaspeptides B **2** (*m*/*z*: 628.7) and E **5** (*m*/*z*: 626.7), and their fluorinated counterparts **21** (*m*/*z*: 646.7) and **22** (*m*/*z*: 662.7). (A) NM-KO4 (Δ*PscyA*) cultures fed with *N*-mPhe **8**, produced **2** and **5**. (B) Cultures fed with 4F–*N*-Met–Phe **20**, produced **21** and **22**.

These compounds were either fed alone (5 mM) or in combination with *N*-mPhe **8** (at a ratio of 5 : 1–5 mM : 1 mM). The only cultures to produce novel compounds in the initial screens were those supplemented with fluorinated phenylalanine analogues. The synthesis of 4F–*N*-mPhe **20** was therefore scaled up (see ESI†) and an LCMS analysis identified compounds with the correct masses to be fluorinated analogues of cycloaspeptide E **5**, and cycloaspeptide B **2** (Fig. 5). 4F–cycloaspeptide E **22** was purified and characterized using proton, carbon and fluorine NMR (Table S10 and Fig. S28–S30†). Purified **5** and **22** were screened for anti-lepidopteran activity using the tobacco budworm (*H. virescens*) as an industrially relavent target organism, but no activity was observed against this species following injection with either compound (Table S12†).

A search of the NCBI database for any potential *PscyA* homologues identified an *N*-MeT from *A. flavus* with 39% sequence identity to *PscyA* at the protein level (Table S5†). Investigating the genomic context of this gene determined that it is part of a BGC containing a 2 module NRPS and cytochrome P450, which has been identified as the ditryptophenaline **23** gene cluster by Watanabe and coworkers.^15^**23** is a dimeric diketopiperazine consisting of two cyclic Trp:*N*-Met-Phe dipeptides.

**Fig. 6:**
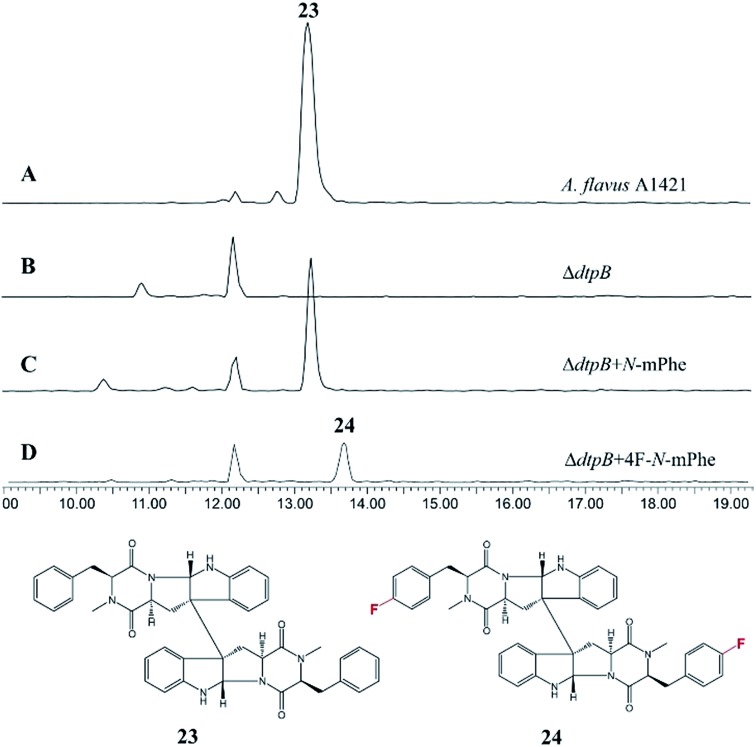
HPLC ES+ spectrum for the crude extracts of various *A. flavus* cultures: (A) *A. flavus* strain A1421 produces ditryptophenaline **23**; (B) a Δ*dtpB* strain can no longer produce **23**; (C) feeding the Δ*dtpB* strain with *N*-methylated phenylalanine restores the production of ditryptophenaline **23**. (D) Feeding the Δ*dtpB* strain with a fluorinated phenylalanine analogue (4F–*N*-mPhe **20**) led to the production of a fluorinated ditryptophenaline analogue **24**.

An *A. flavus* strain with the ditryptophenaline *N*-MeT disrupted (Δ*dtpB*) was kindly supplied by Prof. Kenji Watanabe of the University of Shizuoka, and feeding studies were conducted. Analogous to the cycloaspeptide system, feeding cultures with *N*-mPhe **8** fully restored ditryptophenaline **23** production, demonstrating the acceptance of **8** by the ditryptophenaline NPRS (*dtpA*). Furthermore, the production of a fluorinated ditryptophenylene analogue **24** was again achieved by feeding cultures with 4F–*N*-mPhe **20** (Fig. 6). Interestingly, feeding with *N*-mTyr **9** did not result in the production of hydroxylated analogues, demonstrating that the ditryptophenaline NRPS exhibits lower promiscuity than the cycloaspeptide NRPS. The rapid discovery of a second NRPS which accepts methylated amino acids suggests that these systems represent a new fungal route to methylated peptide natural products, rather than the cycloaspeptide system being unique.

## Conclusions

The gene cluster responsible for the biosynthesis of the cycloaspeptides has been identified in two psychrotolerant *Penicillium* species: *P. soppii* and *P. jamesonlandense*. Heterologous expression in *A. oryzae* has demonstrated that the minimal gene set required to produce both cycloaspeptide A **1** and cycloaspeptide E **2** is a 5-module NRPS and a new type of pathway specific *N*-methyltransferase (*N*-MeT). Gene knock-outs and feeding studies have demonstrated that two modules of the NRPS preferentially accept and incorporate *N*-methylated amino acids, which are provided by the pathway specific *N*-MeT, a system not previously seen in secondary metabolism. Selective amino acid *N*-methyltransferases are very rare in either primary or secondary metabolism. The only known enzyme to catalyse a single *N*-methylation of a proteogenic amino acid is glycine *N*-methyltransferase from primary metabolism. The most similar known examples from fungal secondary metabolism are 4-dimethylallyltryptophan *N*-methyltransferase, which is the second pathway-specific enzyme in the production of ergot alkaloids,^16^ and a methyltransferase from the ergothioneine pathway which trimethylates histidine.^17^

Deletion of the cycloaspeptide *N*-Met allowed the supply of methylated amino acids to the NRPS to be controlled, and this was exploited to direct biosynthesis towards specific minor metabolites, accomplishing the original aim of increasing cycloaspeptide E **5** yields. Feeding cultures with a fluorinated phenylalanine analogue also led to the production of novel fluorinated cycloaspeptides. The ditryptophenaline pathway from *Aspergillus flavus* was quickly confirmed as being a second example of such a system, suggesting that as more NRPS gene clusters are identified and characterised, further examples will be uncovered. Again, feeding a *N*-Met knock-out strain allowed the production of an unnatural ditryptophenaline analogue.

The preferential acceptance of methylated amino acids by an NRPS combined with the ability to remove the natural substrate supply provides a unique biotransformation opportunity over traditional feeding of modified amino acids. Firstly, methylated amino acids fed into the system have little competition from unmethylated cytoplasmic amino acids. Secondly, the incorporation will be more efficient because synthetic methylated amino acids are not at risk of being consumed by other cellular processes such as protein production.

The ability to produce natural product analogues is valuable due to the potential for altered bioactivities or pharmacokinetic properties. *N*-Methylated peptides are particularly desirable as they are known to often display improved stability over their non-methylated counterparts. Currently medicinal chemistry employs expensive and toxic reagents to synthesise *N*-methylated peptides. Consequently, new approaches for *de novo* biosynthesis of *N*-methylated peptides could be an attractive alternative to chemical synthesis. The ability to generate fluorinated natural product analogues is particularly relevant for natural product research due to their extreme rarity in nature (less than 0.005% of identified natural products contain fluorine^18^) combined with the various benefits observed with compounds containing one or more fluorine atom,^19^,^20^ demonstrated by the fact that 15–20% of pharmaceuticals now contain at least one fluorine atom.^18^ Such compounds could also be more amenable to semi-synthetic derivatization, which is a major route to drug development.

Using A-domains which accept methylated amino acids in domain swaps could also be used to introduce methylated amino acids, or unnatural analogues such as the fluorinated amino acids into other NRP natural products of agricultural of pharmaceutical interest. Indeed, there have been some major advances in NRPS structural biology and engineering recently,^21^–^23^ which means that the prospect of swapping in domains/modules to introduce *N*-methylated amino acids into existing NRPs could become feasible. Detailed *in vitro* studies of the *N*-MeT enzyme and the *N*–Me amino acid activating A-domains and modules, including crystallography, could help to elucidate where the selectivity and specificity of such systems lie, and the roles that the individual NRPS domains play in controlling the output of such systems. In the longer term, such an understanding could help guide engineering to alter the substrate specificity of these systems, enabling methylation and incorporation of a wider range of *N*-methylated amino acids. Also, further genome sequencing and mining could lead to the discovery of other *N*-MeT and NRPS that incorporate different *N*-methylated amino acids in nature. Taken together, such studies could provide a set of *N*-Met enzymes and NRPS domains/modules for engineering the *de novo* biosynthesis of *N*-methylated peptide ‘non-natural’ products through the assembly of novel chimeric NRPS.

## Conflicts of interest

There are no conflicts to declare.

## Supplementary Material

Supplementary information
