# Supplementary material for: The cycloaspeptides: uncovering a new model for methylated nonribosomal peptide biosynthesis
Source: Chem Sci. 2018 Apr 10;9(17):4109–17. doi: 10.1039/c8sc00717a (PMC5941284; doi:10.1039/c8sc00717a)
Supplement: Supplementary file 1 [file SC-009-C8SC00717A-s001.pdf]

Electronic Supplementary Material (ESI) for Chemical Science.
This journal is © The Royal Society of Chemistry 2018

Experimental and Supporting Information for:

The Cycloaspeptides: Uncovering a new model for methylated nonribosomal peptide biosynthesis.

Kate M. J. de Mattos-Shipley,[a*] Claudio Greco,[a] David M. Heard,[a] Gemma Hough,[b] Nicholas P. Mulholland,[b] Jason L. Vincent,[b] Jason Micklefield,[c] Thomas J. Simpson,[a] Christine L. Willis,[a] Russell J Cox[d,e] and Andrew M. Bailey[f]

a. School of Chemistry, University of Bristol, Cantock's Close Bristol, BS8 1TS, UK
b. Syngenta Ltd., Jealott's Hill International Research Centre Bracknell, Berkshire, RG42 6EY, UK
c. School of Chemistry, University of Manchester, Oxford Road, Manchester
d. Institute für Organsche Chemie, Leibniz Universität Hannover, Schneiderberg 1A, 30167 Hannover, Germany
e. BMWZ, Leibniz Universität Hannover, Schneiderberg 38, 30167 Hannover, Germany
f. School of Biological Sciences, Life Sciences Building, University of Bristol, 24 Tyndall Avenue, Bristol, BS8 1TQ, UK

Corresponding authors: Andy.Bailey@bristol.ac.uk, Kate.deMattos-Shipley@bristol.ac.uk

Contents

Experimental.......... 5

Strains.......... 5

Culture conditions.......... 5

Chemical extractions.......... 5

LCMS analysis and purification.......... 6

Quantification of cycloaspeptide A.......... 7

Nucleic acid preparation.......... 7

PCR.......... 8

Primer Table.......... 8

Transformations protocols.......... 10

S. cerevisiae.......... 10

A. oryzae.......... 10

P. soppii.......... 10

Gene knock-outs.......... 10

H. virescens injection assay.......... 13

Genome Sequencing and Bioinformatics.......... 13

Identification of homologues to known clusters.......... 14

Identification and analysis of the cycloaspeptide gene cluster.......... 17

Construction of plasmids.......... 21

pTHPs-eGFP.......... 21

pTYGS-N-Met-NRPS.......... 22

pTYGen-N-Met-NRPS.......... 23

LCMS data.......... 24

Orbitrap LCMS data 32

Pencillium species 32

A. flavus 35

Isolated compounds 36

General Experimental 36

Cycloaspeptide A **1** 36

Cycloaspeptide E **5** 38

Pseurotin A **8** 45

Synthesis of Amino Acids 47

General Experimental 47

(±)-N-(tert-butoxycarbonyl)-N-methyl-p-fluorophenylalanine S2 47

(±)-N-(tert-butoxycarbonyl)-N-methyl-m-fluorophenylalanine S3 48

(±)-N-(tert-butoxycarbonyl)-N-methyl-o-fluorophenylalanine S4 49

(±)-N-methyl-p-fluorophenylalanine 23 49

(±)-N-methyl-m-fluorophenylalanine 22 50

(±)-N-methyl-o-fluorophenylalanine 21 50

(±)-N-methyl-p-methylphenylalanine 18 50

(S)-(-)-N-(tert-butoxycarbonyl)-N-methyl-p-fluorophenylalanine (S)-S2 51

(S)-(+)-N-methyl-p-fluorophenylalanine (S)-23 52

(S)-(+)-N-ethylphenylalanine 19 52

(S)-(-)-N-(tert-butoxycarbonyl)-N,O-dimethyltyrosine (S)-S9 53

(S)-(+)- N,O-dimethyltyrosine 20 53

4-Fluoro-N-(tert-butoxycarbonyl)-N-methylphenylalanine 54

4-Fluoro-N-methylphenylalanine 56

3-Fluoro-N-(tert-butoxycarbonyl)-N-methylphenylalanine 57

3-Fluoro-N-methylphenylalanine 59

2-Fluoro-N-(tert-butoxycarbonyl)-N-methylphenylalanine 60

2-Fluoro-N-methylphenylalanine 62

(±)-N-methyl-p-methylphenylalanine 64

(S)-(+)-N-ethylphenylalanine 65

(S)-(-)-N-(tert-butoxycarbonyl)-N,O-dimethyltyrosine 67

(S)-(+)- N,O-dimethyltyrosine 68

H. virescens injection assay 70

References 71

Experimental

Strains

Penicillium soppii (CBS 869.70), *Penicillium jamesonlandense* (CBS 102888) were obtained from CBS. *A. oryzae* NSAR1 had previously been obtained as a gift from the Kitamoto group.[1] *Saccharomyces cerevisiae* strain YPH499 (Stratagene) was used for the construction of plasmids via yeast homologous recombination. *Escherichia coli* strain TOP10 (Invitrogen) was used for the standard propagation of plasmids, except when propagating gateway destination vectors when One Shot® ccdB Survival™ 2 T1R Competent Cells (ThermoFisher) were used.

Culture conditions

Both *Penicillium* strains were maintained on MEA at 25 ⁰C. A range of media were originally used to screen metabolite production in both strains including CYB (3.5 % Czapek-Dox Broth, 1 % Yeast Extract), MEB (Malt Extract Broth) and YEB (Yeast Extract Broth). Solid media of the above broths were also used, with the addition of 1.5 % agar. All subsequent work was done with *P. soppii* using CYB as the production media, cultured in 50 ml of CYB in 250 ml flasks at 25 ⁰C with shaking at 200 rpm for 7 days.

A. oryzae NSAR1 was maintained on MEA at 28 ⁰C. Induction medium for *A. oryzae* transformants was 100 ml CMP in 500 ml flasks (3.5% w/v Czapek Dox Broth, 2% w/v D-(+)-maltose monohydrate, 1% w/v peptone) at 28 °C with shaking at 200 rpm for 7 days.

Chemical extractions

From plates

Initial extractions from agar plates were based on the protocol reported by Smedsgaard.[2] 1 cm^2 plugs of agar from the rim and the centre of growing colonies were treated with a solution of ethyl acetate, dichloromethane and methanol (3:2:1) with 1 % acetic acid. After 1 hour in an ultrasonic bath, the liquid was removed and concentrated

under a nitrogen stream. The crude extract was then dissolved in acetonitrile to a concentration of 10 mg/mL and analysed by HPLC-MS/UV/ELSD.

From liquid cultures

Typical chemical extractions from fermentation cultures, both *Penicillium* and *A. oryzae*, involved adding an equal volume of ethyl acetate and blending to both break open the cells and thoroughly mix the culture with the solvent. The ethyl acetate phase was then separated, dried (using $MgSO_4$) and evaporated under reduced pressure to give a crude extract. This was then dissolved in acetonitrile to a concentration of 10 mg/mL and analysed by HPLC-MS/UV/ELSD.

LCMS analysis and purification

LC-MS analysis of crude fungal extracts was conducted using a Waters LCMS system comprising of a Waters 2767 autosampler, Waters 2545 pump system, a Phenomenex Kinetex column (2.6 μ, C18, 100 Å, 4.6 × 100 mm) with a Phenomenex Security Guard precolumn (Luna C5 300 Å) eluted at 1 mL/min. Detection was by Waters 2998 Diode Array detector between 200 and 600 nm; Waters 2424 ELSD and Waters SQD-2 mass detector operating simultaneously in ES+ and ES- modes between 100 m/z and 800 m/z. A gradient of solvents was used (A, HPLC grade H2O containing 0.05% formic acid; B, HPLC grade CH_3CN containing 0.05% formic acid) comprising: 0 min, 95 % A; 1 min, 95% A; 2 min, 60 % A; 15 min, 5% A; 17 min, 5% A; 18 min, 95% A; 20 min, 95% A; flow rate 1 mL·min-1

Purification of compounds was generally achieved using a Waters mass-directed autopurification system consisting of a Waters 2767 autosampler, Waters 2545 pump system, a Phenomenex Kinetex Axia column (5μ, C18, 100 Å, 21.2 × 250 mm) equipped with a Phenomenex Security Guard precolumn (Luna C5 300 Å). Flow rate was at 16 mL· min-1. Solvent A: HPLC grade H_2O + 0.05% formic acid. Solvent B: HPLC grade CH_3CN + 0.05% formic acid. The post-column flow was split (100:1) and the minority flow was made up with solvent A to 1 mL· min-1 for simultaneous analysis by diode array detector (Waters 2998), evaporative light scattering (Waters 2424) and ESI mass spectrometry in positive and negative modes (Waters SQD-2). Detected peaks were collected, then combined fractions were evaporated under a flow of dry N_2 gas and residues dissolved directly in NMR solvent for NMR analysis.

Quantification of cycloaspeptide A

To allow for quantification of cycloaspeptide A, purified cycloaspeptide A was used to produce a standard curve. Cycloaspeptide A concentrations and the peak integration from diode array data were found to have a linear relationship within the range 0.01 – 1 mg / ml with an injection volume of 10 µl (Figure S1). Thus, crude extracts were diluted to give a concentration in the middle of this range and standards were injected on the same analytical run.

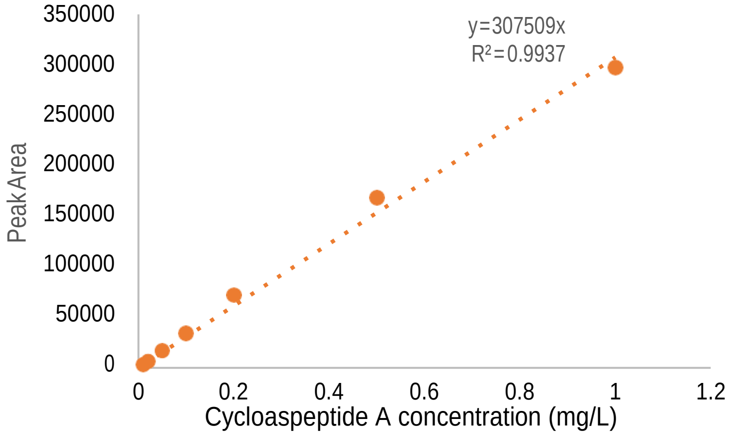

Figure S1: The relationship between cycloaspeptide A concentration and peak area (UV chromatogram) was found to be linear within the concentration range of 0.01 – 1 mg / ml when the injection volume was 10 µl.

Nucleic acid preparation

Genomic DNA for sequencing and for strain analysis was prepared from *P. jamesonlandense* and *P. soppii* by harvesting mycelia which was then lyophilized and ground under liquid nitrogen before using the GenElute Plant Genomic DNA Miniprep kit (Sigma). RNA for cDNA production was prepared using the RNeasy kit from Qiagen according to the manufacturer's instructions. cDNA was synthesised using RevertAid First Strand cDNA Synthesis Kit (ThermoFisher).

PCR

Analytical PCR was performed using BioMix Red (Bioline) and preparative PCR was performed the high-fidelity polymerases KOD Hot Start DNA Polymerase (Merck Millipore) or KAPA HiFi DNA polymerase (Roche Diagnostics).

Primer Table

Table S1: All primers were synthesised by Sigma.

**Primer Name**	**Sequence 5' – 3'**
**pTub-Ps-F**	CTTGTCGGTCTTGGGCGCACCGGCGGACATACTCCAAATGCCACCTCCAA
**pTub-Ps-R**	GGTGAACAGCTCCTCGCCCTTGCTCACCATTTTGAAAAGGTATCCCAATG
**pGpdA-Ps-F**	TTGAGATTGATTGGAGGTGGCATTTGGAGTATGTCCGCCGGTGCGCCCAA
**pGpdA-Ps-R**	CTCGACAGACGTCGCGGTGAGTTCAGGCATGATTGCGGTTTACTAGAGCA
**Yeast-frag-F**	CACGACGTTGTAAAACGACGGCACGTGCCACCATTGCGAATACCGCTTCC
**Yeast-frag-R**	TAATTATATCAGTTATTACCTGCATGCCTGCAGGTCGAGTGGAGATGTGG
**YA-PscyB-F1**	TAATGCCAACTTTGTACAAAAAAGCAGGCTATGTCTCAGATCAAGGTCCGA
**YA-PscyB-F2**	TGGAGTCTTGTCCTATCTGC
**YA-PscyB-F3**	CCAGATGATTGATACGGCTT
**YA-PscyB-F4**	ACCGGATTCTCGGACAGTTC
**YA-PscyB-F5**	CCATCTTCCTGATCCTAGCC
**YA-PscyB-R1**	TTCCCGTCAGAGACAAATCC
**YA-PscyB-R2**	TTCTGTAGCCCGGTCTGTTC
**YA-PscyB-R3**	ACGGACTTGTTCCAAATGG
**YA-PscyB-R4**	GCATGTTGCAGCACAAGGTC
**YA-PscyB-R5**	TATAATGCCAACTTTGTACAAGAAAGCTGGTTGTCTCATCCTCAGCCGTT
**YA-PscyA-F**	CTTTCTTTCAACACAAGATCCCAAAGTCAAAATGCGGTACCTCACTCAAC
**YA-PscyA-R**	AGGTTGGCTGGTAGACGTCATATAATCATATCAGCGCGAAGCGGGATTGG
**pTYGen-G418-F**	GTGAAAGTAAAAGATGCTGAAGATCAGTTGTCTAGTGGATCTTTCGACAC

**pTYGen-G418-R**	TTGGAGGTGGCATTTGGAGTTCGAGTGGAGATGTGGAGTG
**pTYGen-Tub-F**	CACTCCACATCTCCACTCGAACTCCAAATGCCACCTCCAA
**pTYGen-Tub-R**	GCTGTGTTGTCGGACCTTGATCTGAGACATTTTGAAAAGGTATCCCAATG
**pTYGen-2u-R**	ACTGATTACTAGCGAAGCTG
**PscyD-LF**	TAATGCCAACTTTGTACAAAAAAGCAGGCTTATTCAAGGGTGCCTAGTGA
**PscyD-LR**	CGAAAGATCCACTAGAGGATCCCCATCATGCTCTGGTGAAGAGATGACAG
**PscyD-RF**	AGCGCCCACTCCACATCTCCACTCGACCTGCGGATCTCATGGGCTGTCA
**PscyD-RR**	AATGCCAACTTTGTACAAGAAAGCTGGGTTTGGACGGGTGTTTGCAATG
**PscyB-LF**	TAATGCCAACTTTGTACAAAAAAGCAGGCTATGTCTCAGATCAAGGTCCGA
**PscyB-LR**	ACTAGAGGATCCCCATCATGGAAAGACGACCAGAATTGGCA
**PscyB-RF**	CCACATCTCCACTCGACCTGTCGGAAGGATACACAAGTCAA
**PscyB-RR**	AATGCCAACTTTGTACAAGAAAGCTGGGTTCAGCCGTTAGTTAGGTCATG
**PscyA-LF**	TAATGCCAACTTTGTACAAAAAAGCAGGCTGCAGCCACTTAACTATGTAC
**PscyA-LR**	ACTAGAGGATCCCCATCATGACTAACCCAGACCCAAGTTC
**PscyA-RF**	CCACATCTCCACTCGACCTGGCGAATACGGTTATGGGACA
**PscyA-RR**	AATGCCAACTTTGTACAAGAAAGCTGGGTAGTCTGTTTCTCATTTGAGCC
**HygR1-F**	CATGATGGGGATCCTCTAGTG
**HygR2-R**	CTCCAACAATGTCCTGACG
**HygR3-F**	CTGTCGAGAAGTTTCTGATCG
**HygR4-R**	CACATCTCCACTCGACCTG
**PscyB-KOA-L**	GCATCAATTGGGCAATCG
**PscyB-KOA-R**	TGGGACCTGTCTTGTCTC
**PscyA-KOA-L**	ATTGTCAGCCGGTCTACCTC
**PscyA-KOA-R**	TTCTCGTCACTCTGGACAGT
**PscyD-KOA-L**	GGATTCTGCGTTGAGAGCAA
**PscyD-KOA-R**	TTGACATTGGCATCAACGAT

Transformations protocols

S. cerevisiae

Transformations were performed using the LiAc PEG mediated method.[3]

A. oryzae

Transformations were performed using the PEG mediated protoplast method as described previously.[4]

P. soppii

Spores were harvested from a single, fully covered plate and incubated for 3 – 5 days at 25 °C with shaking at 200 rpm in 50 ml of MEB medium (Malt Extract Broth). The resulting hyphae were harvested by centrifugation (8000 x g for 10 mins), washed once with H_2O, once with 0.8M NaCl then resuspended in 10 ml of filter sterilised protoplasting solution (20 mg/ml Trichoderma lysing enzyme (Sigma) and 5 mg/ml driselase (Sigma) in 0.8 M NaCl). Protoplasting was left to proceed with gentle shaking for 2 hours. Protoplasts were filtered through sterile miracloth, harvested by centrifugation (3000 x g for 5 min) and washed in solution 1 (0.8 M NaCl, 10 mM CaCl2, 50 mM Tris-HCl pH 7.5). The protoplasts were then resuspended in 200-500 µl of solution 1. For each transformation, 100 µl of protoplasts and 5-10 µg (10 µl max) of concentrated PCR product or plasmid DNA were gently mixed. The transformation was incubated on ice for 2 mins, after which 1 ml of solution 2 (60% (w/v) PEG 3350, 0.8 M NaCl, 10 mM CaCl2, 50 mM Tris-HCl pH 7.5) was added and then incubated at room temperature for 20 mins. To plate out, 5 ml of molten (50 °C) MEA/S top medium (2% (w/v) malt extract broth, 1 M sorbitol, 0.8% (w/v) agar) was added, gently mixed and then overlaid onto prepared plates (2% (w/v) malt extract broth, 1 M sorbitol, 1.5% (w/v) agar). Plates were then incubated at 28 °C for 3 days, or until colonies appeared.

Gene knock-outs

Various genes were disrupted in this work using the bipartite gene knock-out approach. This approach involves splitting the selectable marker into two overlapping fragments, each of which are fused to regions homologous to the region to be targeted. (with a ~500 bp overlap). The splitting of the selectable marker means that homologous recombination is required to reconstruct the cassette and allow selection, which in turn improve the frequency of integration via homologous recombination, and thus gene disruption.

Gene knock-out cassettes were constructed in plasmid pE-YA and subsequently used as templates to produce the DNA fragments required for the bipartite knock-out method. The general approach is outlined in Figure S2.

Three knock-out cassettes were constructed for the cycloaspeptide *N*-methyltransferase *PscyA*, NRPS *PscyB* and the transcription factor *PscyD*. The right and left flanking regions for each were amplified from *P. soppii* genomic DNA using the following primer pairs:

PscyB-LF/PscyB-LR and PscyB-RF/PscyB-RR for the left and right flanking regions of PscyB.

PscyA-LF/PscyA-LR and PscyA-RF/PscyA-RR for the left and right flanking regions of PscyA.

PscyD-LF/PscyD-LR and PscyD-RF/PscyD-RR for the left and right flanking regions of PscyD.

The primer pair HygR1-F/HygR4-R was used to amplify the hygromycin cassette which formed the centre of the knock-out construct. To amplify the bipartite fragments for the transformation, the forward primer for the left flanking region (PscyA-LF, PscyB-LF or PscyD-LF) was combined with the primer HygR2-R, and HygR3-F was combined with the reverse primer for the right flanking region (PscyA-RR, PscyB-RR or PscyD-RR).

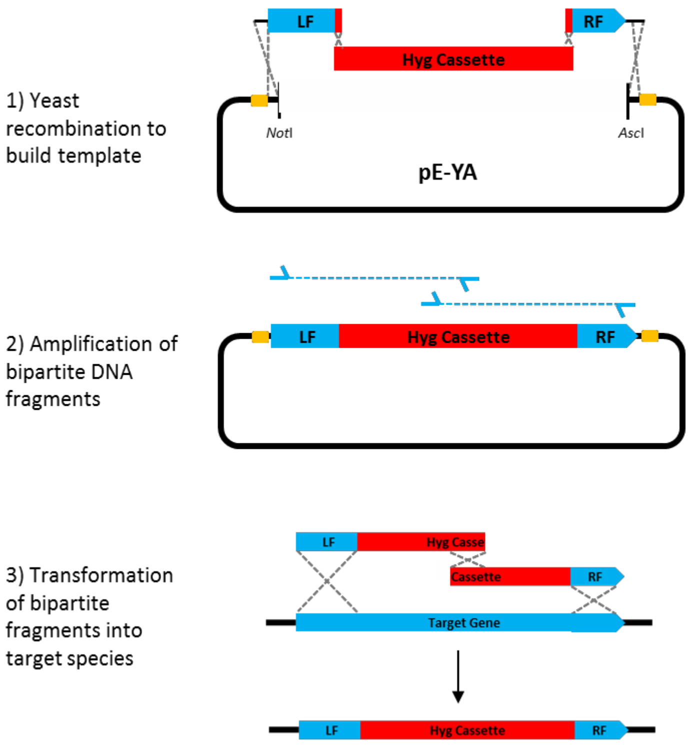

Figure S2: The knockout cassettes consist of the hygromycin resistance cassette, which consists of the gpdA promoter, the HygR resistance gene and the trpC terminator (reference), flanked by approximately 1 kb fragments of the gene to be

targeted. Once this cassette has been constructed in yeast, the overlapping bipartite fragment can be amplified from the resulting plasmid, either directly from the yeast plasmid preparation, or after passage through *E. coli*.

Once transformants had been generated using the bipartite method, targeted integration of the knock-out construct was tested by using primers designed to bind outside the homologous regions in combination with primers which bind within resistance cassette (Figure S3). Correct integration at the left-hand side of the targeted region was tested using the primer HygR2-R in combination with either PscyA-KOA-L, PscyB-KOA-L or PscyD-KOA-L. Correct integration at the right-hand end of the region was tested using primer HygR3-F with the gene specific primers PscyA-KOA-R, PscyB-KOA-R or PscyD-KOA-R.

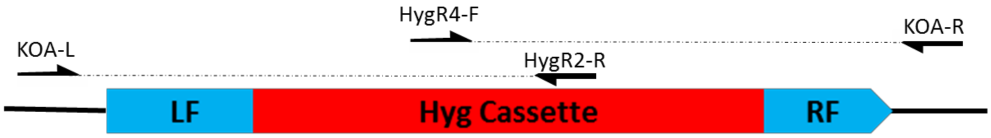

Figure S3: Correct integration of knock-out cassettes was confirmed by combining primers designed to bind outside the targeted region with primers binding within the resistance cassette.

H. virescens injection assay

Intrinsic insecticidal activity was determined by administering peptides via injection (33 gauge needle) into the haemolymph of early fourth instar tobacco budworm, *Heliothis virescens*. The injection was made into the second abdominal segment between the subdorsal band and spiracular band.

Insecticidal activity of the peptides was compared to Spinosad (positive control) and neat dimethyl sulfoxide DMSO (negative control). There were six replicates per treatment. 1µl of each treatment was injected per larva.

Cycloaspeptide E **5** and 4-fluoro phenylalanine Cycloaspeptide E were solubilised in DMSO at 10 mg ml^{-1}. Spinosad was solubilised in DMSO at 0.19 mg ml^{-1}.

Visual assessments of mortality were made at 1, 2, 3 and 7 days after injection for each treatment.

Genome Sequencing and Bioinformatics

Genome sequences were obtained for both *Penicillium* strains using Illumina MiSeq with a 600-cycle (2x300 bp) kit, and assembled and annotated using Newbler v29. The statistics for the two resulting genome sequences are shown in Table S2.

Table S2: Genome statistics

	Penicillium jamesonlandense	***Penicillium soppii***
**Estimated Genome Size**	38.1 Mbp	33.4 Mbp
**Number of Scaffolds**	235	300
**Largest Scaffold**	3,085,890	1,305,324
**Scaffold N50**	1,142,055	451,138
**Total Scaffold Length**	35.8 Mbp	32.6 Mbp
**Number of Contigs**	697	687
**Largest Contig**	1,181,107	788,536
**Contig N50**	376,897	276,214
**Total Contig Length**	35.8 Mbp	32.7 Mbp
**Mapping-rate**	94.0 %	97.5 %

An initial AntiSMASH[5] analysis was conducted on both genomes to identify putative secondary metabolite gene clusters (Table S3).

Table S3: The number of gene clusters of different types within the genomes of *P. soppii* and *P. jamesonlandense,* as analysed by antiSMASH.

**CLUSTER TYPE**	***P. SOPPII***	***P. JAMESONLANDENSE***
NRPS	20	17
PKS	16	13
PKS-NRPS	6	6
Terpene	4	2
Terpene-PKS-NRPS	1	0
Terpene-NRPS	0	1
Other	17	14
Putative	18	30
Total	82	83

Identification of homologues to known clusters

In addition to searching the genomes of *P. soppii* and *P. jamesonlandense* for putative cycloaspeptide clusters, homologues to the clusters known to be involved in other compounds were searched for, including the pseurotin A, fumagillin, griseofulvin and kojic acid clusters.

This was done by blasting our genomic databases for homologues to biosynthetic genes previously reported and available through NCBI. The results of those blast searches were cross-referenced with the antiSMASH predictions for each genome and putative gene clusters were then fully annotated in Artemis using FGENESH predictions and manual editing based on alignments with known homologues (Figures S4 –S6).

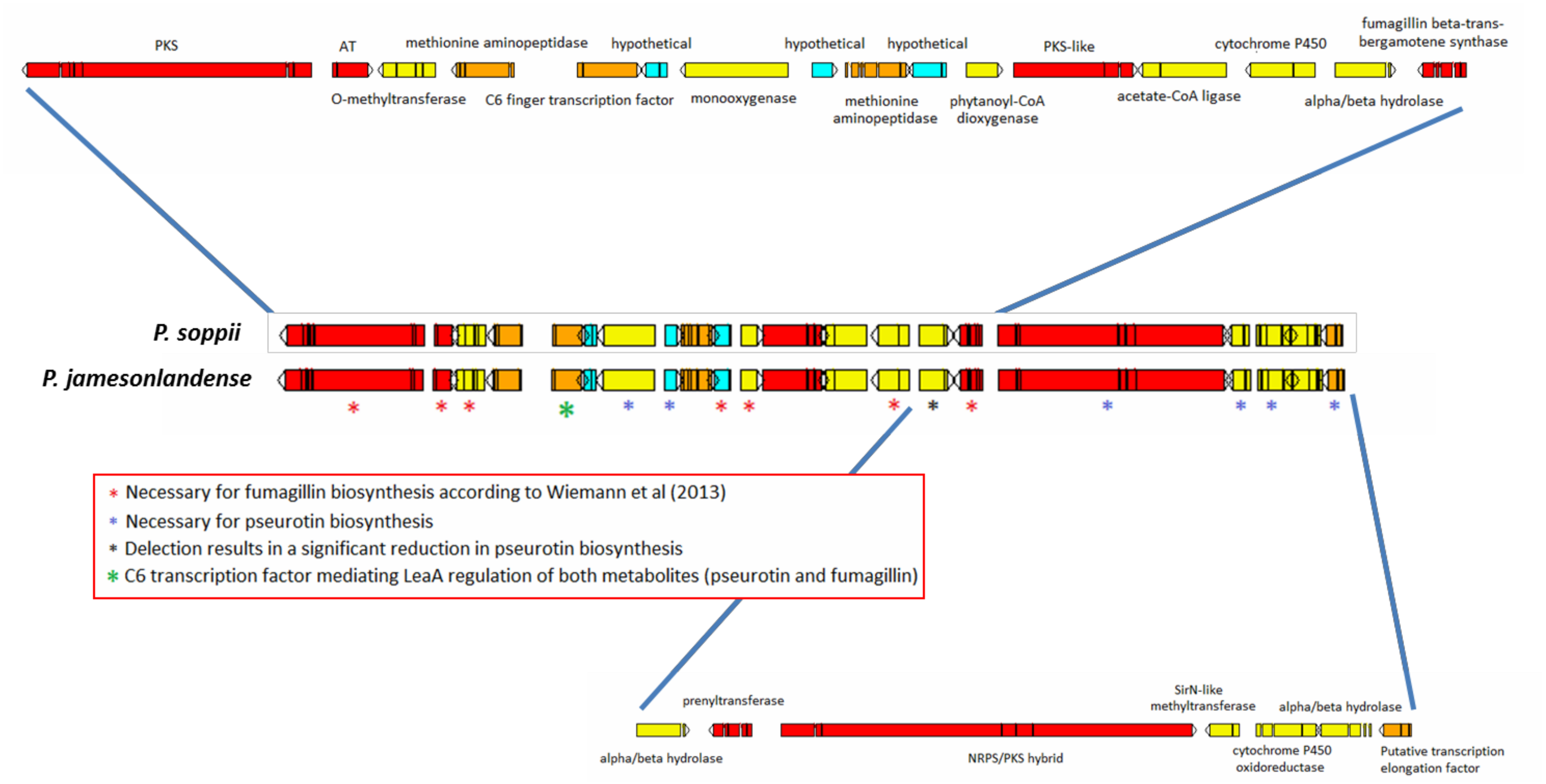

Figure S4: The pseurotin and fumagillin superclusters identified within the genomes of *P. soppii* and *P. jamesonlandense*. These clusters are homologous to the gene cluster identified in *A. fumigatus*[6], in both gene content and gene organisation.

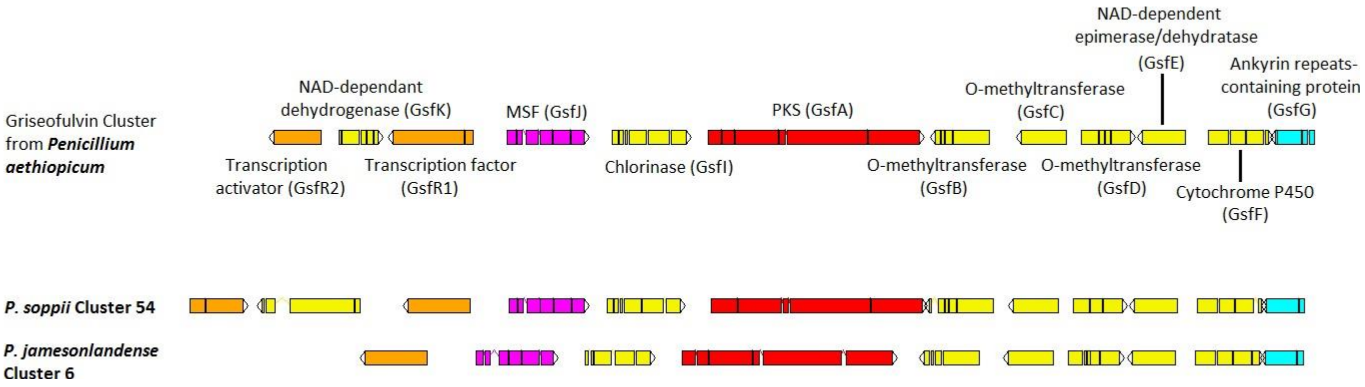

Figure S5: Homologues for the griseofulvin gene cluster from *Penicillium aethiopicum*[7] were identified within the genomes of *P. soppii* and *P. jamesonlandense*. The *P. jamesonlandense* gene cluster is lacking homologues to *GsfK*, a dehydrogenase encoding gene, and *GsfR2*, a transcription activator-encoding gene. We have not observed Griseofulvin production in this strain so it is possible that this cluster is no longer functional.

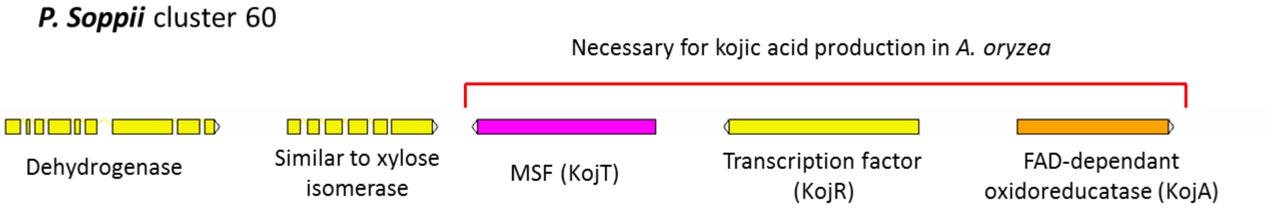

Figure S6: A putative kojic acid gene cluster was identified within the genome of *P. soppii*, but not *P. jamesonlandense*. Homologues to the known Kojic acid genes of *A. oryzae*; *KojT*, *KojR* and *KojA*,[8] were identified. In addition, two genes which may be involved in secondary metabolite biosynthesis; an isomerase and dehydrogenase, were identified adjacent.

Identification and analysis of the cycloaspeptide gene cluster

The domain structures of all NRPS encoding genes in the two *Penicillium* genomes were analysed using InterPro (EMBL) (Table S4). This identified one NRPS (*NRPS11*) as being common to both species and having the correct number of modules for producing a pentapeptide (Figure S7).

Table S4: Domains present in predicted NRPSs encoded by genes within the genomes of *P. soppii* and *P. jamesonlandense*. A total of 11 NRPS genes, present in 8 separate gene clusters, were common to both species. **A**: Adenylation domain. **C**: Condensation domain. **PCP**: Phosphopantetheine attachment site. **NR**: an NRPS domain (domain TIGR01720) though to be involved in post-condensation modification. **TE**: Termination thioesterase domain.

**GENE**	**CLUSTER**		**DOMAINS**				
NRPS1	***P. soppii***	***P. james.***	**A**	**PCP**	**C**	**NR**	**TE**
NRPS2	Cl5	Cl11	3	3	4	1	
NRPS3	Cl13	Cl15	2	2	2		
NRPS4	Cl20	Cl49	4	5	6	2	
NRPS5	Cl31	Cl44	1	1	-		1
NRPS6			2	2	1		1
NRPS7			2	2	2		
NRPS8	Cl62	Cl48	2	2	2		
NRPS9	Cl67	Cl47	3	5	6		
NRPS10	Cl68	Cl61	3	3	2		1
NRPS11	Cl82	Cl10	5	5	5		
NRPS12	Cl14	-	2	2	2		
NRPS13			1	1	1		
NRPS14	Cl21	-	1	2	2		
NRPS15	Cl24	-	2	2	2		
NRPS16	Cl27	-	4	4	5	1	
NRPS17	Cl36	-	2	2	2		
NRPS18	Cl56	-	4	4	6	1	
NRPS19	Cl59	-	4	4	5	1	
NRPS20	Cl73	-	2	2	2		
NRPS21	Cl76	-	4	4	5	1	

NRPS22	Cl78	-	3	3	3		
NRPS23	Cl80	-	1	3	3		
NRPS24	Cl81	-	3	3	3		
NRPS25	-	Cl7	4	4	6	1	
NRPS26	-	Cl22	4	5	6		1
NRPS27	-	Cl35	7	8	8		
NRPS28	-	Cl58	2	3	2		
NRPS29	-	Cl65	2	2	2		
NRPS30	-	Cl66	2	2	2		
NRPS31	-	Cl79	1	1	1		
NRPS32	-	Cl82	3	3	3		1
NRPS33	-	Cl83	1	1	1		

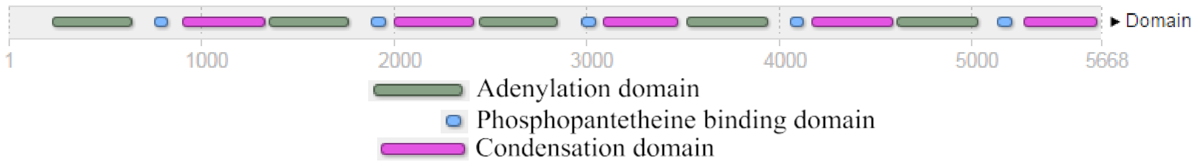

Figure S7: The predicted domain architecture of NRPS11, as analysed using InterPro[9]. This NRPS is present in both the *P. jamesonlandense* and *P. soppii* genomes, named *PjcyB* and *PscyB* respectively, and is responsible for cycloaspeptide biosynthesis.

The putative gene clusters containing *NRPS11* from both species were annotated using Softberry to predict the intron and exon positions for coding sequences. This annotation was then manually adjusted using discretion, based on alignments with homologues identified during blast searches. Putative gene functions were then investigated using BLAST and InterPro.

The antiSMASH predicted gene cluster for *P. soppii* contained only two genes, a putative *N*-methyltransferase encoding gene (named *PscyA*) and *NRPS11* (*PscyB*). The antiSMASH predicted cluster for *P. jamesonlandense* was significantly larger, containing homologues to *PscyA* and *PscyB* (named *PjcyA* and *PjcyB* respectively) and encoding a putative amidase (*PjcyC*), a transcription factor (*PjcyD*), an amino acid transporter (*PjcyF*) and an aminotransferase (*PjcyG*) (Figure S8). Searching the *P. soppii* genome for homologues to these additional genes identified them all together, but at a different genomic locus, with a genomic comparison conducted using artemis comparison tool suggesting that a genomic region containing the *N*-methyltransferase and NRPS (*PscyA* and

PscyB) has undergone a translocation event in *P. soppii* at some point during the species divergence. An additional gene, *PscyE*, encoding a putative dehydrogenase, was present in *P. soppii* but absent in *P. jamesonlandense*.

A bioinformatic analysis of the genes in both species is shown in Table S5. Closest homologues were identified using the blastp algorithm to search the Swiss-Prot protein database.

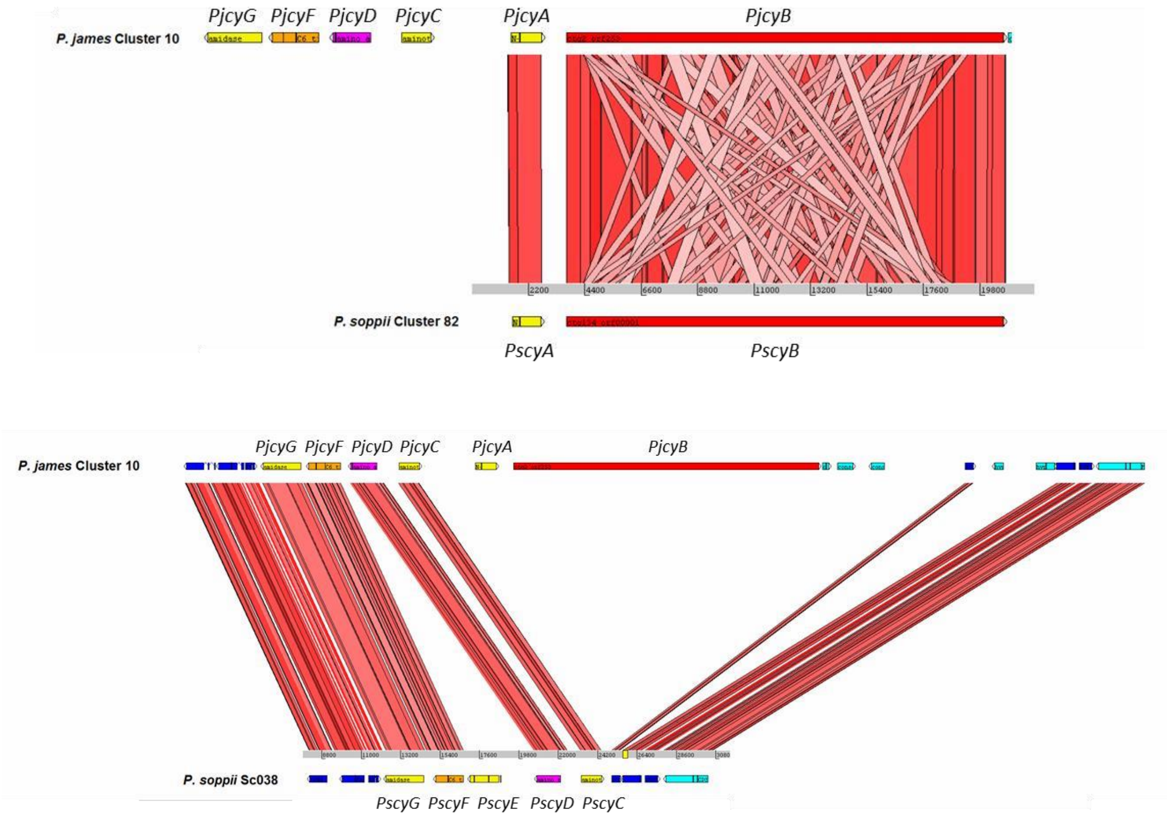

Figure S8: An ACT comparison of putative cycloaspeptide biosynthetic genes present in the genomes of *P. jamesonlandense* and *P. soppii*. Homology between the predicted protein sequences (using the tblastx algorithm) is denoted by the red bars. In *P. jamesonlandense* the 6 genes are all located at one locus. In *P. soppii* they are split over two loci.

Table S5: A bioinformatic analysis of the genes identified within the predicted cycloaspeptide gene clusters of *P. soppii* and *P. jamesonlandense*. Predicted gene function is based on homologues identified during BLAST searches. Domains were identified using the NCBI Conserved Domain Search.[10] The closest homologues, identified by blasting the predicted protein sequences against the Swiss-Prot database, are shown.

**Gene name (*P. james* / *P. soppii*)**	**Predicted gene function**	**Gene Size**	**Introns**	**Protein Size**	**Domains**	**Homologue**	**Identity (%)**	**E value**	**Query Coverage**	**Score**
PjcyA* / *PscyA	*N*-methyltransferase	1153/1096	1/1	366/347	Methyltransf_33: pfam10017	dtpB[11]	38%/39%	2e-82/2e-82	94%/96%	254/254
PjcyB* / *PscyB	NRPS	17013/17007	0/0	5670/5668	**See Figure S7**	easA[12]	33%/33%	0.0/0.0	97%/96%	2399 /2370
PjcyG* /*PscyG	Amidase	2079/2079	0/0	692/692	Amidase: pfam01425	-	-	-	-	-
PjcyD* / *PscyD	Transcription factor	1768/1777	2/2	559/556	GAL4: cd00067 Fungal_trans_2: pfam11951	ustR[13]	26%/25%	9e-44/1e-46	94%/98%	164/172
**- / *PscyE***	Dehydrogenase	1687	3	491	-	-	-	-	-	-
PjcyF* / *PscyF	Amino acid transporter	1416/1392	0/0	471/463	Aa_trans: pfam01490	mtr[14]	54%/53%	2e-162/2e-163	92%/94%	467/469
PjcyC* / *PscyC	Aminotransferase	1116/1116	0/0	371/371	Aminotran_1_2: pfam00155	His5[15]	28%/28%	2e-10/3e-10	52%/52%	62/61.2

Construction of plasmids

All plasmids used in this work were built by yeast recombination, where *S. cerevisiae* was transformed with approximately 0.5 µg of each required DNA fragment, with at least 30 bp homologous overlap between adjacent linear fragments to be joined. Resulting plasmids were extracted from yeast using the Zymoprep™ Yeast Plasmid Miniprep II kit, according to the manufacturer's instructions and then transformed into commercially available One Shot TOP10 Chemically Competent *E. coli* to obtain sufficient plasmid copies for downstream applications.

pTHPs-eGFP

This plasmid is an adapted version of pTYGS-eGFP, an *A. oryzae* plasmid provided by the group of Dr. Colin Lazarus, which contains a hygR cassette (conferring hygromycin resistance) as the selectable marker, and an eGFP cassette to function as a reporter gene. In the original plasmid, these cassettes contain the *A. oryzae* GpdA and AmyB promoters respectively. A strategy was designed to replace these promoters with the GpdA and tubulin promoters from *P. soppii*, which were amplified from genomic DNA using primers pGpdA-Ps-F/pGpdA-Ps-R and pTub-Ps-F/pTub-Ps-R respectively. A portion of the yeast shuttle vector, pE-YA, containing the 2u Ori and Ura3 cassette for uracil prototrophy, was also amplified using primers Yeast-frag-F/Yeast-frag-F and recombined into the plasmid, simultaneously converting the plasmid into a yeast/*E. coli* shuttle vector and allowing the yeast recombination to occur. pTYGS-eGFP was digested with HindIII, XhoI and NotI prior to recombination.

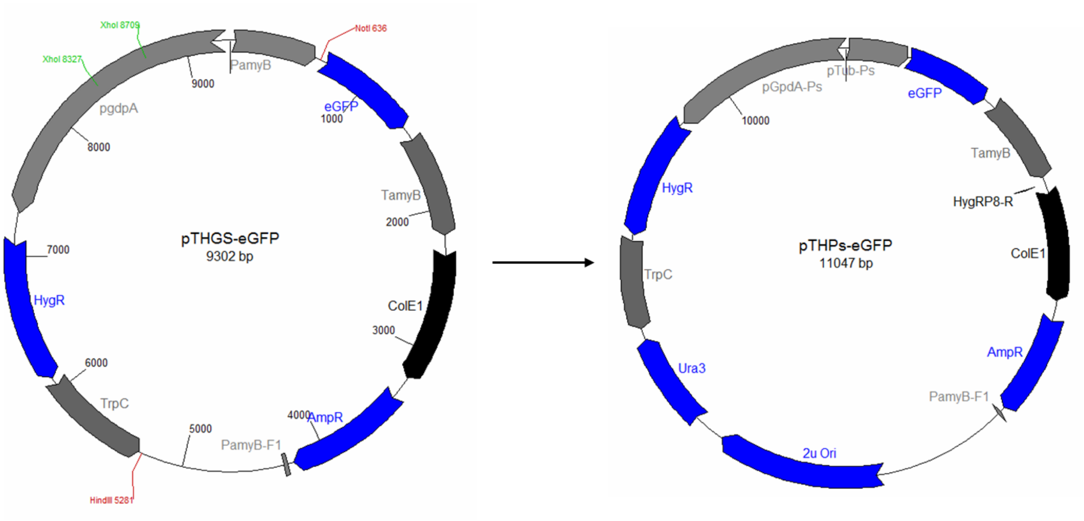

Figure S9: pTHGS-eGFP, an *A. oryzae* plasmid, was converted for use in *P. soppii* by replacing the promoter regions (pgpdA and pamyB) with native *P. soppii* promoters (pGpdA-Ps and pTub-Ps). This was achieved in one step by introducing the required cassettes for plasmid function in yeast (Ura3 and the 2u Ori) at the same time as swapping the promoters by yeast homologous recombination.

pTYGS-N-Met-NRPS

The large (17007 bp) NRPS encoding gene *PscyB* was amplified from genomic DNA in five overlapping fragments of approximately 3.5 kb each using the primer pairs YA-PscyB-F and YA-PscyB-R, numbered 1 to 5. These fragments were homologous recombined in yeast to reconstruct the full NRPS in the plasmid pE-YA. Simultaneously, the *N*-Methyltransferase *PscyA* was amplified using the primer pair YA-PscyA-F/YA-PscyA-R from *P. soppii* cDNA generated under cycloaspeptide production conditions (due to the presence of an intron). The resulting coding sequence was recombined into the multi-gene expression vector pTYGS-arg[16] so that it is located between an *A. oryzae* promoter (Padh) and terminator (Teno). *PscyB* was then transferred into the *PscyA* containing plasmid using gateway technology to produce the final vector: pTYGS-N-Met-NRPS (Figure S10).

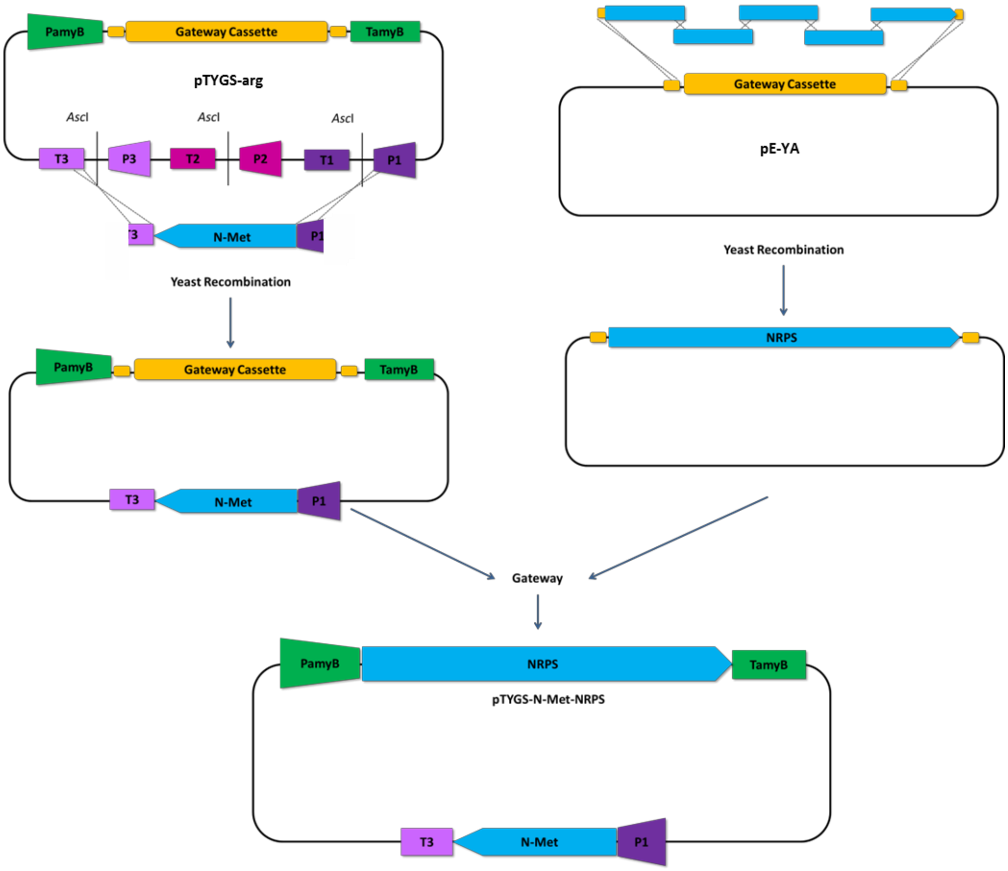

Figure S10: The construction of pTYGS-N-Met-NRPS. The *N*-methyltransferase *PscyA* was transferred, via yeast recombination, into a multigene expression vector with arginine prototrophy called pTYGS-Arg. This placed *PscyA* between the *A. oryzea* alcohol dehydrogenase promoter (pAdh) and the *A. oryzae* enolase terminator (tEno). The NRPS gene, having been reconstructed from 5 PCR products in a yeast assembly vector, pE-YA, was then transferred into the expression vector using Gateway technology.

pTYGen-N-Met-NRPS

To adapt pTYGS-N-Met-NRPS for use in the *P. soppii* NRPS knock-out strain, the argB cassette, which confers arginine prototrophy to *A. oryzae* NSAR1, was replaced with the nptII cassette, which confers resistance to the antibiotic geneticin. The promoter driving expression of the NRPS was also converted from the *A. oryzae* AmyB promoter to the *P. soppii* tubulin promoter. The nptII cassette was amplified from a geneticin plasmid using primers pTYGen-G418-F and pTYGen-G418-R. The tubulin promoter was amplified from plasmid pTYPs-eGFP using the primer pair pTYGen-Tub-F/pTYGen-Tub-R. SnaBI was used to digest pTYGS-N-Met-NRPS, but in addition to cutting within the region to be modified, this enzyme also cuts within the *N*-methyltransferase cassette and the 2u origin of replication. Therefore, a portion of the original plasmid spanning both of these restriction

sites was amplified using the primers YA-PscyA-F and pTYGen-2u-R, to act as a patch during the yeast recombination. These various fragments (SnaBI digested pTYGS-N-Met-NRPS, the geneticin cassette, the tubulin promoter and the patch fragment) were transformed into *S. cerevisiae* and the resulting plasmid, named pTYGen-N-Met-NRPS was extracted from yeast and propagated in *E. coli*.

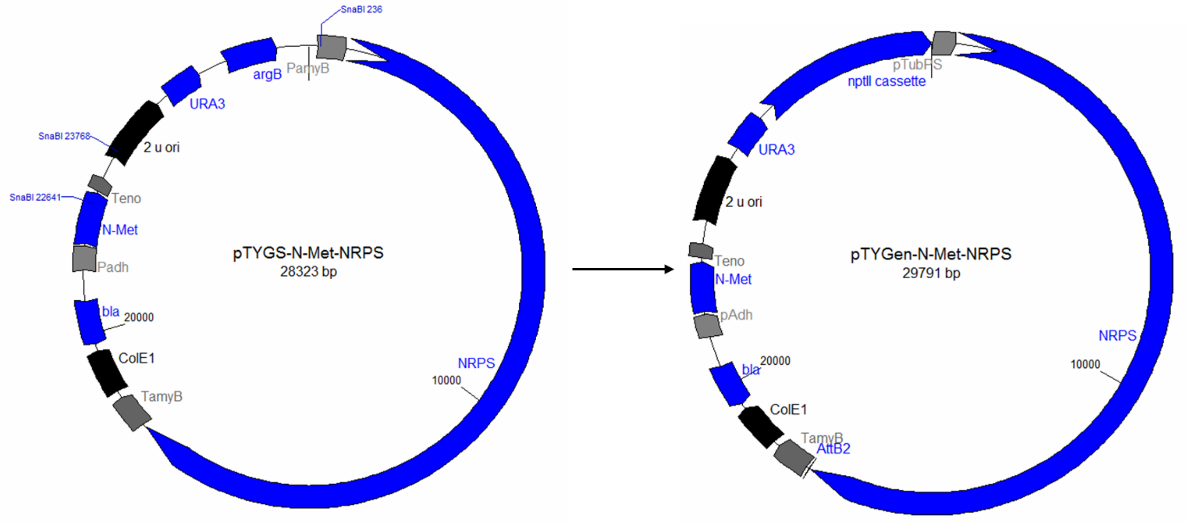

Figure S11: pTYGS-N-Met-NRPS, a plasmid constructed to produce cycloaspeptides in A. oryzae, was converted for use in P. soppii, to compliment an NRPS knock-out strain. This was achieved by replacing the argB cassette (for arginine prototrophy in *A. oryzae*), with the nptII cassette (for geneticin resistance), and replacing the *A. oryzae* amyB promoter with the *P. soppii* tubulin promoter.

LCMS data

Initial LCMS analysis using the Waters LCMS system with a Phenomenex Kinetex column allowed detection of compounds via their mass and UV spectra. Data from this analysis is shown below for the various cycloaspeptides and the ditryptophenaline compounds (Figures S12 to S20).

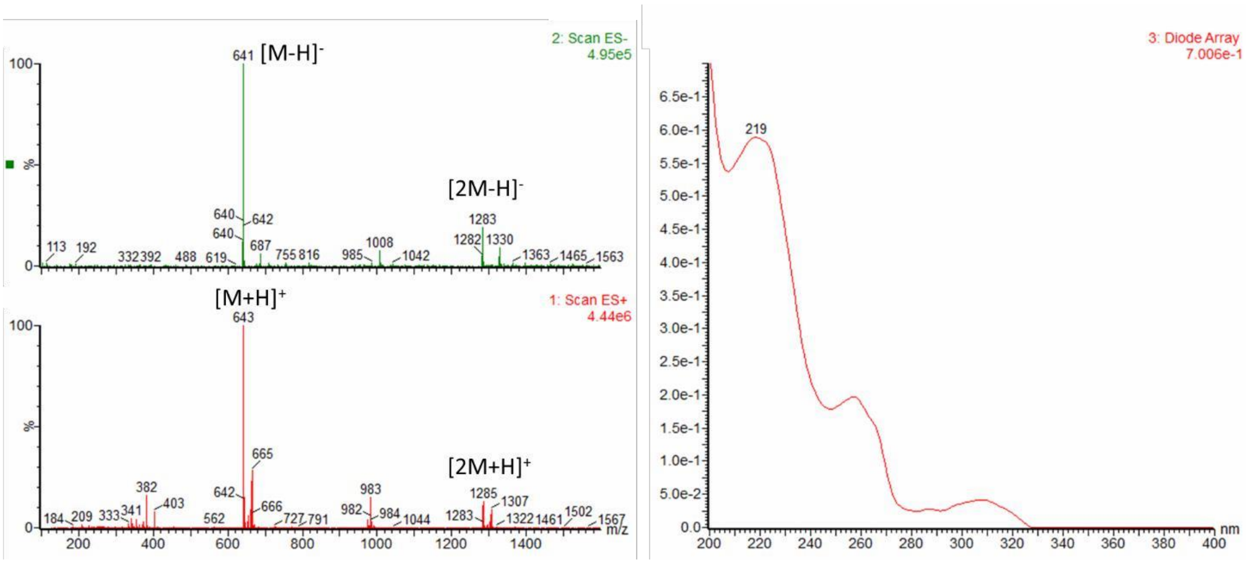

Figure S12: LCMS analysis of cycloaspeptide A **1** (molecular weight: 641.77) showing ES- and ES+ mass data and the UV absorbance.

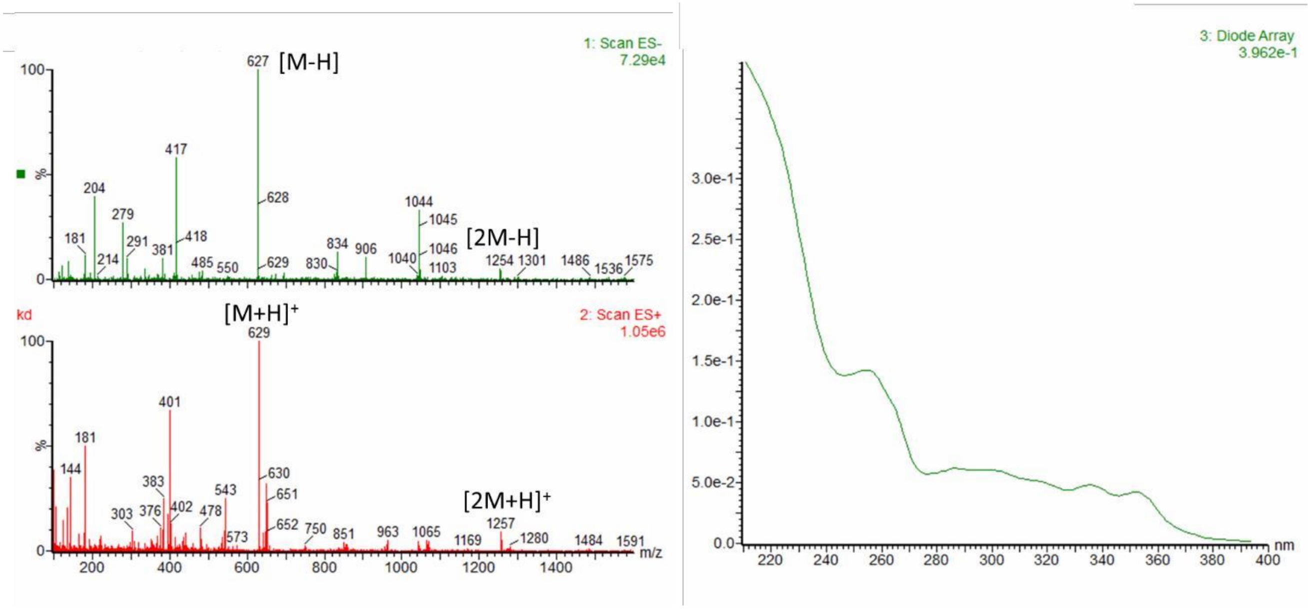

Figure S13: LCMS analysis of cycloaspeptide B **2** (molecular weight: 627.74) showing ES- and ES+ mass data and the UV absorbance.

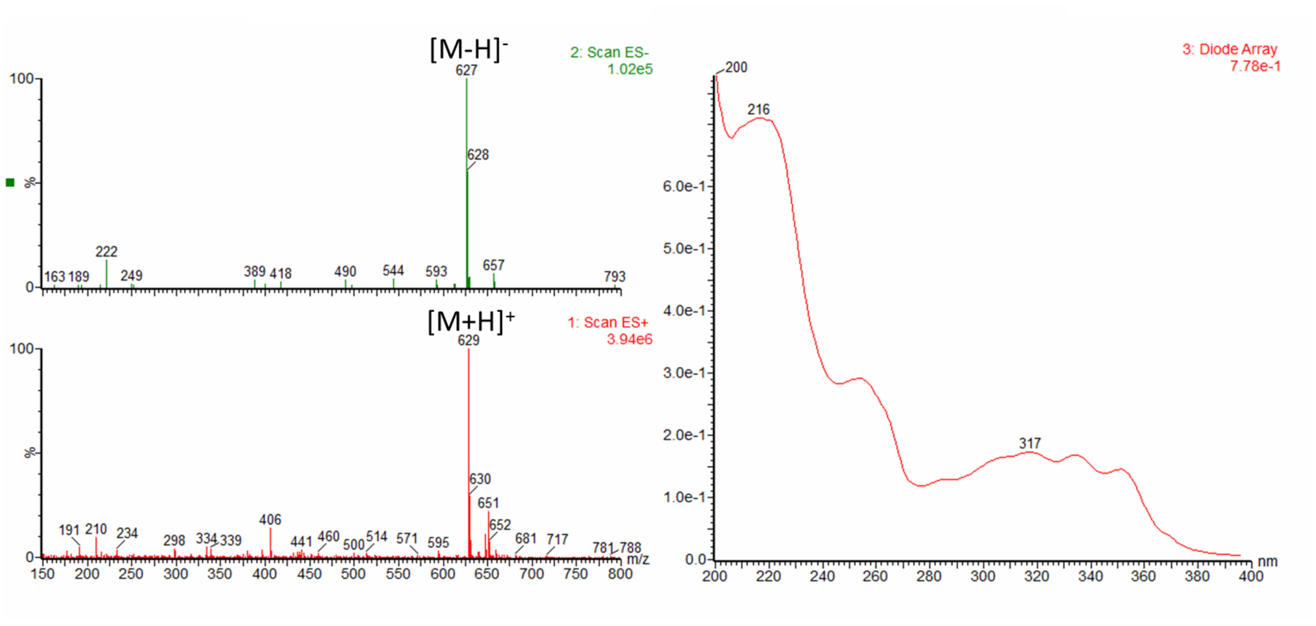

Figure S14: LCMS analysis of cycloaspeptide C **3** (molecular weight: 627.74) showing ES- and ES+ mass data and the UV absorbance.

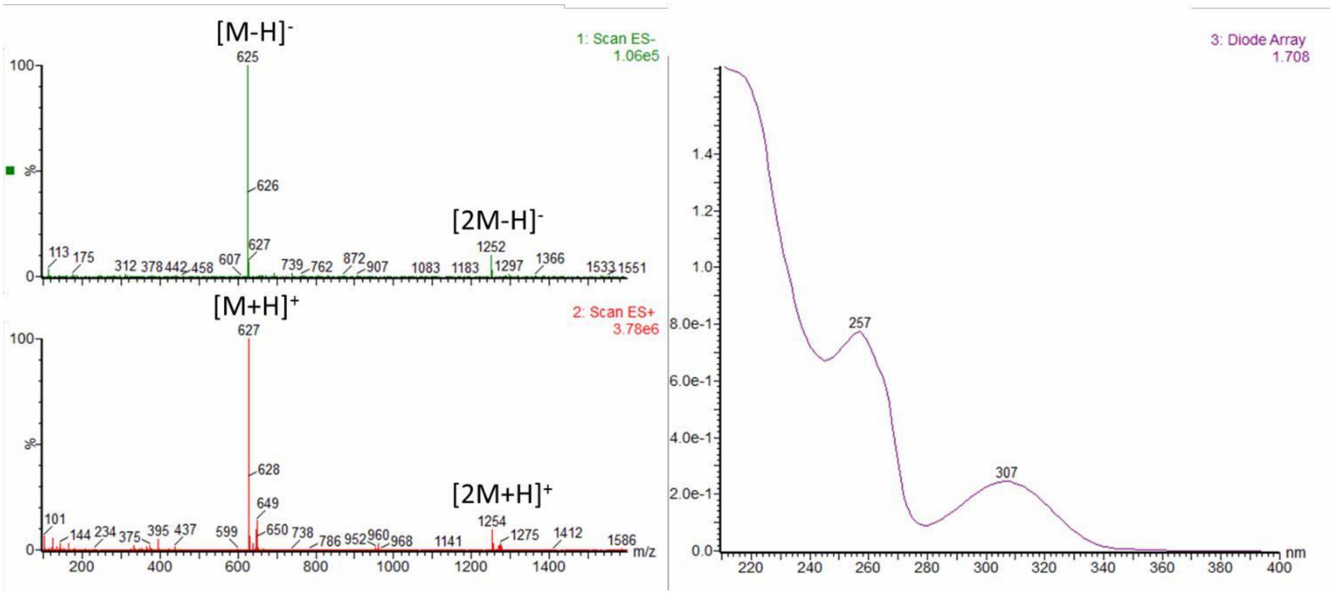

Figure S15: LCMS analysis of cycloaspeptide E **5** (molecular weight: 625.77) showing ES- and ES+ mass data and the UV absorbance.

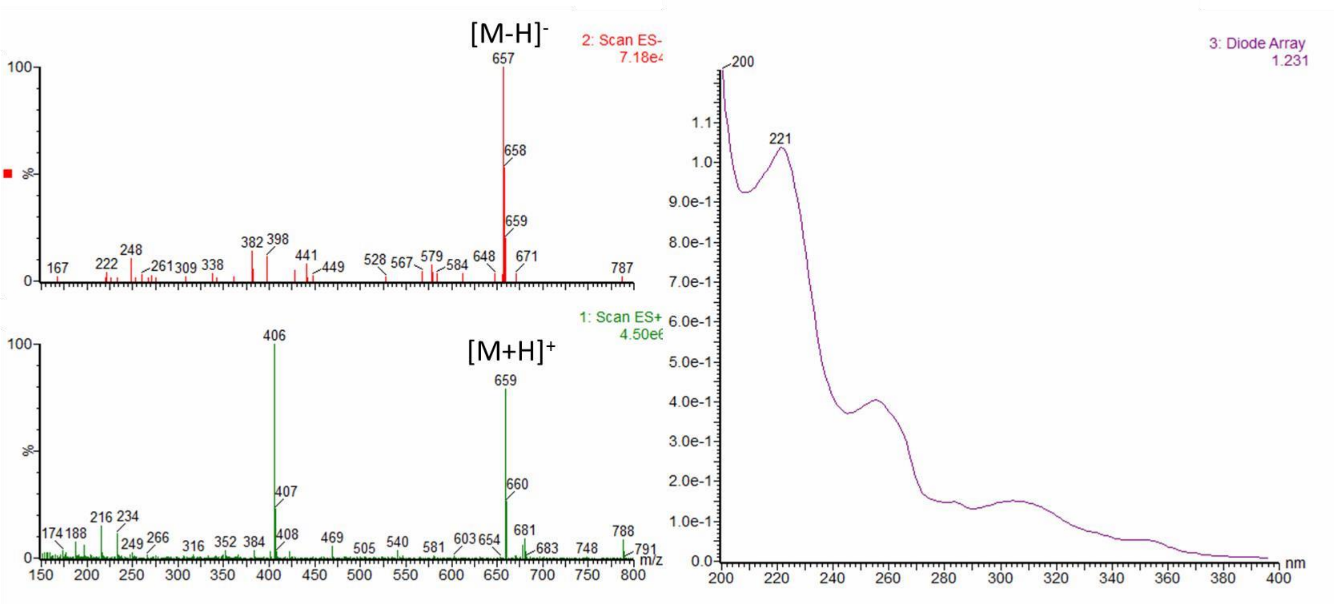

Figure S16: LCMS analysis of cycloaspeptide G **7** (molecular weight: 657.77) showing ES- and ES+ mass data and the UV absorbance.

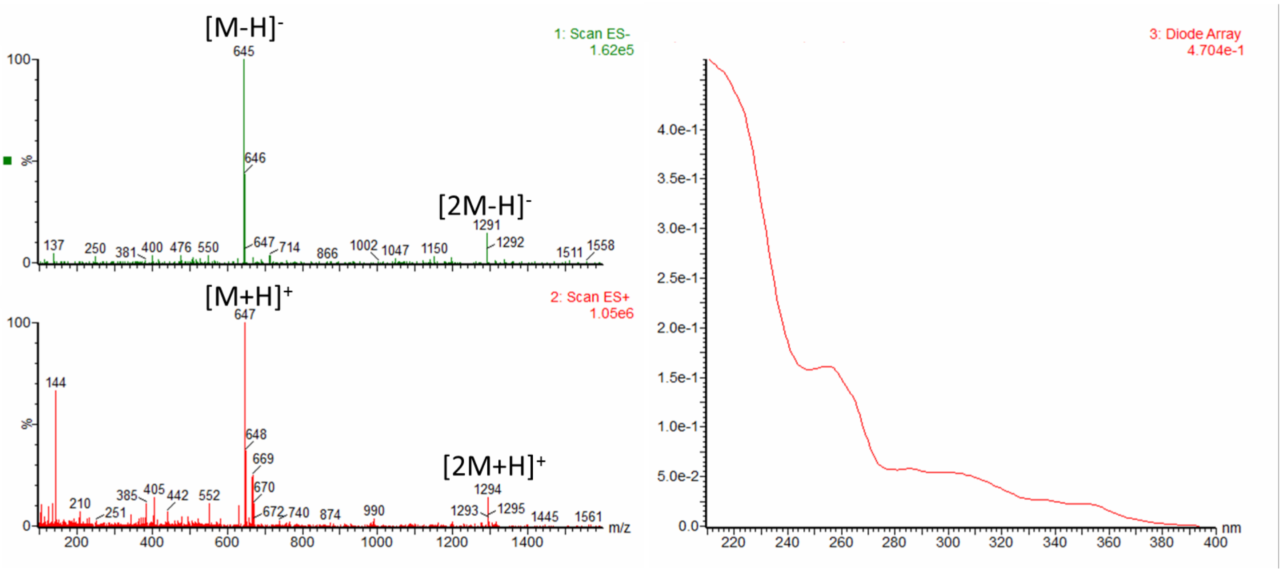

Figure S17: LCMS analysis of 4F-cycloaspeptide B **21** (molecular weight: 645.73) showing ES- and ES+ mass data and the UV absorbance.

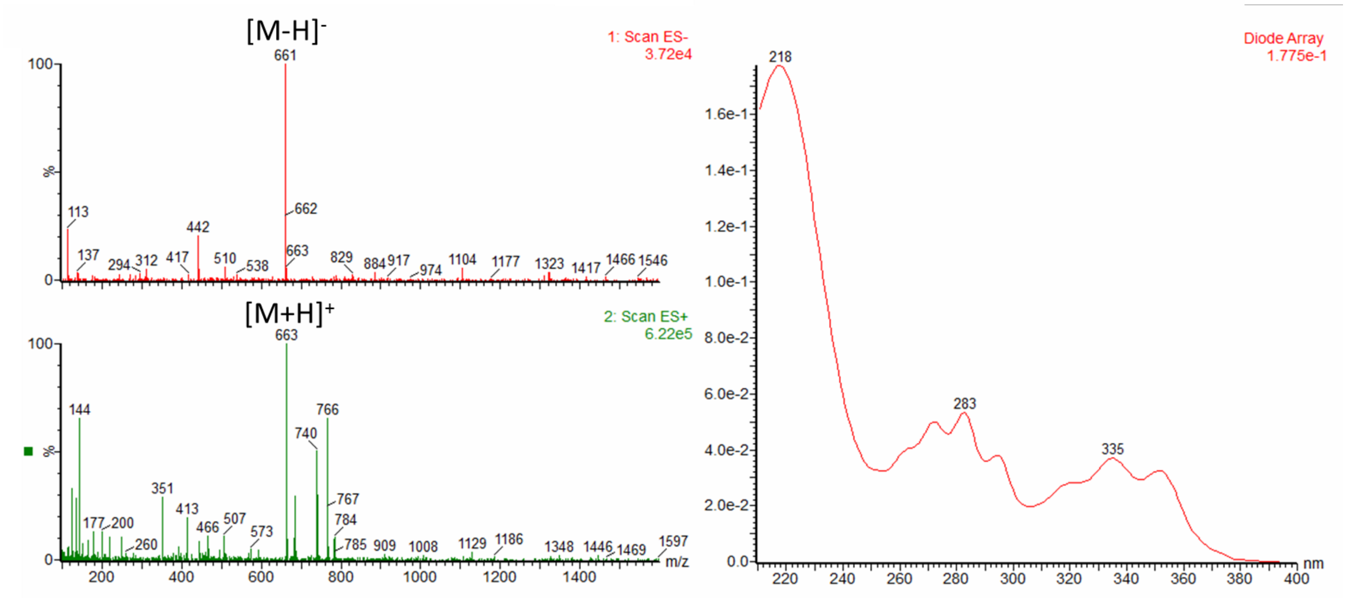

Figure S18: LCMS analysis of 4F-cycloaspeptide E **22** (molecular weight: 661.75) showing ES- and ES+ mass data and the UV absorbance.

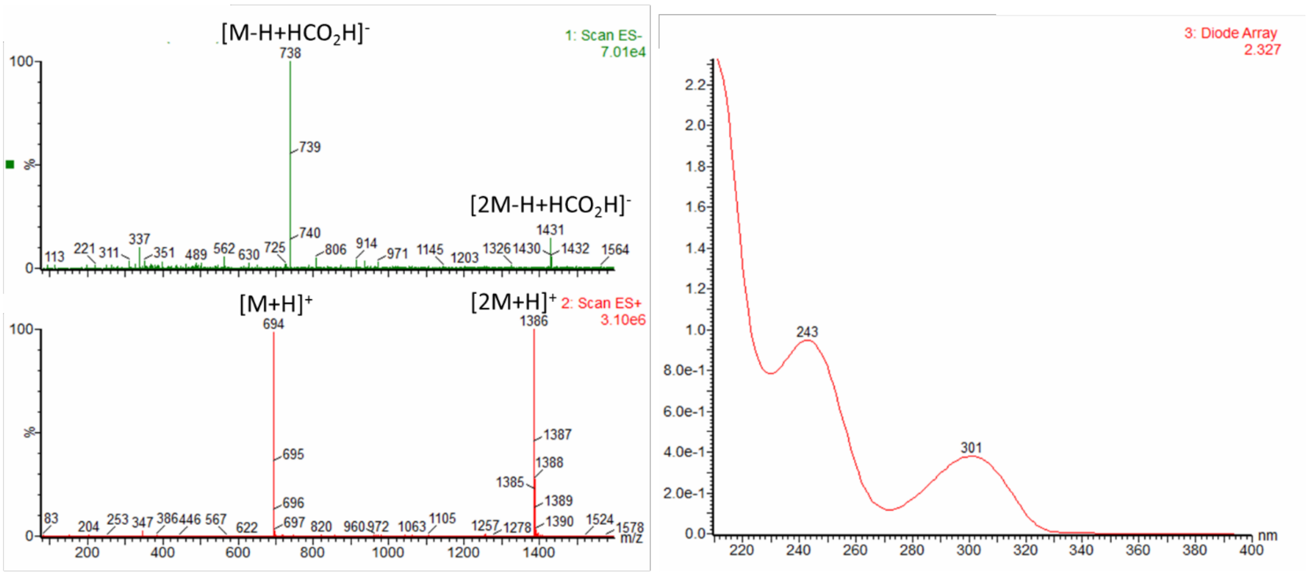

Figure S19: LCMS analysis of ditryptophenaline **23** (molecular weight: 692.82) showing ES- and ES+ mass data and the UV absorbance.

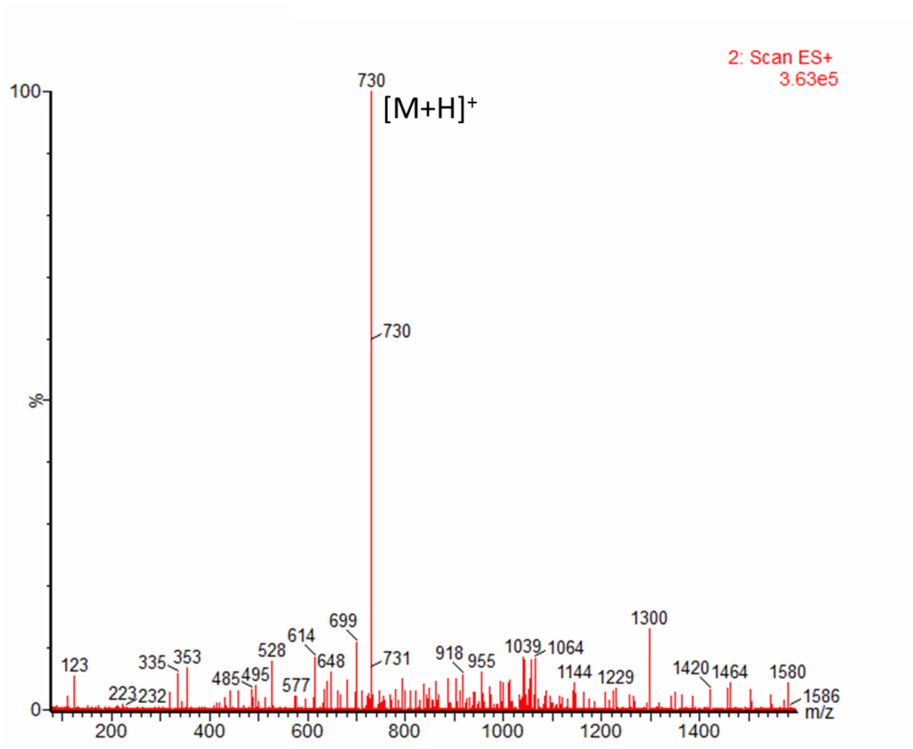

Figure S20: LCMS analysis of 4-fluoro-ditryptophenaline **24** (molecular weight: 729.3) showing the ES+ mass data. There wasn't sufficient material within the crude extract analysed to obtain clear ES- or UV data for this compound using the Waters system. High-resolution masses were obtained using the orbitrap to support the identification of this compound (see next section).

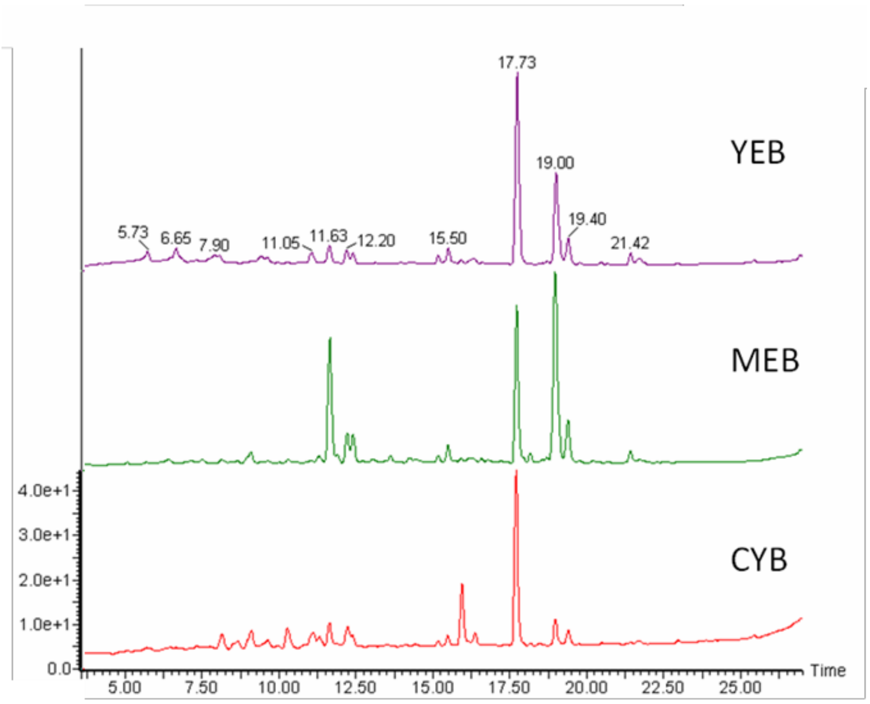

Figure S21: HPLC (DAD, 210–800 nm) chromatograms comparing *P. soppii* crude extracts grown in yeast extract broth (YEB), malt extract broth (MEB) or Czapek yeast broth (CYB) for 13 days at 25 ºC with 200 rpm shaking. Cycloaspeptide A **1** can be clearly seen in all cultures at 17.7 minutes.

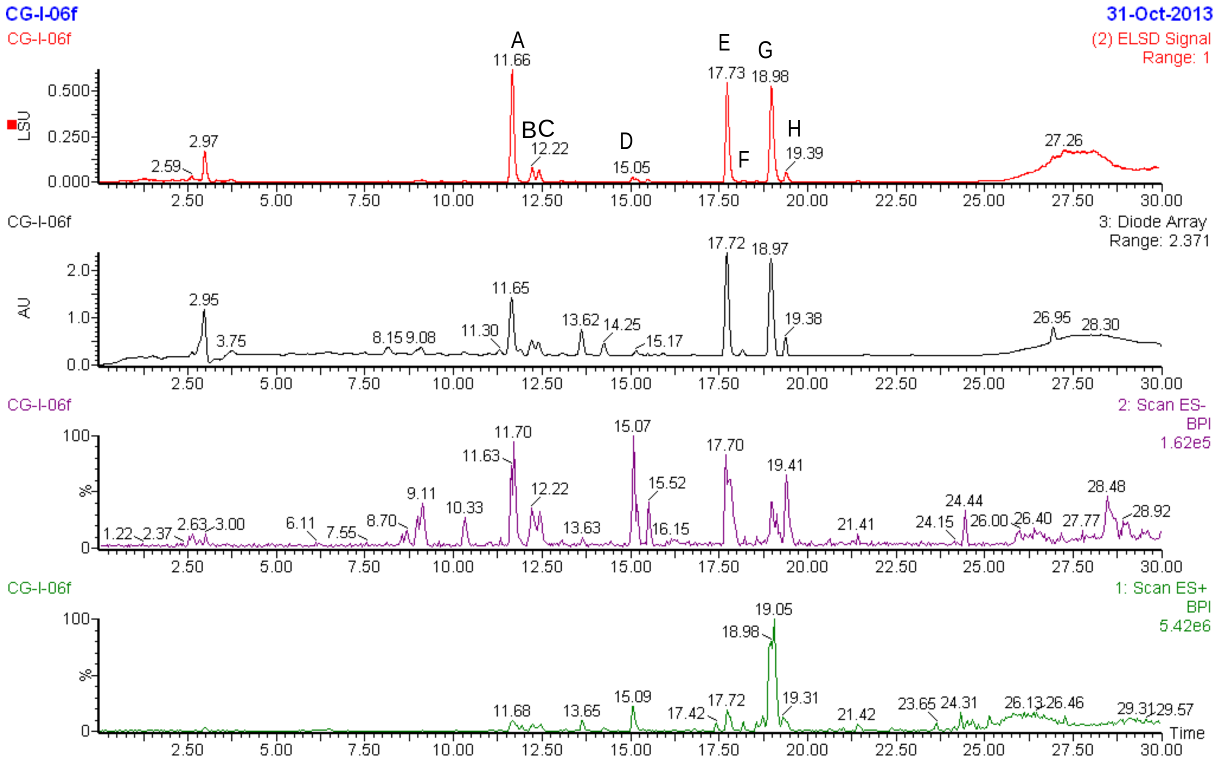

Figure S22: HPLC analysis of the crude extract from *P. soppii* grown on MEB. From the top; ELSD, ES-, ES+ and UV spectra. The masses of peaks A and B correspond to pseurotin A. Peak E is cycloaspeptide A **1**. The mass of peak F corresponds to benzomalvin A. The mass of peak G corresponds to fumagillin. The accurate masses of these peaks have been shown to correspond to the molecular formulae of these compounds (see Figure S26 and Table S6).

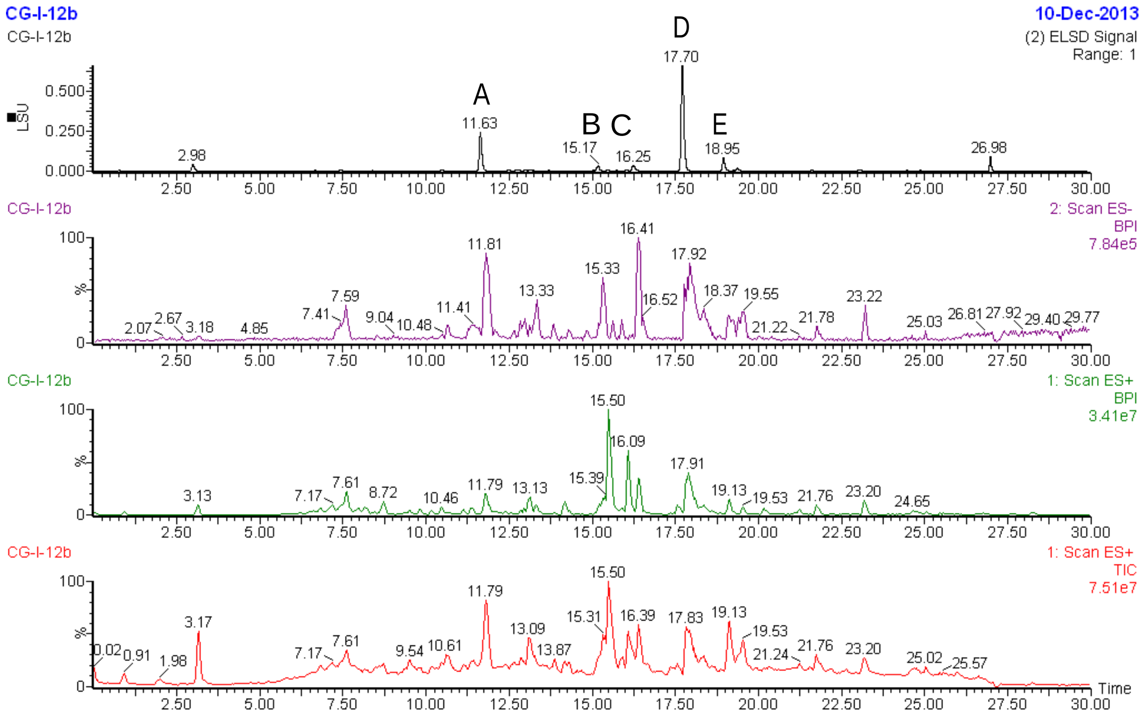

Figure S23: HPLC analysis of the crude extract for *P. jamesonlandense* grown in MEB for 14 days. ELSD, ES-, ES+ and UV (from the top). Peak A is pseurotin A. The mass of peak B corresponds to cycloaspeptide G **7**. Peak D is cycloaspeptide A **1**. The mass of peak C corresponds to cycloaspeptide D **4**. The mass of peak E corresponds to fumagillin. The accurate masses of these peaks have been shown to correspond to the molecular formulae of these compounds (see Figure S27 and Table S7).

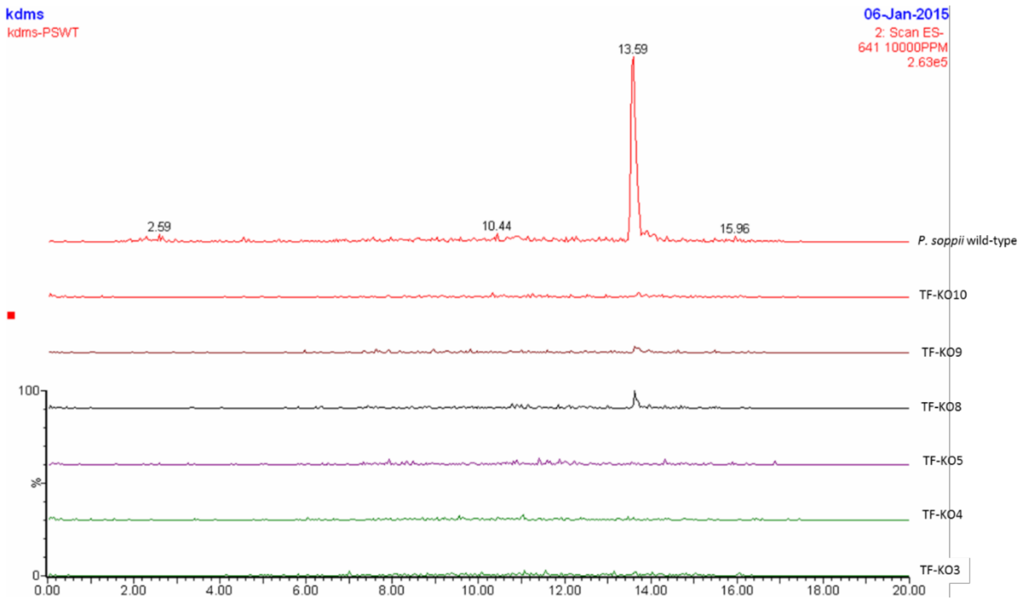

Figure S24: An extracted ion chromatogram for the mass of cycloaspeptide A **1** ([M-H]$^-$: 641.7), comparing *P. soppii* wild-type (top trace) and six transcription factor knock-out lines (Δ*PscyD*) grown on MEA. A reduction or total loss of cycloaspeptide production is seen in all transformants.

High-Resolution Accurate Mass data

Various crude extracts were analysed using an Orbitrap Elite system (Thermo Scientific) to obtain high-resolution mass-spec data and the resulting predicted formulae.

Pencillium species

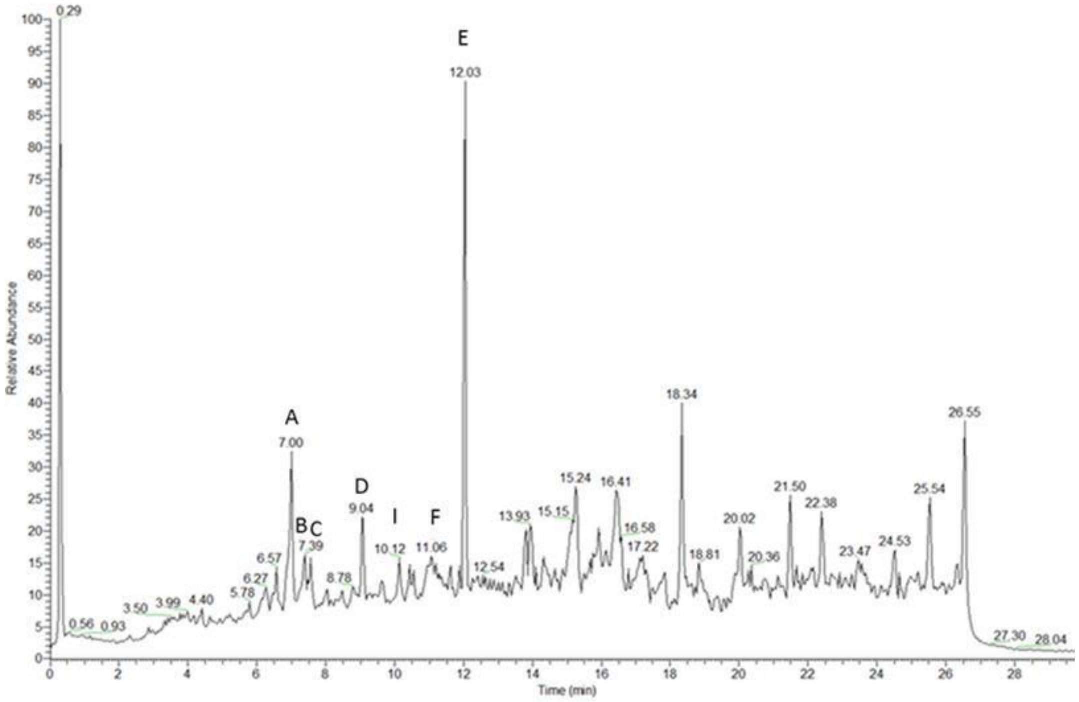

Figure S25: Orbitrap mass spectrum of *P. soppii* crude extract, grown in MEB for 2 weeks at 25°C with 200 rpm shaking. The MS data for this spectrum is shown in Table S6.

Table S6: Summary of the main peaks detected by the Waters LC-MS system and UPLC-Orbitrap Mass Analyser from a *P. soppii* MEB crude extract (2 weeks at 25 ⁰C with 200 rpm shaking)

***P. soppii* extract**									
**HPLC-MS/UV/ELSD**						**UPLC-Orbitrap**			
**peak**	**RT (min)**	**ES+**	**ES-**	**UV (nm)**	**ELSD**	**RT (min)**	**ES+**	**Formula**	**Suggested Compound**
**A**	11.7	432	430	258, 385	✓✓✓	7.0	454.1470	$C_{20}H_{19}O_6N_7$	pseurotin A
**B**	12.2	432	430	258, 385	✓	7.4	454.1470	$C_{20}H_{19}O_6N_7$	pseurotin A
**C**	12.5	316	314	258 290	✓				-
**D**	15.1	405	381	-	-				-
**E**	17.7	642	640	258, 307	✓✓✓	12.0	642.3288	$C_{36}H_{43}O_6N_5$	cycloaspeptide A
**F**	17.9	382, 404	-	300	✓	11.1	382.1503	$C_{37}H_{39}O_3N_9$	benzomalvin A
**G**	19.0	458, 481	-	300	✓✓✓				fumagillin
**H**	19.4	458, 481	-	300	✓				
**I**						10.1	658.3243	$C_{36}H_{43}O_7N_5$	cycloaspeptide G

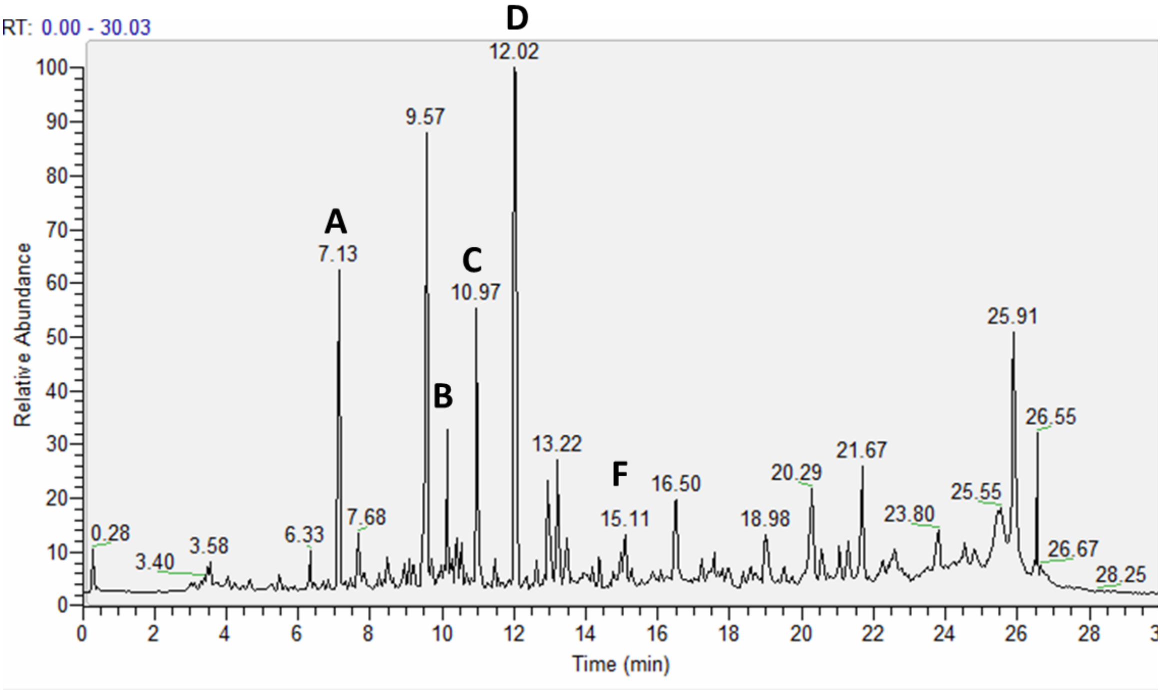

Figure S26: Orbitrap mass spectrum of *P. jamesonlandense* crude extract, grown in MEB for 2 weeks at 19 ºC with 200 rpm shaking. The MS data for this spectrum is shown in Table S7.

Table S7: Summary of the main peaks detected by the Waters LC-MS system and UPLC-Orbitrap Mass Analyser from a *P. jamesonlandense* MEB crude extract (2 weeks at 19 ºC with 200 rpm shaking)

***P. jamesonlandense* extract**									
	**HPLC-MS/UV/ELSD**					**UPLC-Orbitrap**			
**peak**	**RT (min)**	**ES+**	**ES-**	**UV (nm)**	**ELSD**	**RT (min)**	**ES+**	**Formula**	**Suggested Compound**
**A**	11.6	432	430	258, 385	✓✓✓	7.1	454.1470	$C_{20}H_{19}O_6N_7$	pseurotin A
**B**	15.2	658	656	255, 290	✓	10.14	658.3243	$C_{36}H_{43}O_7N_5$	cycloaspeptide G
**C**	16.3	628	626	255, 300	✓	10.97	628.3131	$C_{35}H_{41}O_6N_{10}$	cycloaspeptide D
**D**	17.7	642	640	258, 307	✓✓✓	12.02	642.3288	$C_{36}H_{43}O_6N_5$	cycloaspeptide A
**E**	18.9	460	458	337, 352	✓				fumagillin
**F**						15.1	626.3335	$C_{36}H_{43}O_5N_5$	cycloaspeptide E

A. flavus

Crude extracts from various *Aspergillus flavus* cultures were analysed using the orbitrap system to obtain high resolution mass data. A compound with the exact mass of 693.31843 was detected in the wild-type cultures and the Δ*dtpB* cultures fed with *N*-methylated phenylalanine, but absent from the cultures fed with 4-fluoro-phenylalanine. A compound with the exact mass of 729.29999 was uniquely detected in all three cultures fed with 4-fluoro-phenylalanine. These two accurate masses correspond to the chemical formulae $C_{42}H_{41}N_6O_4$ and $C_{42}H_{39}F_2N_6O_4$ respectively, which are the formulae for the protonated molecular ions ($[M+H]^+$) of ditryptophenaline and 4F-ditryptophenaline.

Isolated compounds

General Experimental

NMR experiments were conducted on the following spectrometers: Varian 400-MR Varian (^{1}H NMR at 400 MHz and ^{13}C NMR at 100 MHz), Varian VNMR S500 spectrometer, (^{1}H NMR at 500 MHz, ^{19}F NMR at 470 MHz and ^{13}C NMR at 125 MHz) and Bruker Advance III HD Cryo ^{13}C-probe, (^{1}H NMR at 500 MHz and ^{13}C NMR at 125 MHz). Chemical shifts were recorded in parts per million (ppm referenced to the appropriate residual solvent peak) and coupling constant (J) in Hz, reported to the closest 0.5 Hz. Multiplicity is described by the following abbreviations: s, singlet; d, doublet; t, triplet; q, quartet; m, multiplets.

The mass analyser detectors used were electrospray ionization (ESI) mass spectra, recorded on a MicrO-ToF II Bruker Daltonics mass spectrometer and Orbitrap Elite Thermo Scientific mass spectrometer.

Cycloaspeptide A 1

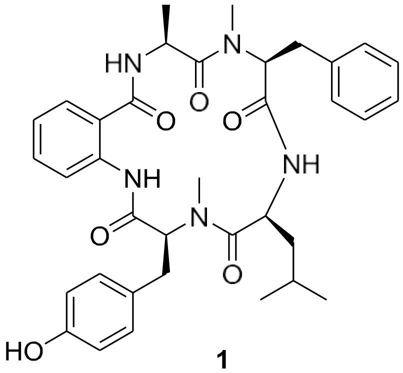

1

Cycloaspeptide A **1** was purified as white solid (1.3 mg); UV λ_{max} nm 257, 300; ^{1}H NMR (CDCl$_3$, 500 MHz) Table S8; ^{13}C NMR (CDCl$_3$, 125 MHz) δ_C 173.70, 169.90, 169.69, 168.21, 168.15, 154.58, 141.75, 137.77, 134.44, 130.80, 130.48, 129.52, 129.31, 127.37, 127.01, 122.42, 121.09, 115.78, 115.25, 77.41, 77.36, 77.16, 76.91, 70.04, 63.46, 48.89, 44.13, 41.40, 34.17, 32.23, 30.22, 29.86, 25.00, 23.43, 22.12, 16.56; HRMS m/z calculated for $C_{36}H_{43}N_5O_6$ 641.32134; observed m/z 642.32999.

Table S8: ^{1}H NMR data for purified cycloaspeptide A **1** compared to the published data[17]

		^{1}H NMR of the Purified Compound		^{1}H NMR Literature	
**Residue**	**Position**	***δ* / ppm**	***J* / Hz**	***δ* / ppm**	***J* / Hz**
**Ala**	NH	6.59 (1H)	d 7.0	6.96 (1H)	d 6.96
	$CH_α$	4.41 (1H)	m	4.41 (1H)	m
	$CH_β$	0.42 (3H)	d 6.5	0.40 (3H)	d 6.6
**Phe**	NCH_3	2.88 (3H)	s	2.87 (3H)	s
	$CH_α$	5.20 (1H)	dd 3.0, 12.0	5.21 (1H)	dd 3.4, 11.7
	$CH_β$	2.95 (1H)	dd 12.0, 14.5	2.96 (1H)	dd 12.2, 13
		3.48 (1H)	dd 4.4, 14.5	3.48 (1H)	dd 4, 14.2
	C2 C6	7.17 (2H)	m	7.17 (2H)	d 8.2
	C3 C5	7.32 (2H)	m	7.33 (2H)	m
	C4	7.26 (1H)	m	7.26 (1H)	m
**Leu**	NH	7.13 (1H)	d 8.5	7.30 (1H)	d 7.8
	$CH_α$	4.72 (1H)	m	4.72 (1H)	ddd 8, 8, 8
	$CH_β$	1.36 (1H)	m	1.36 (1H)	ddd 5.5, 8, 13
		1.86 (1H)	ddd 5.5, 8.0, 14.0	1.86 (1H)	ddd 5.5, 8.5, 13
	$CH_γ$	1.67 (1H)	m	1.67 (1H)	m
	CH_3	1.02 (3H)	d 6.5	0.98 (3H)	d 6.4
**Tyr**	NCH_3	2.73 (3H)	S	2.69 (3H)	s
	$CH_α$	3.80 (1H)	dd 4.0, 14.0	3.80 (1H)	dd 4, 11.3
	$CH_β$	3.37 (1H)	dd 11.0, 14.0	3.35 (1H0	dd 11.3, 14.2
		3.49 (1H)	dd 4.0, 14.0	3.48 (1H)	dd 4, 14.2
	C2 C6	7.04 (2H)	d 8.0	6.98 (2H)	d 8.6
	C3 C5	6.79 (2H)	d 8.0	6.79 (2H)	d 8.6
**ABA**	NH	12.01 (1H)	s	12 (1H)	s
	C3	7.42 (1H)	d 8.5	7.44 (1H)	d 7.8
	C4	7.00 (1H)	t 8.0	6.96 (1H)	t 7.8
	C5	7.49 (1H)	t 8.5	7.46 (1H)	t 8.6
	C6	8.92 (1H)	d 8.0	8.83 (1H)	d 8.6

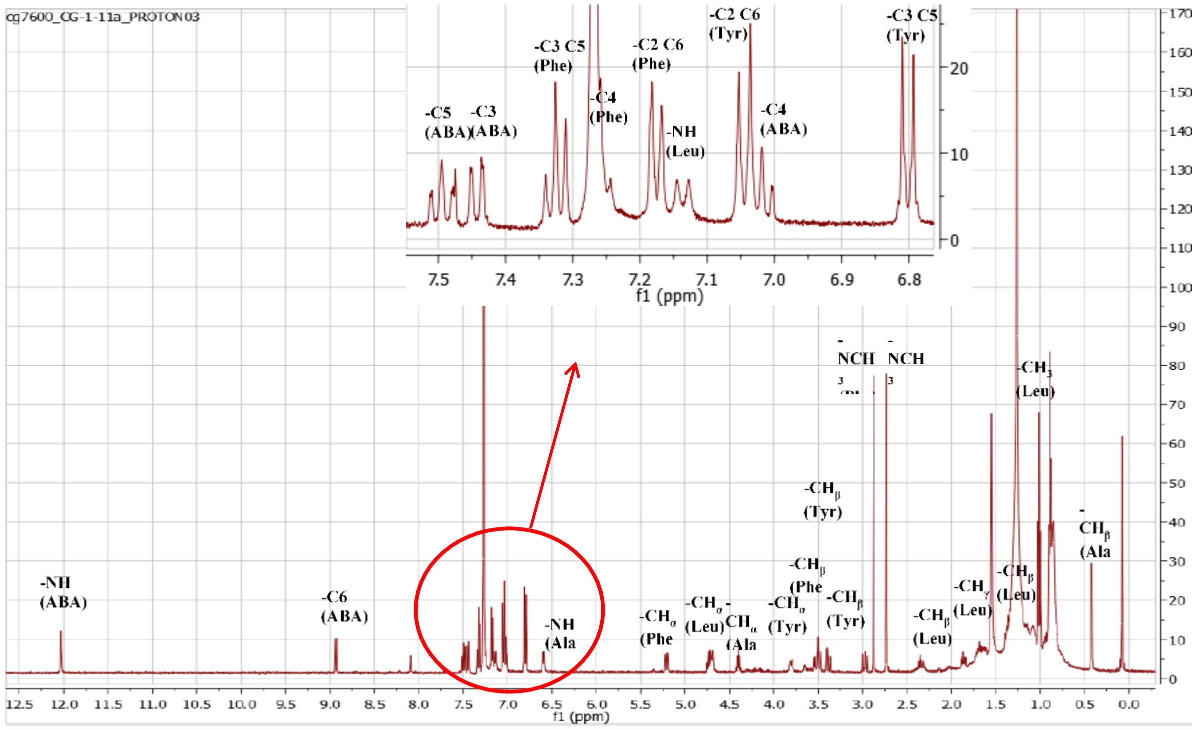

Figure S27: Proton NMR spectra for cycloaspeptide A **1**, purified from *P. soppii*

Cycloaspeptide E 5

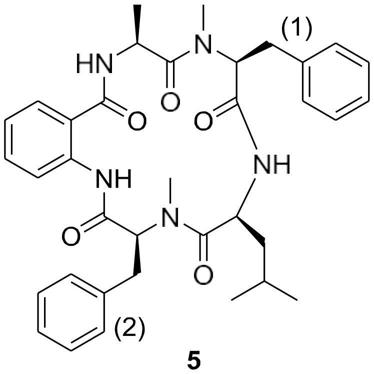

5

P. soppii ΔpscyA strain was grown in CYB (10 ×100 mL flasks), fed with *N*-methylphenylalanine (100 mg) and extracted after 14 days. The crude extract was purified using preparative HPLC using *Method 1* to obtain cycloaspeptide E **5** as a white solid (1.3 mg); λ_{max} / nm 257, 300; δ_H ($CDCl_3$, 500 MHz) Error! Reference source ot found.; δ_C ($CDCl_3$, 125 MHz) **Table S9**; *m/z* (ESI) 626.5862 $[M+H]^+$ ($C_{36}H_{44}N_5O_5$ requires 626.3264). Data in accordance with the literature.[180]

Table S9: NMR data for cycloaspeptide E **5**, isolated from *P. soppii* strain NM-KO4 (Δ*PscyF*) fed with *N*-methyl-L-phenylalanine, compared to the literature data ([a]500 MHz and [b]126 MHz, $CDCl_3$).[18]

		Isolated		Literature	
**Residue**	**Position**	**δ_C[b] / ppm**	**δ_H[a] / ppm (*J* in Hz)**	**δ_C / ppm**	**δ_H/ ppm (*J* in Hz)**
**Ala**	NH		6.77 (1H, d, *J* 7.0)		6.60 (1H, d, *J* = 7.0 Hz)
	C_α	44.1	4.41 (1H, m)	44.4	4.41 (1H, dq, *J* = 7.0, 6.6 Hz)
	C_β	16.5	0.41 (3H, d, *J* 7.0)	16.8	0.43 (3H, d, *J* = 6.6 Hz)
	CO	173.9	-	173.9	
	N-Me	30.3	2.87 (3H, s)	30.4	2.89 (3H, s)
	C_α	63.5	5.20 (1H, dd, *J* 12.0, 3.0)	63.7	5.21 (1H, dd, *J* = 11.8, 3.0 Hz)
	C_β	34.2	3.49 (1H, dd, *J* 14.0, 3.0)	34.4	3.49 (1H, dd, *J* = 14.3, 2.6 Hz)
			2.96 (1H, dd, *J* 15.0, 12.0)		3.0 (1H, dd, *J* = 14.9, 12.3 Hz)
**Phe(1)**	C1	138.8	-	139.1	
	C2-5	129	7.36-7.28 (4H, m)	129	7.36-7.28 (4H, m)
	C6	127	7.35 (1H, m)	127	7.35 (1H, m)
	CO	168.1	-	168.3	
	NH		7.14 (1H, m)		7.14 (1H, d, *J* = 8.7 Hz)
	C_α	48.9	4.72 (1H, ddd, *J* 9.0, 9.0, 6.0)	48.8	4.72 (1H, ddd, *J* =8.5, 8.5, 5.6 Hz)
	C_β	41.4	1.86 (1H, m)	41.7	1.88 (1H, m)
			1.35 (1H, m)		1.38 (1H, m)
**Leu**	C_γ	25.0	1.69 (1H, m)	25.2	1.71 (1H, m)
	C_δ	23.4	1.01 (3H, d, *J* 6.0)	23.7	1.02 (3H, d, *J* = 6.7 Hz)
	C_ε	22.1	1.00 (3H, d, *J* 6.0)	22.4	1.01 (3H, d, *J* = 6.7 Hz)
	CO	170.0	-	170.2	
	N-Me	39.2	2.68 (3H, s)	39.5	2.70 (3H, s)
	C_α	70.0	3.86 (1H, dd, *J* 11.0, 4.0)	70.0	3.87 (1H, dd, *J* =10.8, 3.7 Hz)
	C_β	33.2	3.59 (1H, dd, *J* 14.0, 4.0)	33.4	3.62 (1H, dd, *J* =13.8, 4.1 Hz)
			3.47 (1H, dd, *J* 14.0, 11.0)		3.47 (1H, dd, *J* =14.3, 11.3 Hz)
**Phe(2)**	C1	137.7	-	138.1	
	C2-5	129	7.36-7.28 (4H, m)	129	7.36-7.28 (4H, m)

	C6	127	7.35 (1H, m)	127	7.35 (1H, m)
	CO	170.0	-	171.0	
	NH		12.03 (1H, s)		12.03 (1H, s)
	C1	115.3	-	115.4	
**ABA**	C2	141.7	-	142	
	C3	121.1	8.92 (1H, d, *J* 9.0)	121.4	8.95 (1H, d, *J* =8.7 Hz)
	C4	134.5	7.51 (1H, m)	134.7	7.51 (1H, dd, *J* =8.7, 8.2 Hz)
	C5	122.5	7.02 (1H, dd, *J* 8.0, 8.0)	122.7	7.02 (1H, dd, *J* =8.2, 8.2 Hz)
	C6	127.0	7.45 (1H, d, *J* 8.0)	127.2	7.45 (1H, d, *J* =8.2 Hz)
	CO	169.7		170.0	

4F-cycloaspeptide E 22

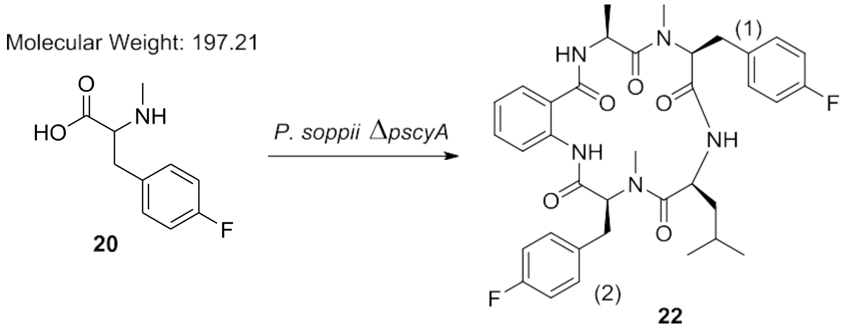

P. soppii ΔpscyA strain was grown in CYB (5 ×100 mL), fed with *m*-fluoro-*N*-methylphenylalanine **20** (5 mM final concentration) and extracted after 14 days. The crude extract was purified by preparative HPLC using *Method 1* to obtain 4F-cycloaspeptide E **22** as a white solid (1.3 mg); λ_{max} / nm 257, 300; δ_H ($CDCl_3$, 500 MHz) **Table S10**; δ_C ($CDCl_3$, 125 MHz) **Table S10**; δ_F ($CDCl_3$, 470 MHz) **Table S10**; *m/z* (ESI) 684.2948 $[M+Na]^+$ ($C_{36}H_{41}F_2N_5NaO_5$ requires 684.2968).

Table S10: NMR data for 4F-cycloaspeptide E **22** ([a]500 MHz, [b]126 MHz and [c]470 MHz, $CDCl_3$).

**4F-cycloaspeptide 12**				
**Residue**	**Position**	**δ_C[b]/ppm (*J* / Hz)**	**δ_H[a] ppm (*J* / Hz)**	**δ_F[c] ppm (*J* / Hz)**
**Ala**	NH	-		-
	C_α	44.0	4.43 (1H, m)	-
	C_β	16.7	0.53 (3H, d, *J* 7.0)	-
	CO	173.9	-	-
	N-Me	30.3	2.86 (3H, s)	-
	C_α	63.4	5.17 (1H, dd, *J* 12.0, 3.0)	-
	C_β	3.4	3.47 (1H, dd, *J* 15.0, 3.0)	-
			2.94 (1H, dd, *J* 15.0, 12.0)	-
**F-Phe(1)**	C1	132.4 (d, *J* = 3 Hz)	-	-
	C2-6	130.8 (d, *J* = 8 Hz)	7.14 (2H, m)	-
	C3-5	115.8 (d, *J* = 21 Hz)	7.02 (2H, m)	-
	C4-F	162.0 (d, *J* = 245 Hz)	-	-
	F1	-	-	-115.96 (tt, *J* = 5, 8)

	CO	168.0	-	-
	NH	-	7.21 (1H, m)	-
	C_{α}	48.9	4.72 (1H, ddd, *J* 9.0, 9.0, 6.0)	-
	C_{β}	41.4	1.84 (1H, m)	-
			1.35 (1H, m)	-
**Leu**	C_{γ}	25.0	1.65 (1H, m)	-
	C_{δ}	23.4	1.00 (3H, d, *J* 3.0)	-
	C_{ε}	22.1	0.99 (3H, d, *J* 3.0)	-
	CO	170.0	-	-
	N-Me	39.3	2.74 (3H, s)	-
	C_{α}	69.9	3.82 (1H, dd, *J* 11.0, 4.0)	-
	C_{β}	32.4	3.57 (1H, dd, *J* 14.0, 4.0)	-
			3.43 (1H, m)	-
**F-Phe(2)**	C1	134.5 (d, *J* = 3 Hz)	-	-
	C2-6	131.0 (d, *J* = 8 Hz)	7.14 (2H, m)	-
	C3-5	116.2 (d, *J* = 21 Hz)	7.02 (2H, m)	-
	C4-F	162.2 (d, *J* = 246 Hz)	-	-
	F2	-	-	-115.2 (tt, *J* = 5, 9)
	CO	169.8	-	-
	NH	-	12.01 (1H, s)	-
	C1	115.4	-	-
**ABA**	C2	141.6	-	-
	C3	121.0	8.90 (1H, d, *J* 9.0)	-
	C4	134.4	7.42 (1H, m)	-
	C5	122.5	7.02 (1H, dd, *J* 8.0, 8.0)	-
	C6	127.2	7.45 (1H, d, *J* 8.0)	-
	CO	169.8	-	-

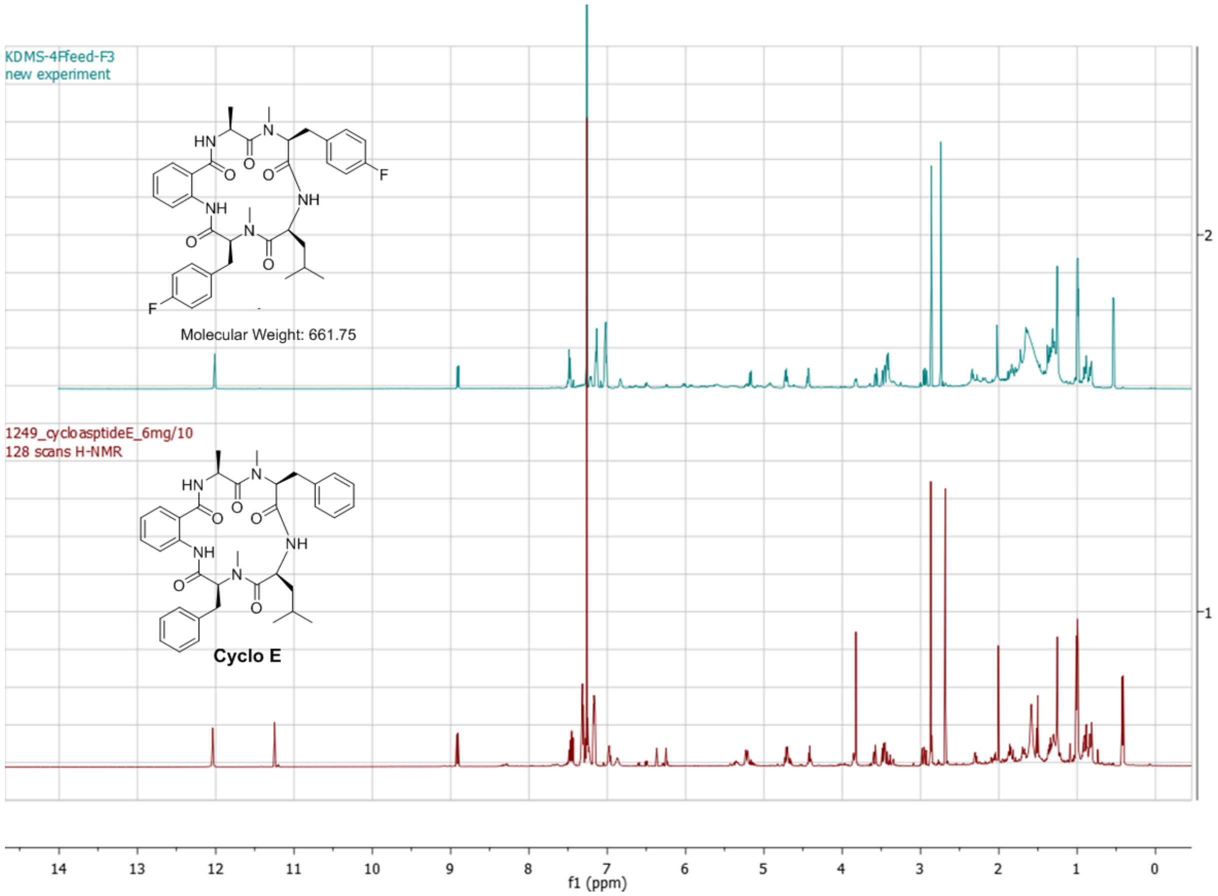

Figure S28: Proton NMR spectra for cycloaspeptide E **5**, purified from *P. soppii* strain NM-KO4 (Δ*PscyF*) fed with *N*-methyl-L-phenylalanine **8** (bottom trace), compared to 4F-cycloaspeptide E **22**, purified from *P. soppii* strain NM-KO4 (Δ*PscyF*) fed with synthesised 4F-*N*-methyl-phenylalanine **S2** (top trace).

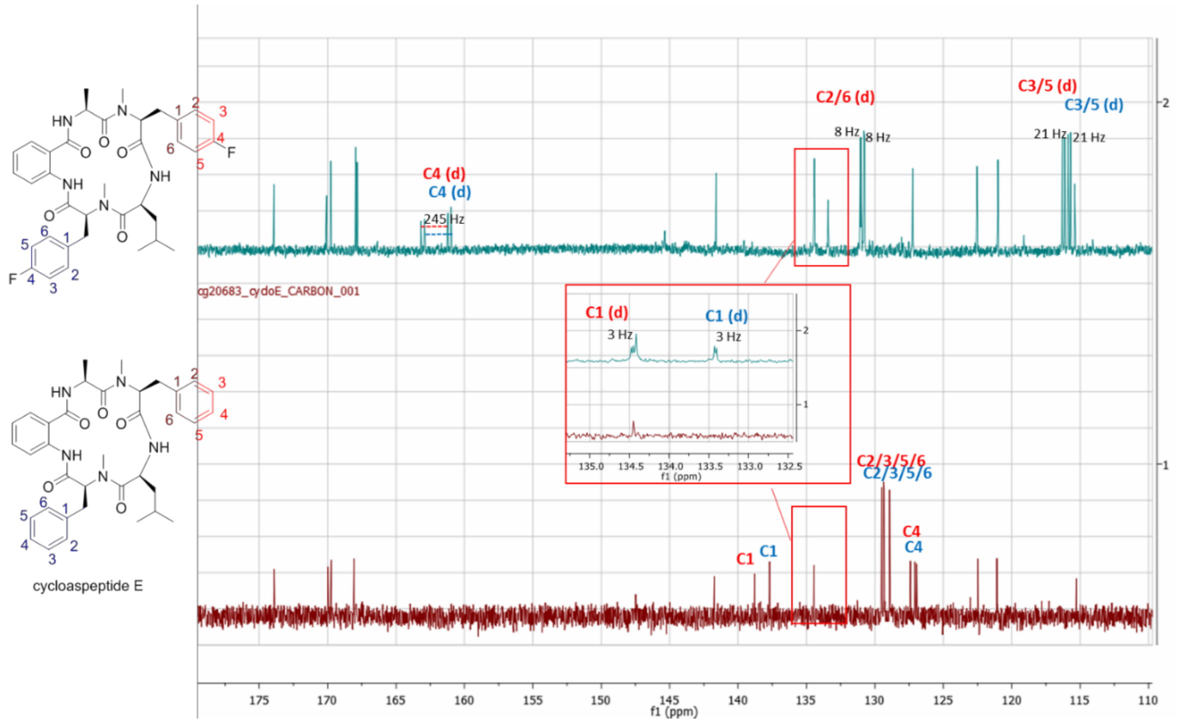

Figure S29: Carbon NMR spectra for cycloaspeptide E **5**, purified from *P. soppii* strain NM-KO4 (Δ*PscyF*) fed with *N*-methyl-L-phenylalanine **8** (bottom trace), compared to 4F-cycloaspeptide E **22**, purified from *P. soppii* strain NM-KO4 (Δ*PscyF*) fed with synthesised 4F-*N*-methyl-phenylalanine **S2** (top trace).

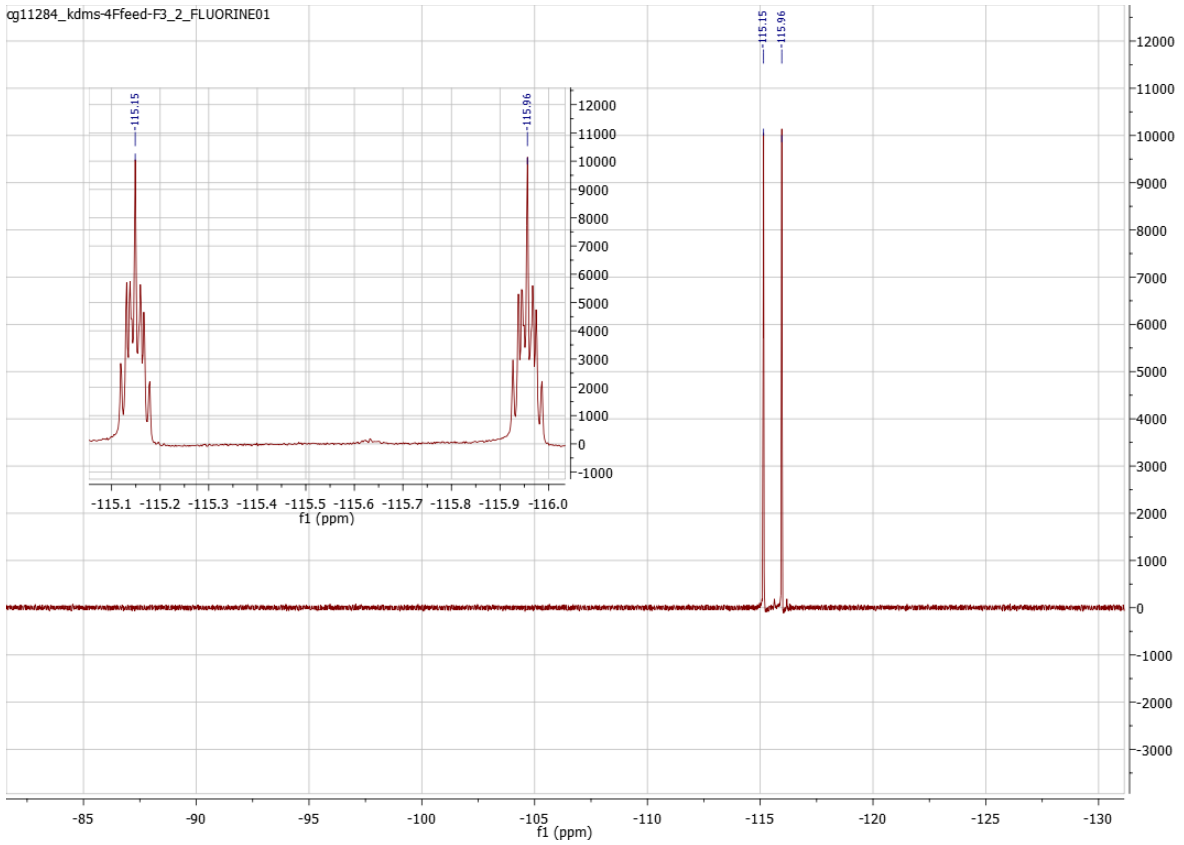

Figure S30: Fluorine NMR spectra for 4F-cycloaspeptide E **22**, purified from *P. soppii* strain NM-KO4 (Δ*PscyF*) fed with synthesised 4F-*N*-methyl-phenylalanine **S2**.

Pseurotin A 25

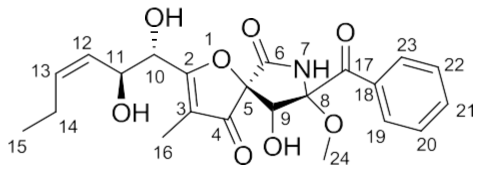

25

Pseurotin A **25** was purified as a white solid (4.3 mg); UV λ_{max} nm 307; ^{1}H NMR ($CDCl_3$, 500 MHz) (Table S11). HRMS m/z calculated for $C_{22}H_{25}NO_8$ 431.1580; observed m/z 432.1658.

Table S11: ^{1}H-NMR data of the purified pseurotin A **25** compared to the literature data after a D_2O wash (500 MHz in $CDCl_3$).[19]

	^{1}H NMR purified compound		^{1}H NMR Literature*	
**Position**	***δ* / ppm**	***J* / Hz)**	***δ* / ppm**	***J* / Hz**
**9**	4.69 (1H)	s	4.70 (1H)	s
**10**	4.58 (1H)	d (4.5)	4.59 (1H)	d (4.4)
**11**	4.74 (1H)	dd (4.5, 9.0)	4.75 (1H)	dd (4.4, 9.0)
**12**	5.38 (1H)	dd (9.0, 12.0)	5.28 (1H)	dd (9.0, 11.0)
**13**	5.68 (1H)	dt (12.0, 8.0)	5.60 (1H)	dt (11.0, 7.6)
**14**	2.08-2.25 (2H)	m	2.05-2.24 (2H)	m
**15**	1.02 (3H)	t (7.5)	0.99 (3H)	t (7.6)
**16**	1.72 (3H)	s	1.68 (3H)	s
**19/23**	8.32 (2H)	d (7.5)	8.32 (2H)	d (7.3)
**20/22**	7.50 (2H)	t (7.5)	7.49 (2H)	t (7.3)
**21**	7.66 (1H)	t (7.5)	7.65 (1H)	t (7.3)
**8-OMe**	3.39 (3H)	s	3.44 (3H)	s

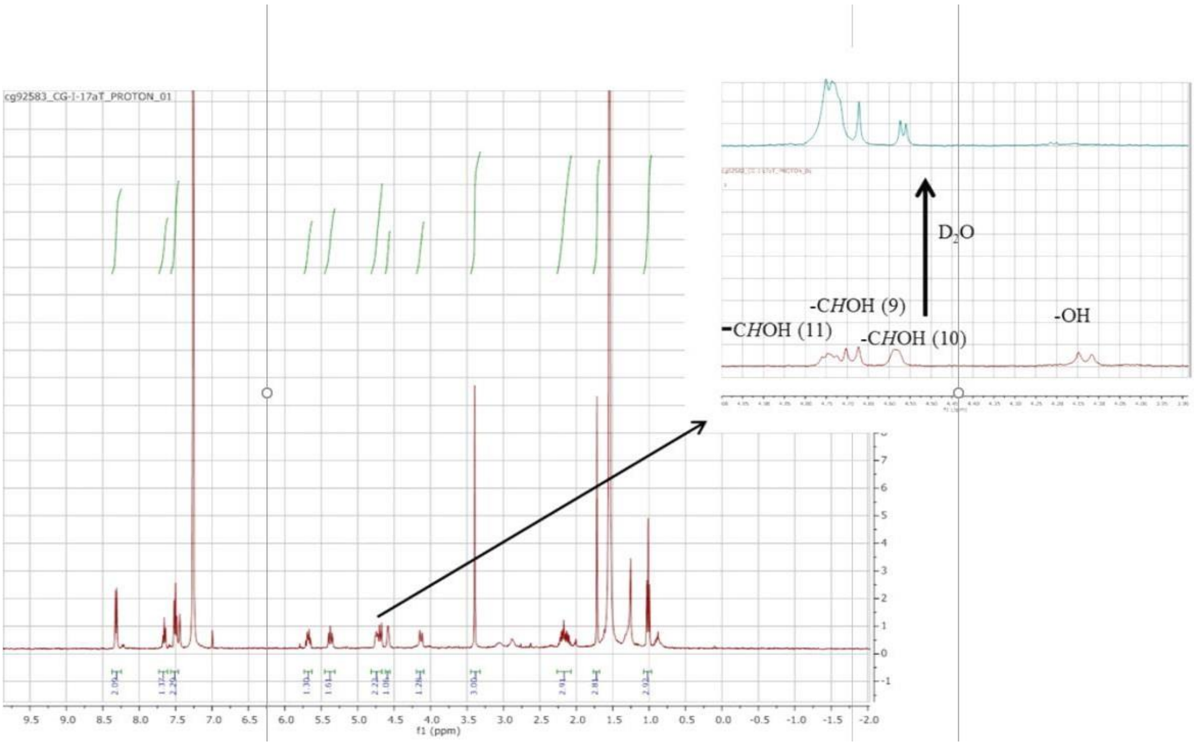

Figure S31: ^{1}H NMR spectra for pseurotin A **25**, purified from *P. soppii*.

Synthesis of Amino Acids

General Experimental

All commercially available compounds were used without further purification, with the exception of diisopropylamine (distilled from CaH_2). All moisture or air sensitive reactions were carried out in flame-dried glassware under a positive pressure of nitrogen using syringe/septa techniques. Anhydrous solvents were obtained by passing through a modified Grubbs system of alumina columns, manufactured by Anhydrous Engineering.

Reactions were routinely monitored by thin layer chromatography on aluminium backed silica plates (Merck DC Alufolien Keiselgel 60 F254). Detection was under UV light source (λ_{max} 254 nm) or through staining with potassium permanganate solution (5%) with subsequent heating. Flash column chromatography was performed using silica gel (40-63 micron, obtained from Sigma Aldrich) as the stationary phase.

^{1}H, ^{19}F and ^{13}C NMR spectra were recorded using either a Jeol ECS300, Jeol ECS400 or Varian 400-MR spectrometer. The chemical shifts (δ) are reported in parts per million (ppm) and are referenced to the residual solvent peak. Coupling constants (*J*) are measured in Hertz (Hz). COSY, HSQC and HMBC were routinely used to define the peaks of ^{1}H and ^{13}C NMR spectra. ^{1}H NMR is reported in the format: chemical shift (ppm), integration, multiplicity (s=singlet, d=doublet, t =triplet, q=quartet, m=multiplet, br=broad), coupling constant, assignment.

Optical rotations were recorded using Bellingham and Stanley ADP220 polarimeter, irradiating with sodium D line (λ=589nm) and $[\alpha]_D$ values are quoted in units 10^{-1} deg cm^2 g^{-1}. Infrared spectra were recorded on a Perkin Elmer Spectrum Two spectrometer as solids or as a thin film. Significant frequencies, ν are reported in wavenumber units (cm^{-1}).

Electrospray (ESI) mass spectra were recorded on a VG Analytical Quattro mass spectrometer.

(±)-*N*-(*tert*-butoxycarbonyl)-*N*-methyl-*p*-fluorophenylalanine S2

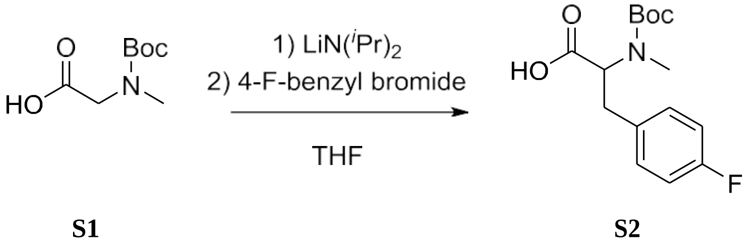

S1 **S2**

To diisopropylamine (2.5 mL, 17.4 mmol, 3.3 equiv.) in THF (10 mL) at -15 °C was added *n*-BuLi (1.45 M in hexane, 11.0 mL, 15.8 mmol, 3 equiv.) and the mixture stirred at -15 °C for 30 minutes, then allowed to warm to 0 °C. *N*-Boc sarcosine **S1** (1.01 g, 5.3 mmol, 1 equiv.) was dissolved in THF (15 mL), and added at -10 °C to the stirred LDA solution under nitrogen. After 1 h, the reaction mixture was cooled to -40 °C and 4-fluorobenzyl bromide (1.6 mL, 13.0 mmol, 2.5 equiv.) added dropwise. The reaction mixture was stirred for 3 h, then quenched with saturated aqueous ammonium chloride (10 mL). The reaction mixture was acidified to pH 2 with HCl (1 M), and extracted with ethyl acetate (2 × 50 mL). The extract was dried with magnesium sulfate, and concentrated *in vacuo* to yield an orange oil. The crude material was purified by column chromatography using ethyl acetate/petrol/acetic acid (10-35% ethyl acetate, 1% acetic acid) to yield acid **S2** (1.17 g, 74%) as an off white solid; δ_H(400 MHz, $CDCl_3$): 7.16 (2H, m, Ar-H), 6.99 (2H, m, Ar-H), 4.81 (0.5H, dd, *J* 11.0, 5.0, 2-H), 4.55 (0.5H, dd, *J* 11.0, 4.0, 2-H), 3.28 (1H, m, 3-H) and 3.04 (1H, m, 3-H), 2.74 (1.5H, s, N-CH_3), 2.69 (1.5H, s, N-CH_3), 1.38 (4.5H, s, ^{t}Bu) 1.33 (4.5H, s, ^{t}Bu); δ_F(283 MHz, $CDCl_3$): -116.1 (m, Ar-F), -116.3 (m, Ar-F); δ_C(101 MHz, $CDCl_3$): 175.8, 163.1, 160.6, 156.4, 155.2, 133.3, 133.0, 130.6, 130.5, 130.4, 115.6, 115.5, 115.4, 115.3, 81.0, 80.9, 61.6, 60.4, 34.6, 34.1, 32.7, 28.4, 28.3; ν_{max} (film) 2978, 1682, 1510, 1392, 1368, 1222, 1158; HRMS (ESI) calc for $C_{15}H_{20}FNNaO_4$ $[M+Na]^+$ 320.1269, found 320.1265.

(±)-*N*-(*tert*-butoxycarbonyl)-*N*-methyl-*m*-fluorophenylalanine S3

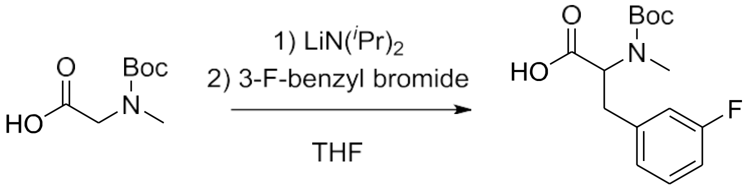

S1 **S3**

(±)-*N*-(*tert*-butoxycarbonyl)-*N*-methyl-*m*-fluorophenylalanine **S3** was prepared similarly from *N*-Boc sarcosine **S1** (500 mg, 2.64 mmol) and 3-fluorobenzyl bromide (1.3 mL, 10.6 mmol, 4 equiv.), yielding the product as a white solid (397 mg, 51%) δ_H(400 MHz, $CDCl_3$): 9.95 (1H, s, COOH), 7.26 (1H, m, Ar-H), 7.02-6.87 (3H, m, Ar-H), 4.84 (0.5H, dd, *J* 11.0, 5.0, 2-H), 4.63 (0.5H, dd, *J* 11.0, 4.0, 2-H), 3.32 (1H, m, 3-H), 3.00 (1H, m, 3-H), 2.76 (1.5H, s, N-CH_3), 2.70 (1.5H, s, N-CH_3), 1.40 (4.5H, s, ^{t}Bu) 1.35 (4.5H, s, ^{t}Bu); δ_F(283 MHz, $CDCl_3$): -113.1 (m, Ar-F), -113.3 (m, Ar-F); δ_C(101 MHz, $CDCl_3$): 176.2, 176.1, 164.2, 161.7, 156.3, 155.1, 140.0, 139.9, 139.8, 130.2, 130.2, 130.1, 130.0, 124.9, 124.7, 116.1, 115.9, 114.0, 113.8, 113.8, 113.6, 81.1, 80.9, 61.2, 60.4, 35.1, 34.6, 33.0, 32.6, 28.3, 28.3; ν_{max} (film) 2972, 1713, 1689, 1585, 1486, 1400, 1249, 1140; HRMS (ESI) calc for $C_{15}H_{20}FNNaO_4$ $[M+Na]^+$ 320.1269, found 320.1267.

(±)-*N*-(*tert*-butoxycarbonyl)-*N*-methyl-*o*-fluorophenylalanine S4

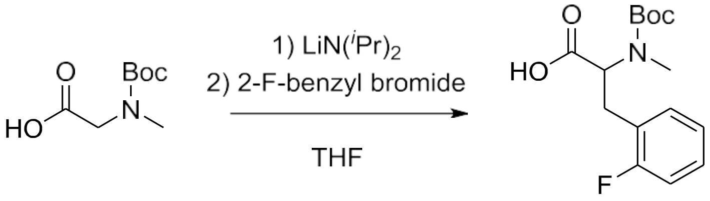

S1 **S4**

(±)-*N*-(*tert*-butoxycarbonyl)-*N*-methyl-*o*-fluorophenylalanine **S4** was prepared similarly from *N*-Boc sarcosine **S1** (500 mg, 2.64 mmol) and 2-fluorobenzyl bromide (1.3 mL, 10.6 mmol), yielding the product as a white solid (504 mg, 64%) δ_H(400 MHz, $CDCl_3$): 10.80 (1H, s, COOH), 7.25-7.13 (2H, m, Ar-H), 7.09-6.98 (2H, m, Ar-H), 4.89 (0.4 H, dd, *J* 11.0, 5.0, 2-H), 4.70 (0.6 H, dd, *J* 11.0, 4.0, 2-H), 3.34 (1H, m, 3-H), 3.24 (0.4H, m, 3-H), 3.07 (0.6H, m, 3-H), 2.76 (1.5H, s, N-CH_3), 2.71 (1.5H, s, N-CH_3), 1.37 (4.5H, s, ^{t}Bu) 1.32 (4.5H, s, ^{t}Bu); δ_F(283 MHz, $CDCl_3$): -118.1 (m, Ar-F); δ_C(101 MHz, $CDCl_3$): 176.4, 162.7, 160.3, 156.1, 155.1, 131.7, 131.4, 128.8, 128.8, 128.7, 128.6, 124.6, 124.5, 124.4, 124.3, 124.2, 115.5, 115.4, 115.3, 115.2, 81.0, 80.7, 60.2, 33.4, 32.9, 29.2, 28.3, 28.2; ν_{max} (film) 2970, 1714, 1693, 1587, 1396, 1234, 1156, 908, 732; HRMS (ESI) calc for $C_{15}H_{20}FNNaO_4$ $[M+Na]^+$ 320.1269, found 320.1278

(±)-*N*-methyl-*p*-fluorophenylalanine 20

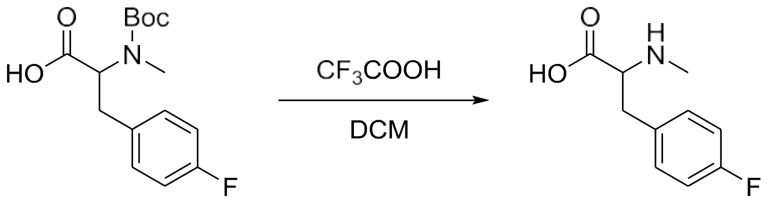

S2 **20**

Protected amino acid **S2** (1.00 g, 3.36 mmol) was dissolved in DCM (4 mL) and trifluoroacetic acid (3 mL) added. The reaction mixture was stirred at r.t. for 16 h, then concentrated *in vacuo,* azeotroping with toluene (3 × 10 mL). The resulting white solid was washed with chloroform and diethyl ether, yielding amino acid **23** as the trifluoroacetic acid salt (550 mg, 53%) δ_H(400 MHz, D_2O): 7.08-7.03 (2H, m, Ar-H), 6.92-9.85 (2H, m, Ar-H) 4.05 (1H, t, *J* 6.0, 2-H), 3.11 (1H, dd, *J* 14.0, 6.0, 3-H), 3.03 (1H, dd, *J* 14.0, 6.0, 3-H), 2.52 (3H, s, N-CH_3); δ_F(283 MHz, D_2O): -75.7 (s, CF_3COOH), -115.1 (tt, *J* 9.0, 5.5, Ar-F); δ_C(101 MHz, D_2O) 170.1, 163.2, 160.8, 131.1, 131.0, 128.9, 128.9, 115.7, 115.5, 61.7, 61.5, 33.7, 31.8, 31.6; ν_{max} (ATR) 2807, 1742, 1651, 1510, 1222, 1187, 1136, 830, 727; HRMS (ESI) calc for $C_{10}H_{13}FNO_2$ $[M+H]^+$ 198.0925, found 198.0918.

(±)-*N*-methyl-*m*-fluorophenylalanine 19

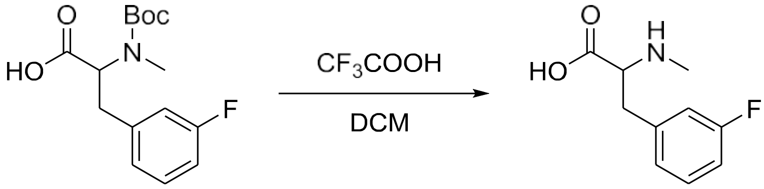

S3 **19**

Prepared as above from protected amino acid **S3** (300 mg, 1.0 mmol), to yield the product trifluoroacetic acid salt **22** as a white solid(149 mg, 47%). δ_H(400 MHz, D_2O) 7.06 (1H, m, Ar-H), 6.79-6.69 (3H, m, Ar-H), 3.97 (1H, t, *J* 6.0, 2-H), 3.03 (1H, dd, *J* 14.0, 6.0, 3-H), 2.96 (1H, dd, *J* 14.0, 6.0, 3-H), 2.42 (3H, s, N-CH_3); δ_F(283 MHz, D_2O) -75.9 (s, CF_3COOH), -113.2 (m, Ar-F); δ_C(101 MHz, $CDCl_3$): 169.9, 163.6, 161.8, 161.4, 161.2, 135.5, 135.4, 130.8, 130.5, 125.0, 116.9, 116.0, 115.9, 115.8, 115.6, 114.7, 114.5, 114.0, 62.4, 61.2, 34.1, 33.9, 31.8, 31.5; ν_{max} (ATR) 3023, 2809, 1729, 1665, 1588, 1437, 1198, 1144; HRMS (ESI) calc for $C_{10}H_{13}FNO_2$ $[M+H]^+$ 198.0925, found 198.0923.

(±)-*N*-methyl-*o*-fluorophenylalanine 18

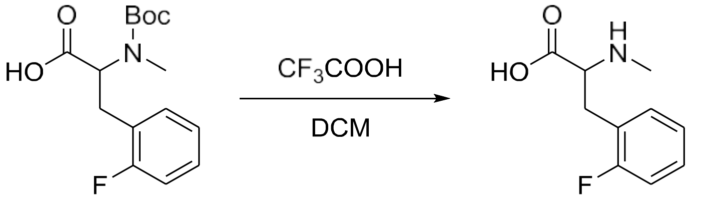

S4 **18**

Prepared as above from protected amino acid **S4** (395 mg, 1.33 mmol), to yield the product trifluoroacetic acid salt **21** as a white solid (238 mg, 57%). δ_H(400 MHz, D_2O) 7.11-6.96 (2H, m, Ar-H), 6.91-6.80 (2H, m, Ar-H), 3.98 (1H, m, 2-H), 3.08-3.01 (2H, m, 3-H_2), 2.47 (3H, s, N-CH_3); δ_F(283 MHz, D_2O) -75.8 (CF_3COOH), -117.4 (Ar-F); δ_C(101 MHz, D_2O) 169.9, 162.0, 159.6, 131.6, 131.4, 130.2, 124.6, 120.1, 119.9, 115.5, 115.2, 60.8, 60.6, 31.9, 31.6, 28.5; ν_{max} (ATR) 2747, 1790, 1596, 1495, 1397, 1232, 1182, 1100, 758; HRMS (ESI) calc for $C_{10}H_{13}FNO_2$ $[M+H]^+$ 198.0925, found 109.0924

(±)-*N*-methyl-*p*-methylphenylalanine 15

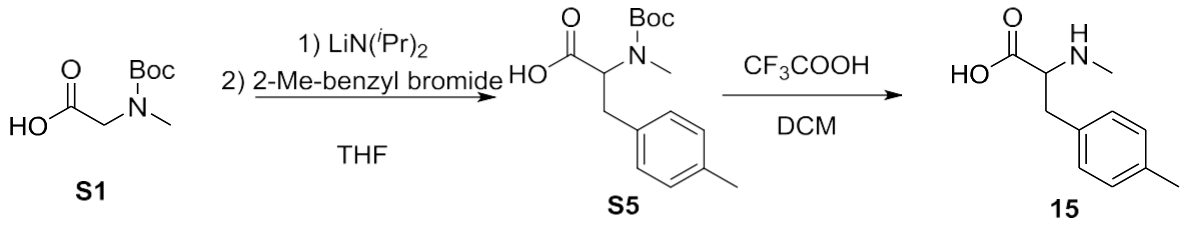

(±)-*N*-(*tert*-butoxycarbonyl)-*N*-methyl-*p*-methylphenylalanine **S5** was prepared similarly from *N*-Boc sarcosine **S1** (500 mg, 2.64 mmol) and 2-fluorobenzyl bromide (1.3 mL, 10.6 mmol), yielding the product as a white solid (504 mg, 64%) which was used without purification. (±)-*N*-methyl-p-methylphenylalanine **18** was prepared as above from protected amino acid **S5** (500 mg, 1.70 mmol), to yield the product trifluoroacetic acid salt **18** as a white solid (203 mg, 62%). δ_H (400 MHz, D_2O 7.10 (2H, d, *J* 7.9 Hz, Ar-H), 7.05 (2H, d, *J* 7.9 Hz, Ar-H), 3.74 (1H, t, *J* 6.2 Hz, 2-H), 3.05 (2H, d, *J* 6.2 Hz, 3-H_2), 2.54 (3H, s, N-H_3), 2.18 (3H, s, CH_3).δ_C (126 MHz, D_2O) 172.6 (C=O), 137.9 (Ar), 131.1 (Ar), 129.6 (Ar-CH_2), 129.3 (Ar-CH_2), 64.3 (CH), 35.1 (CH_2), 31.2 (N-CH_3), 20.1 (CH_3); ν_{max} (ATR) 3098, 3026, 2930, 1729, 1647, 1592, 1513, 1418, 1386, 1145; HRMS (ESI) calc for $C_{11}H_{16}NO_2$ $[M+H]^+$ 194.1103, found 194.1176.

(*S*)-(-)-*N*-(*tert*-butoxycarbonyl)-*N*-methyl-*p*-fluorophenylalanine (*S*)-S2

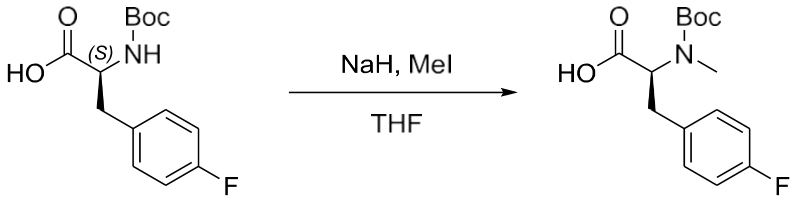

S5 **(*S*)-S2**

Sodium hydride (60% dispersion in mineral oil, 4.21 g, 106 mmol, 10 equiv.) was added slowly in portions over 1 h to a solution of L-*N*-Boc-*p*-fluorophenylalanine **S5** (3001 mg, 10.6 mmol) and iodomethane (6.5 mL, 106 mmol, 10 equiv.) in THF (8 mL) at 0 °C. The reaction mixture was allowed to warm to room temperature and stirred overnight. The reaction mixture was diluted with diethyl ether (40 mL) and quenched with water (15 mL). The layers were separated, and the aqueous layer extracted with diethyl ether (2 × 30 mL), acidified with 10% citric acid solution, and extracted with ethyl acetate (3 × 60 mL). The combined organic layers were dried over $MgSO_4$, and concentrated *in vacuo* to yield an orange oil. The crude material was purified via column chromatography, eluting with 20-30% ethyl acetate/petrol with 1% acetic acid, to yield L-*N*-Me-(4-F)-Phe **(*S*)-S2** (3.02 g, 96%); Spectroscopic data as for **S2**. [□]$^{22}_{D}$ -52 (*c* 1, $CHCl_3$).

(*S*)-(+)-*N*-methyl-*p*-fluorophenylalanine (*S*)-23

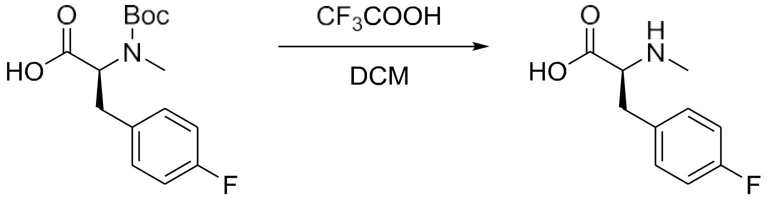

(*S*)-S2 **(*S*)-23**

Prepared as above from protected amino acid **(*S*)-S2** (456 mg, 1.54 mmol), to yield amino acid trifluoroacetic acid salt **(*S*)-23** as a white solid (157 mg, 52%). Spectroscopic data in agreement with **23**. [□]$^{22}_{D}$ +9.0 (*c* 1, 1 M HCl).

(*S*)-(+)-*N*-ethylphenylalanine 19

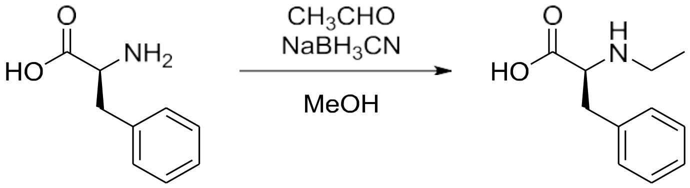

S6 **19**

To a suspension of L-phenylalanine **S6** (507 mg, 3.03 mmol) in methanol (6 mL) was added acetaldehyde (0.2 mL, 158 mg, 3.58 mmol, 1.2 equiv.) and sodium cyanoborohydride (141 mg, 2.24 mmol, 0.74 equiv.) and the reaction mixture stirred at room temperature for 18 h. The resulting white precipitate was isolated by vacuum filtration and washed with methanol to yield pure (*S*)-*N*-ethylphenylalanine **19** (359 mg, 61%). δ_H (400 MHz, D_2O) 7.35-7.27 (3H, m, Ar-H), 7.25-7.19 (2H, m, Ar-H) 4.23 (1H, t, *J* 6.5, CH), 3.29 (1H, dd, *J* 14.5, 5.5, ArC*H*H), 3.18 (1H, dd, *J* 14.5, 7.0, ArCH*H*), 3.07 (2H, q, *J* 7.5, CH_2), 1.17 (3H, t, *J* 7.5, CH_3); δ_C(100 MHz, D_2O) 170.5 (C=O), 133.4 (Ar), 129.2 (Ar), 129.0 (Ar), 127.9 (Ar), 60.4 (CH), 42.1 (CH_2), 34.7 ($ArCH_2$), 10.4 (CH_3); [□]$^{22}_{D}$ +25.0 (*c* 1, 2 M HCl) [lit. [□]$_D$ +33.0 (*c* 1, 2 M HCl)].[20]

NMR data in accordance with literature.[21]

(*S*)-(-)-*N*-(*tert*-butoxycarbonyl)-*N,O*-dimethyltyrosine (*S*)-S9

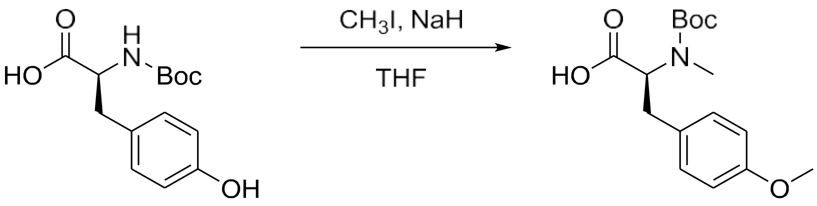

S8 **(*S*)-S9**

A solution of *N*-Boc-L-Tyrosine **S8** (500 mg, 1.77 mmol) and methyl iodide (560 mg, 0.24 mL, 3.91 mmol) in THF (10 mL) at 0 °C, sodium hydride (234 mg, 5.84 mmol) was added. After 1 h at 0 °C the mixture was stirred at room temperature for 12 h. The mixture was quenched by the dropwise addition of THF/Water (1:1, 10 mL). The solvent was removed under reduced pressure. The residue was washed with water (20 mL) and pentane (20 mL). The aqueous phase was acidified with citric acid (pH 2) and extracted with ethyl acetate (20 mL). The combined extracts were washed with aqueous sodium chloride, dried over magnesium sulphate and concentrated under vacuum to obtain pure **(*S*)-S9** (501 mg, 91%) as a yellow oil. δ_H (500 MHz, $CDCl_3$) 7.12 and 7.10 (2H, two d, *J* 8.5 Ar-H), 6.83 (2H, d, *J* 8.5, Ar-H), 4.78 (0.5H, dd, *J* 11.0, 5.1, 2-H), 4.55 (0.5H, dd, *J* 11.0, 4.5, 2-H), 3.78 (3H, s, OCH3), 3.25 (1H, m, 3-H), 3.01 (1H, m, 3-H), 2.75 (1.5H, s, N-CH_3), 2.69 (1.5H, s, N-CH_3), 1.40 (4.5H, s, ^{t}Bu), 1.35 (4.5H, s, ^{t}Bu); δ_C(126 MHz, D_2O) 176.4 (C=O), 176.1 (C=O) , 158.6 (ArOMe), 158.5 (ArOMe), 156.6 (COOBoc), 156.2 (COOBoc), 130.1 (ArH), 130.0 (Ar), 129.6 (Ar) 129.2 (Ar), 114.2 (ArH), 114.0 (ArH), 80.8 (O*C*(CH_3)$_3$), 80.7 (O*C*(CH_3)$_3$), 61.7 (CH), 60.9 (CH), 55.4 (OCH_3), 34.5 (CH_2), 34.0 (CH_2), 33.0 (N-CH_3), 32.7 (N-CH_3), 28.4 (OC(*C*H_3)$_3$), 28.3 (OC(*C*H_3)$_3$); $[\square]^{21}_{D}$ -15.3 (*c* 1.0, MeOH) [lit. $[\square]^{22}_{D}$ -16.9 (*c* 1.0, MeOH)].[22]

(*S*)-(+)- *N,O*-dimethyltyrosine 17

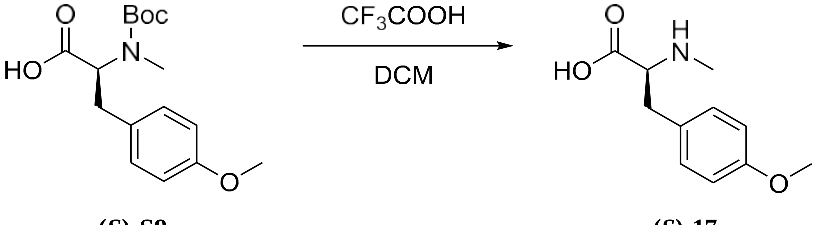

(*S*)-S9 **(*S*)-17**

Prepared as above from protected amino acid **(*S*)-S9** (400 mg, 1.30 mmol), to yield amino acid trifluoroacetic acid salt **(*S*)-20** as a white solid (151 mg, 55%). δ_H (500 MHz, D_2O) 7.25 (2H, d, *J* 8.6, Ar-H), 7.00 (2H, d, *J* 8.6, Ar-H), 4.11 (1H, t, *J* 6.1, 2-H), 3.83 (3H, s, OCH_3), 3.28 (1H, dd, *J* 14.8, 6.1, 3-H), 3.23 (1H, dd, *J* 14.8, 6.1, 3-H),

2.73 (3H, s, N-CH_3); δ_C(126 MHz, D_2O) 171.3 (C=O), 158.4 (ArOMe), 130.6 (ArH), 126.1 (Ar), 114.5 (ArH), 62.9 (CH), 55.3 (OMe), 34.2 (CH_2), 31.9 (NMe);); [□]$^{24}_{D}$ +8.1 (*c* 0.3, H_2O) [lit. [□]$^{20}_{D}$ +7.5 (*c* 0.3, H_2O)].[23]

4-Fluoro-*N*-(tert-butoxycarbonyl)-*N*-methylphenylalanine

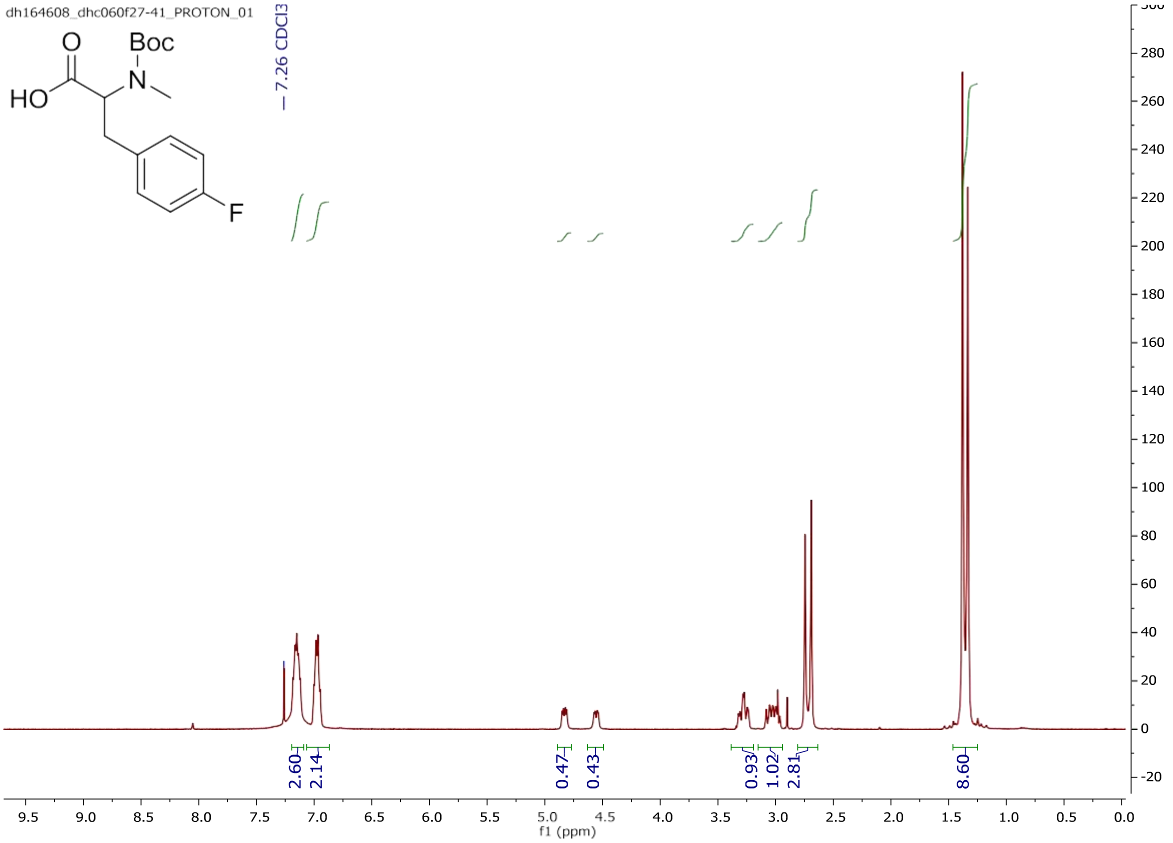

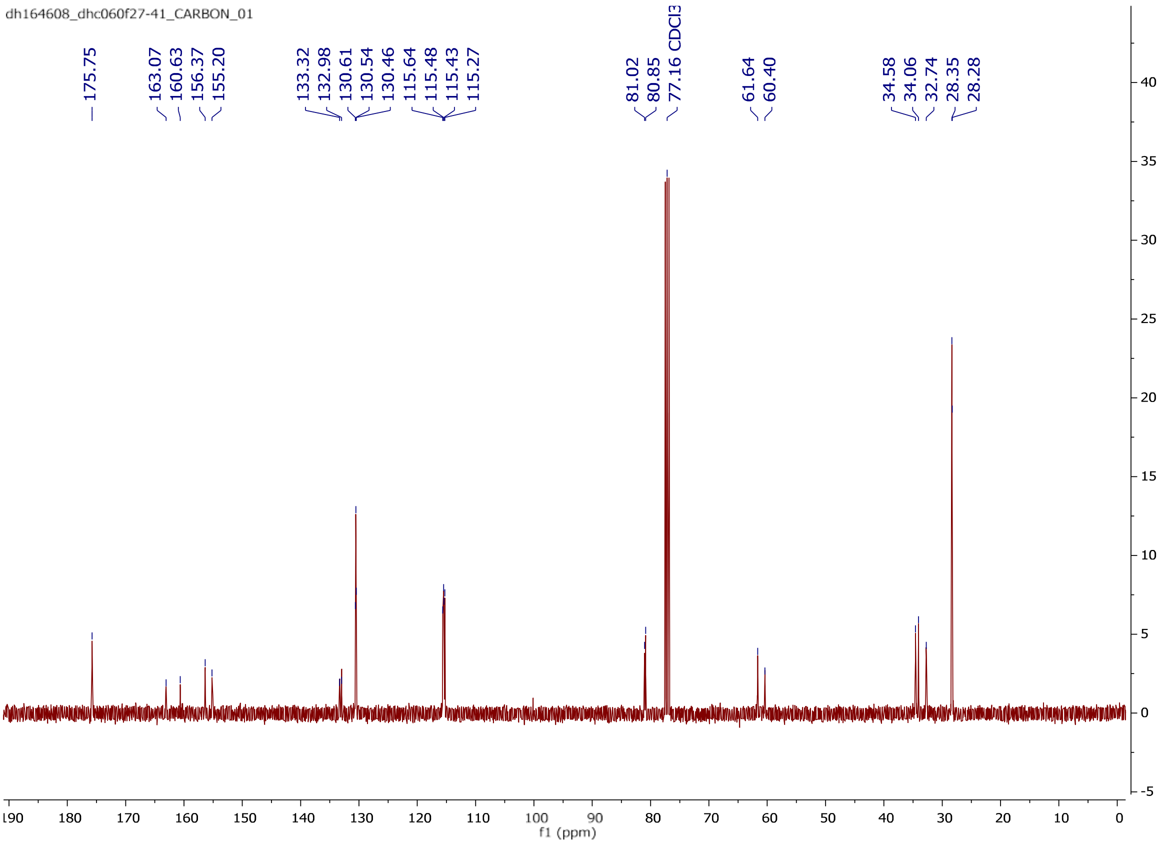
dh164608_dhc060f27-41_CARBON_01
175.75
163.07
160.63
156.37
155.20
133.32
132.98
130.61
130.54
130.46
115.64
115.48
115.43
115.27
81.02
80.85
77.16 CDCl3
61.64
60.40
34.58
34.06
32.74
28.35
28.28
f1 (ppm)

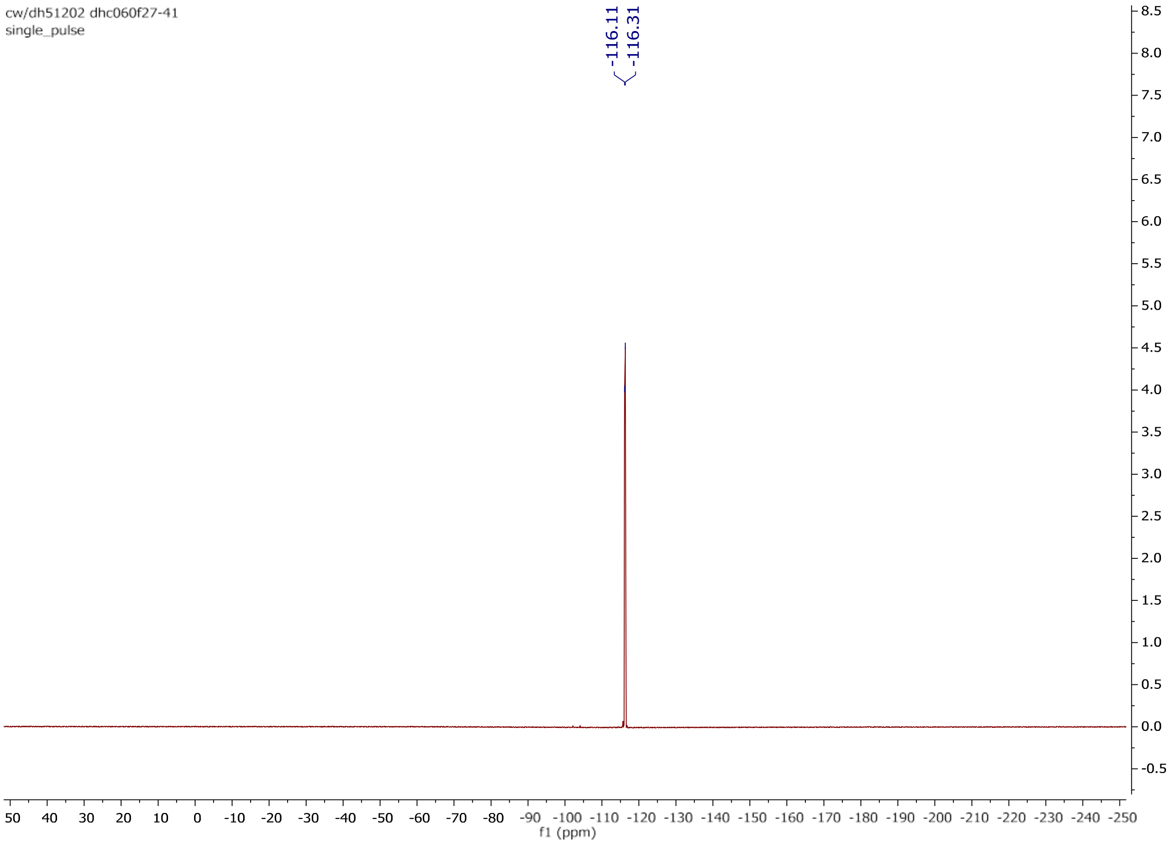
cw/dh51202 dhc060f27-41
single_pulse
-116.11
-116.31
f1 (ppm)

4-Fluoro-*N*-methylphenylalanine

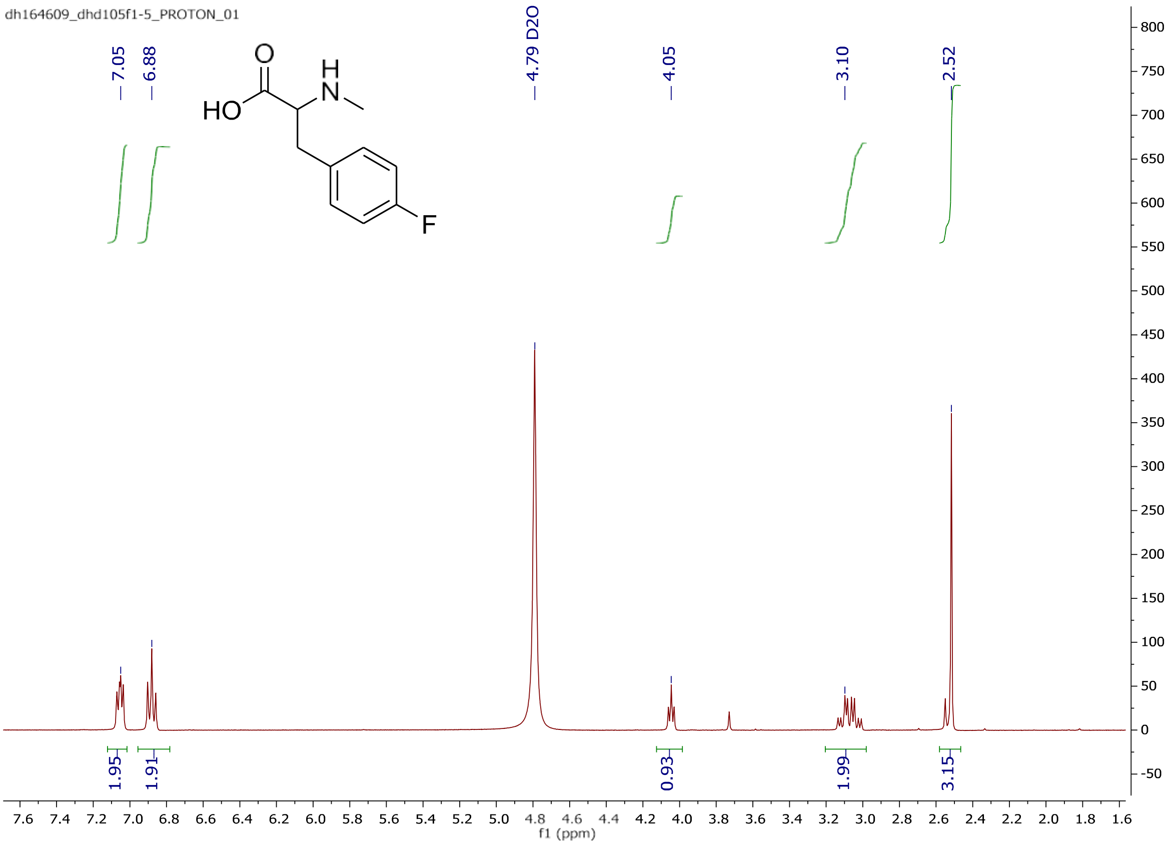

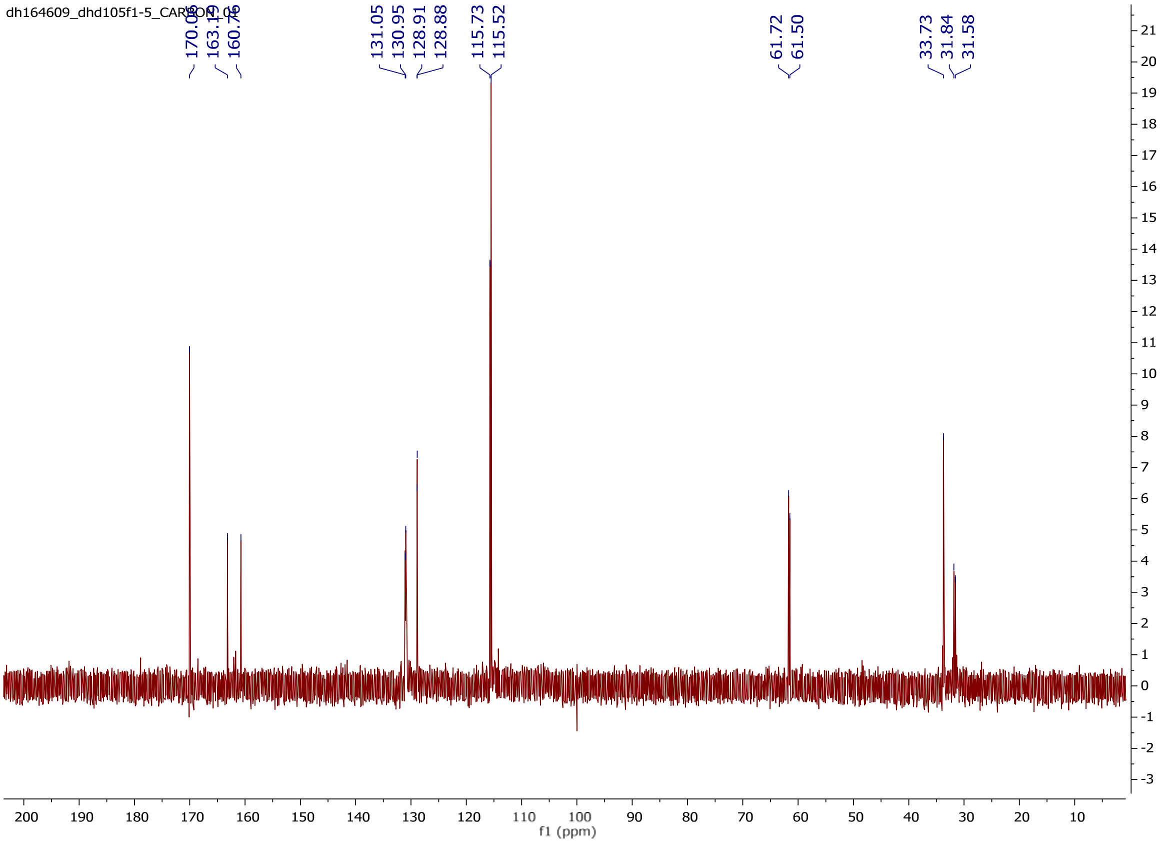

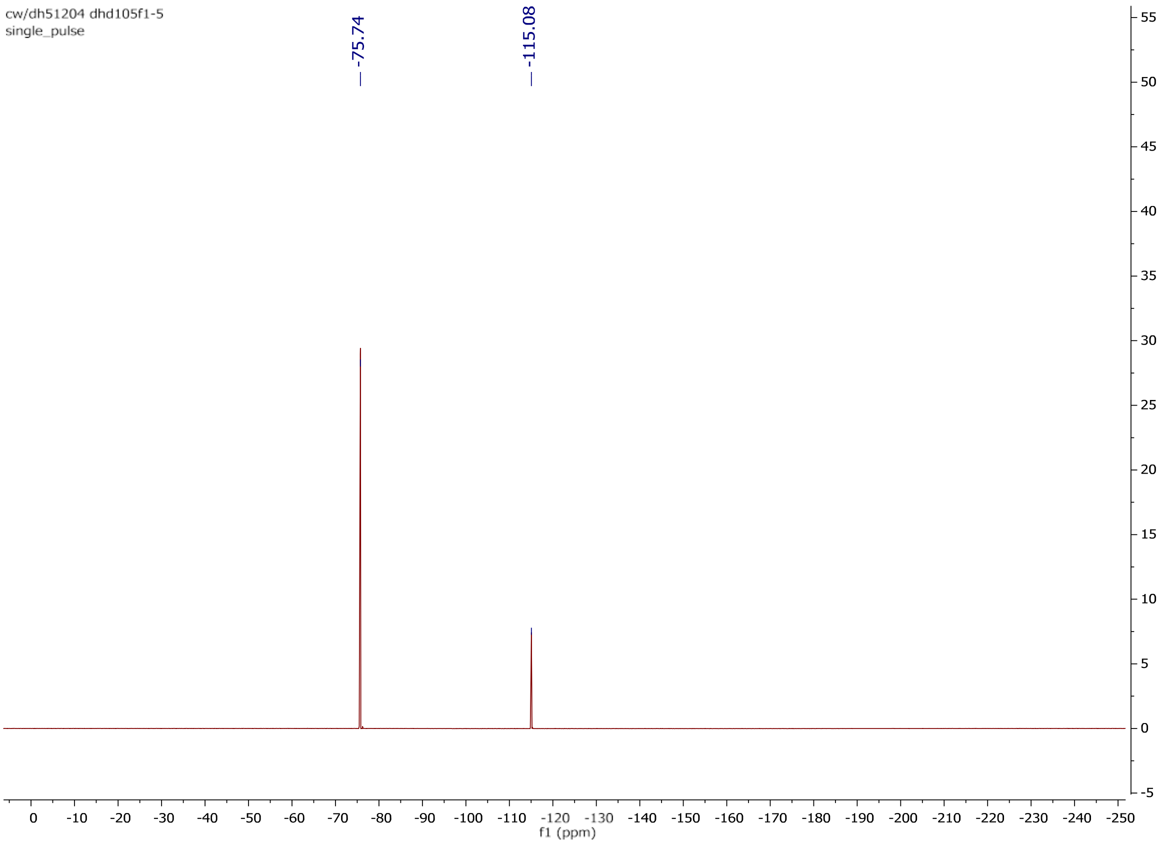

3-Fluoro-*N*-(tert-butoxycarbonyl)-*N*-methylphenylalanine

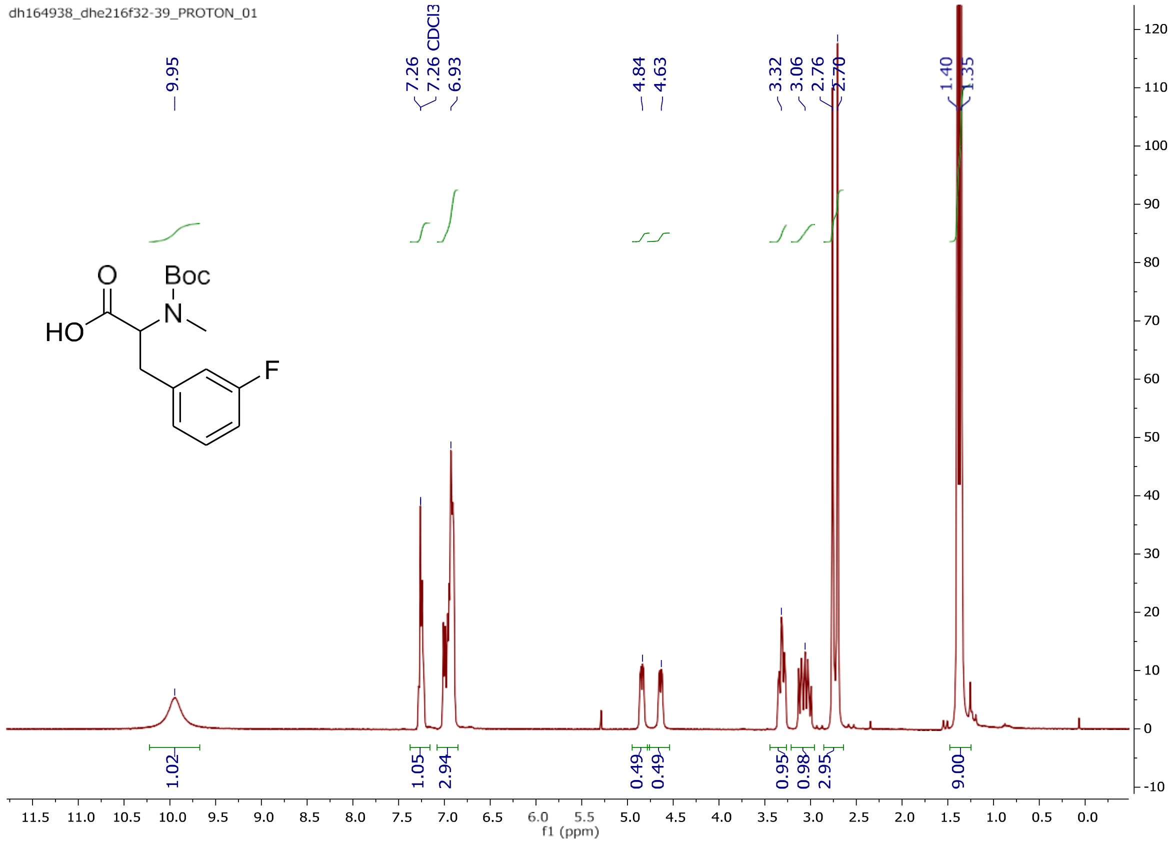

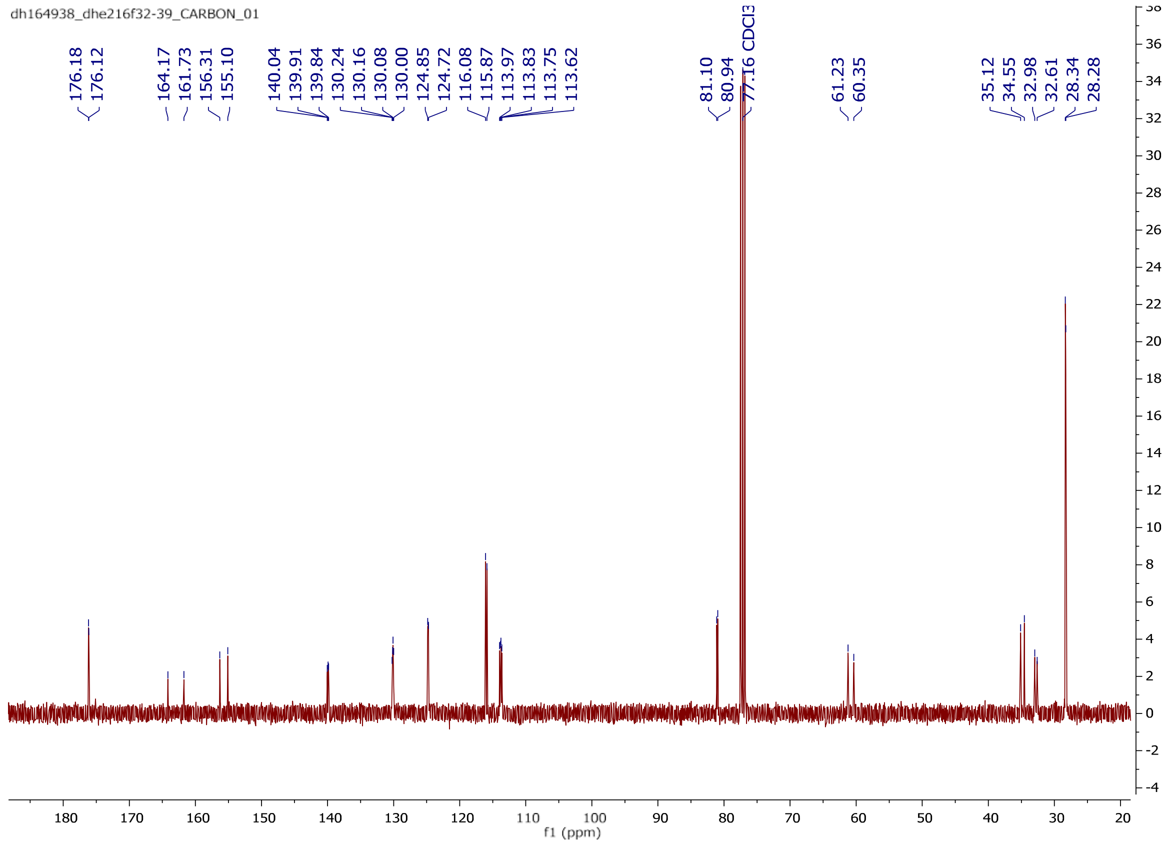

dh164938_dhe216f32-39_CARBON_01
176.18
176.12
164.17
161.73
156.31
155.10
140.04
139.91
139.84
130.24
130.16
130.08
130.00
124.85
124.72
116.08
115.87
113.97
113.83
113.75
113.62
81.10
80.94
77.16 CDCl3
61.23
60.35
35.12
34.55
32.98
32.61
28.34
28.28
f1 (ppm)

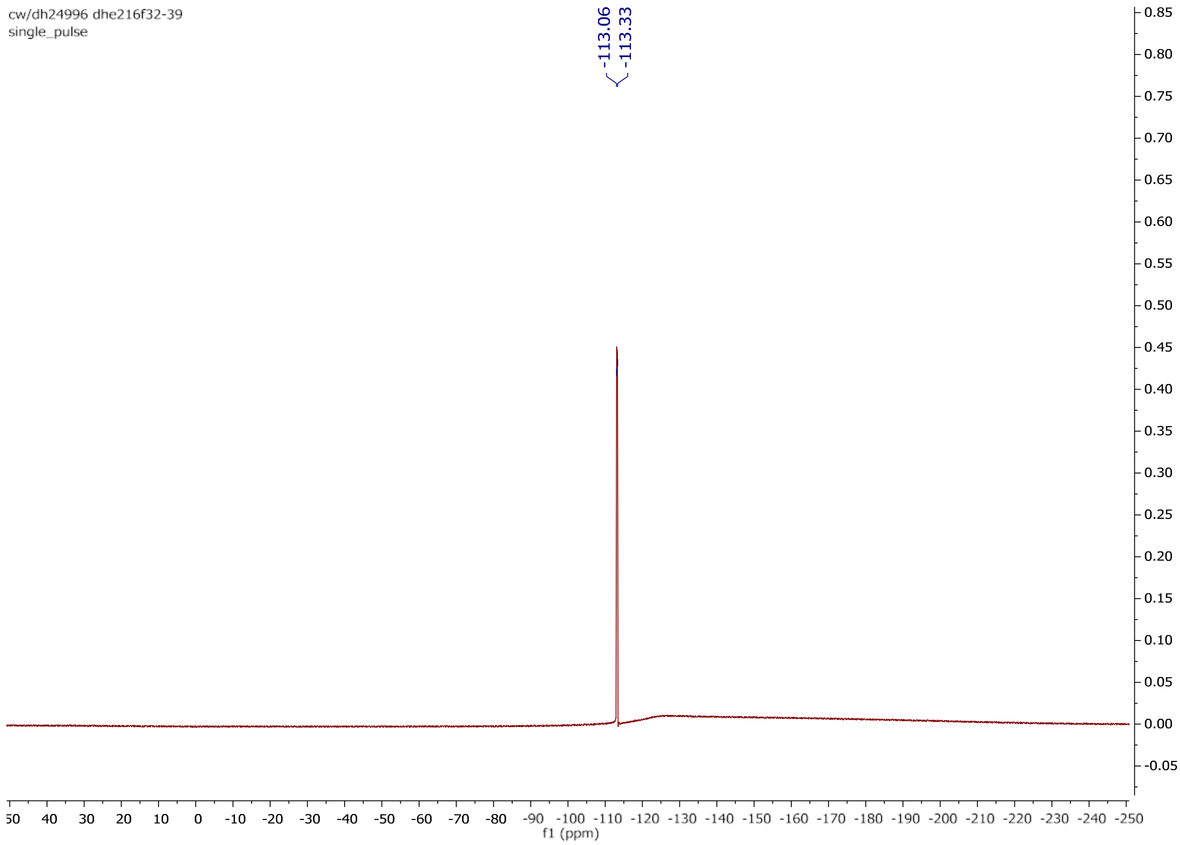

cw/dh24996 dhe216f32-39
single_pulse
-113.06
-113.33
f1 (ppm)

3-Fluoro-*N*-methylphenylalanine

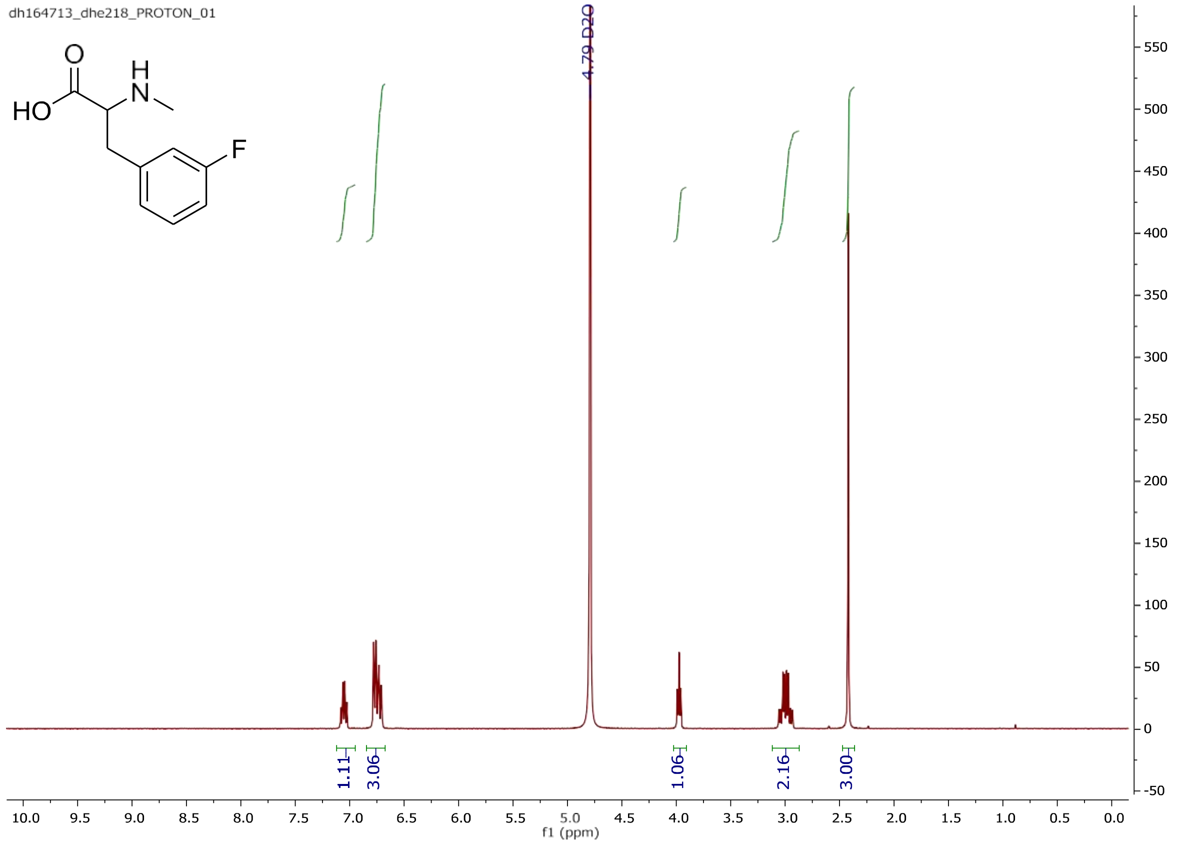

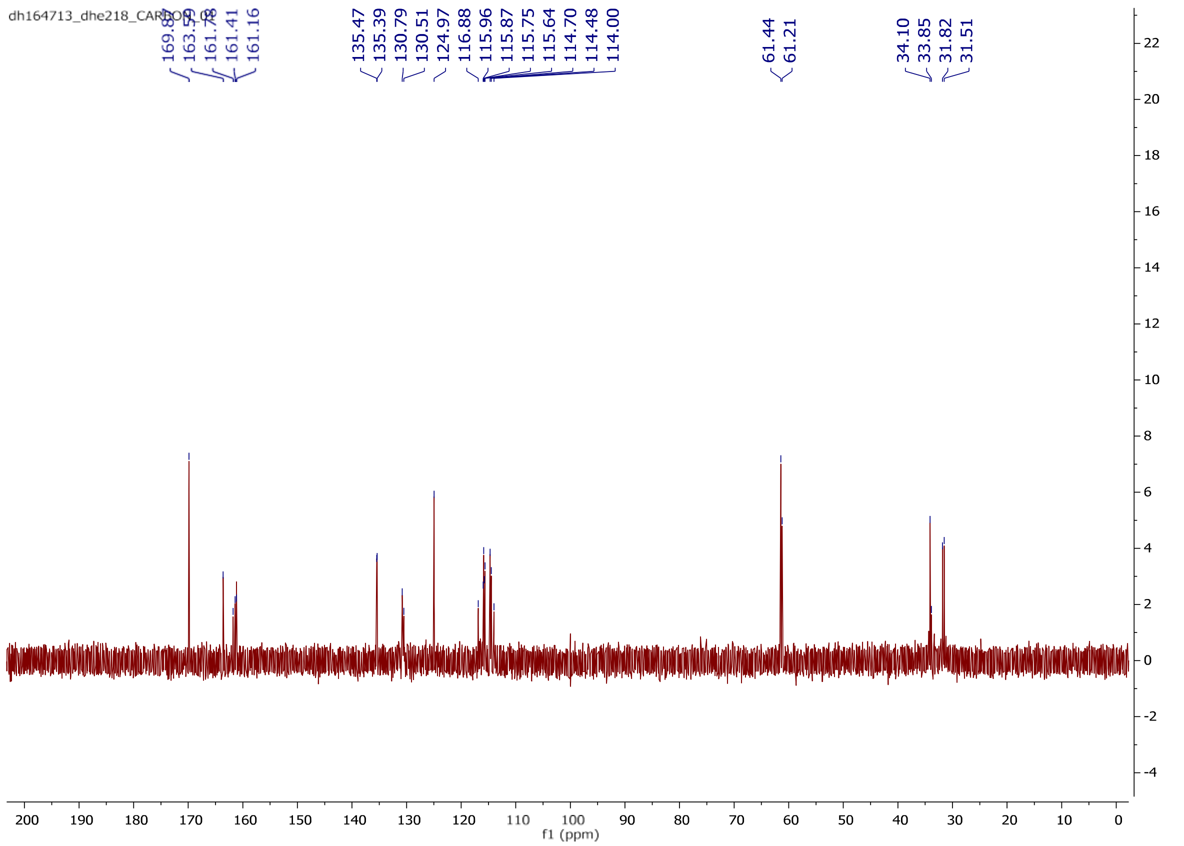

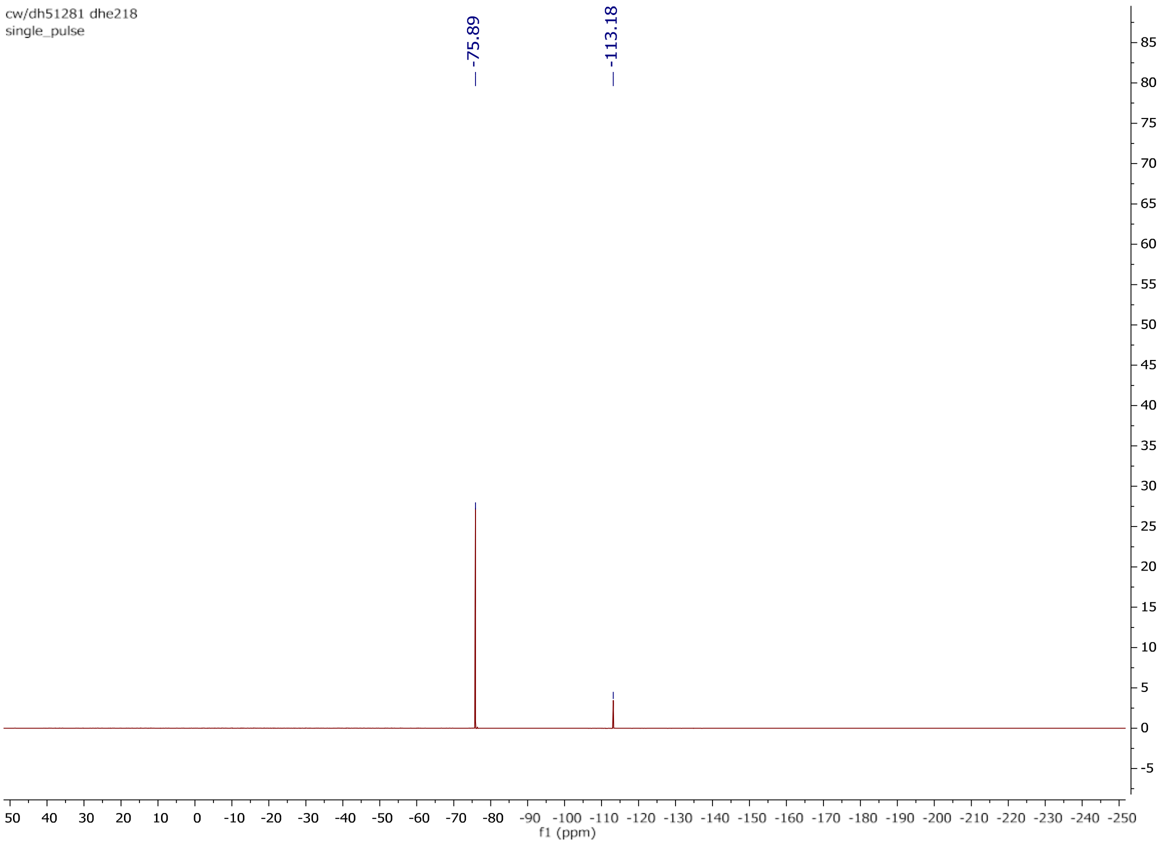

2-Fluoro-*N*-(tert-butoxycarbonyl)-*N*-methylphenylalanine

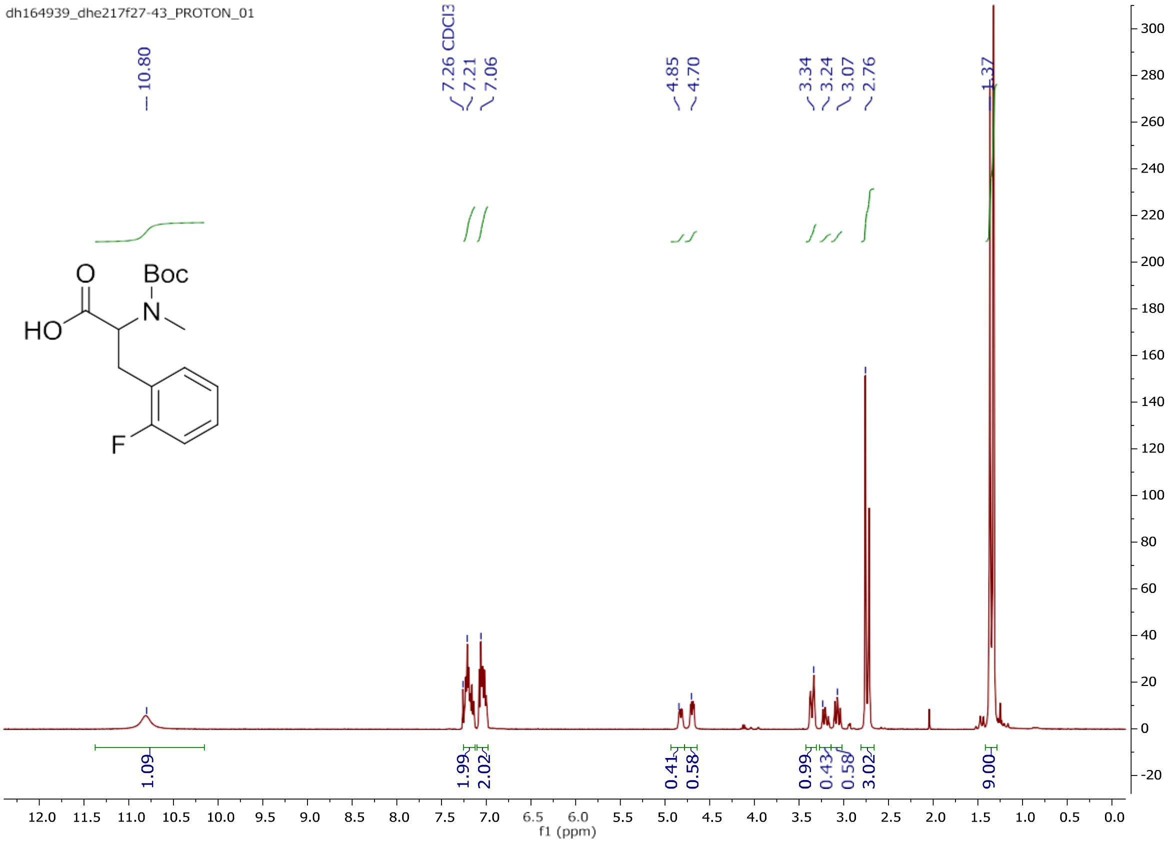

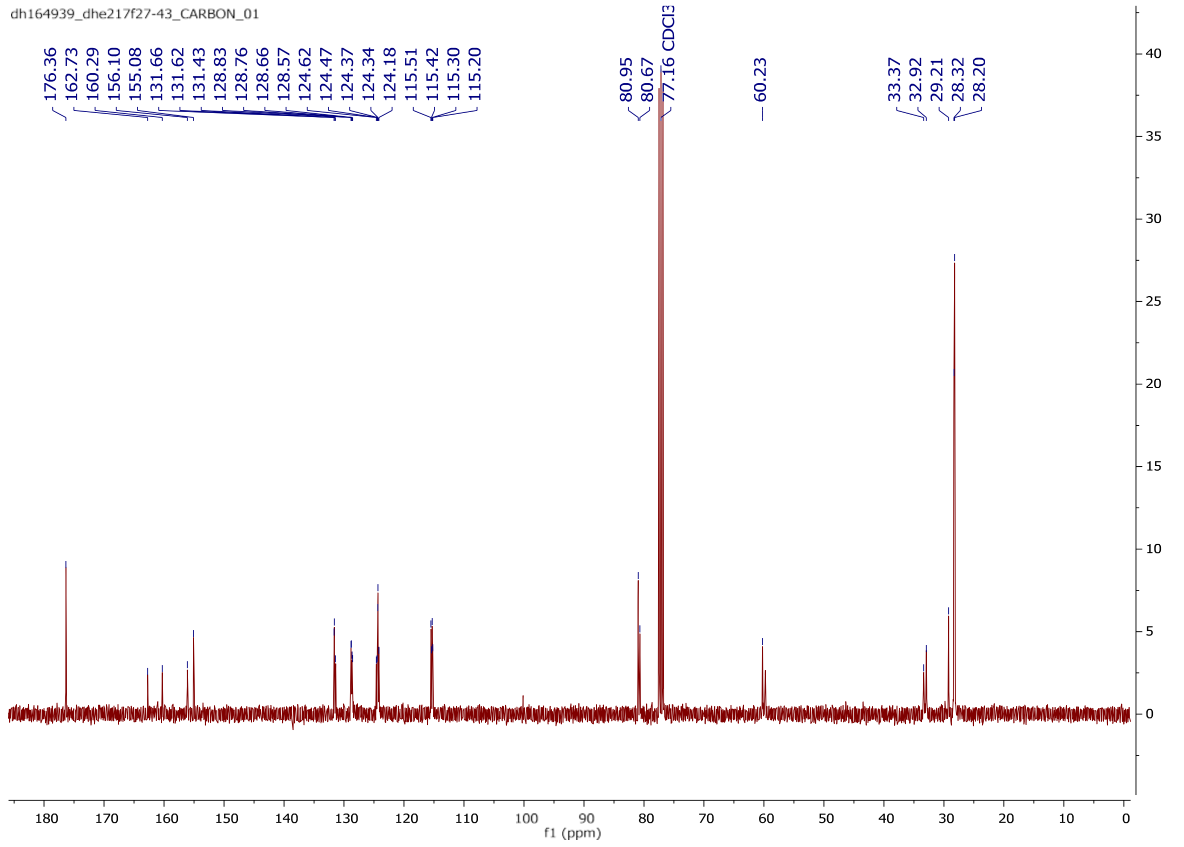
dh164939_dhe217f27-43_CARBON_01
176.36
162.73
160.29
156.10
155.08
131.66
131.62
131.43
128.83
128.76
128.66
128.57
124.62
124.47
124.37
124.34
124.18
115.51
115.42
115.30
115.20
80.95
80.67
77.16 CDCl3
60.23
33.37
32.92
29.21
28.32
28.20
f1 (ppm)

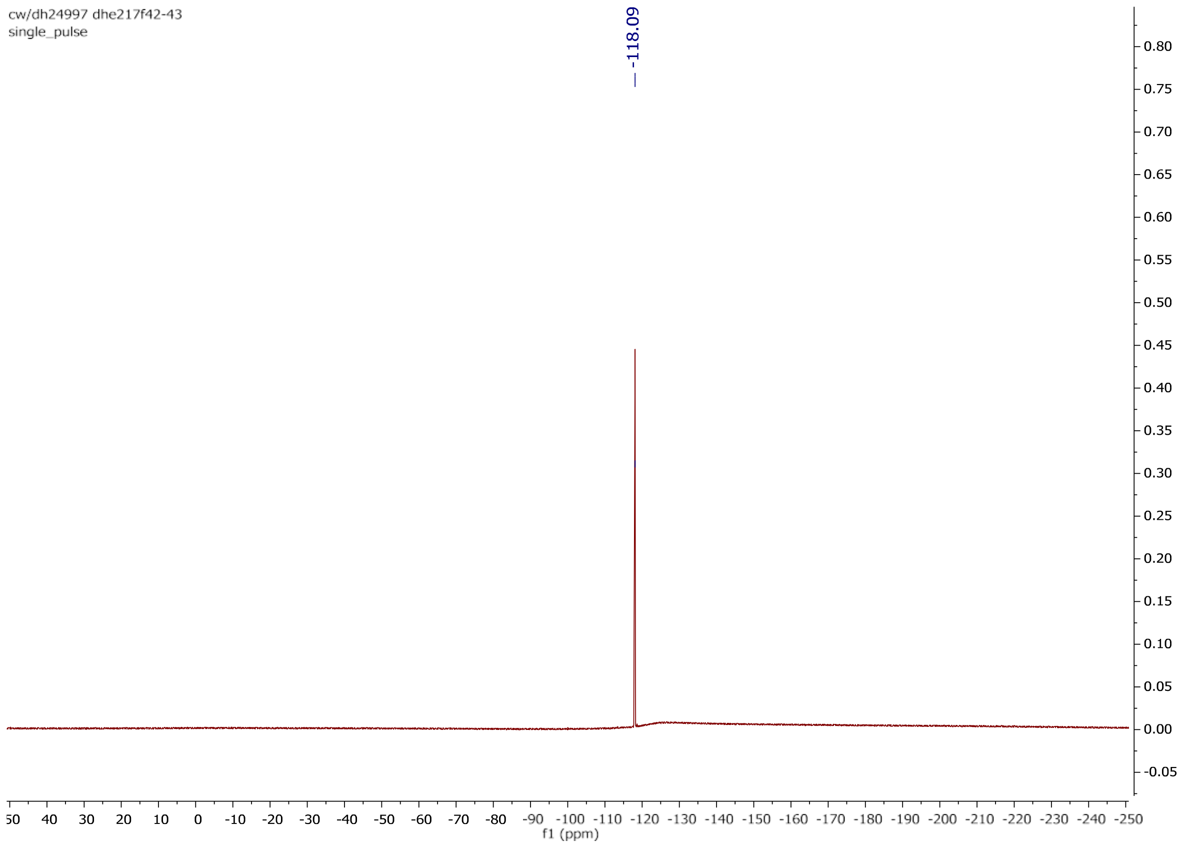
cw/dh24997 dhe217f42-43
single_pulse
-118.09
f1 (ppm)

2-Fluoro-*N*-methylphenylalanine

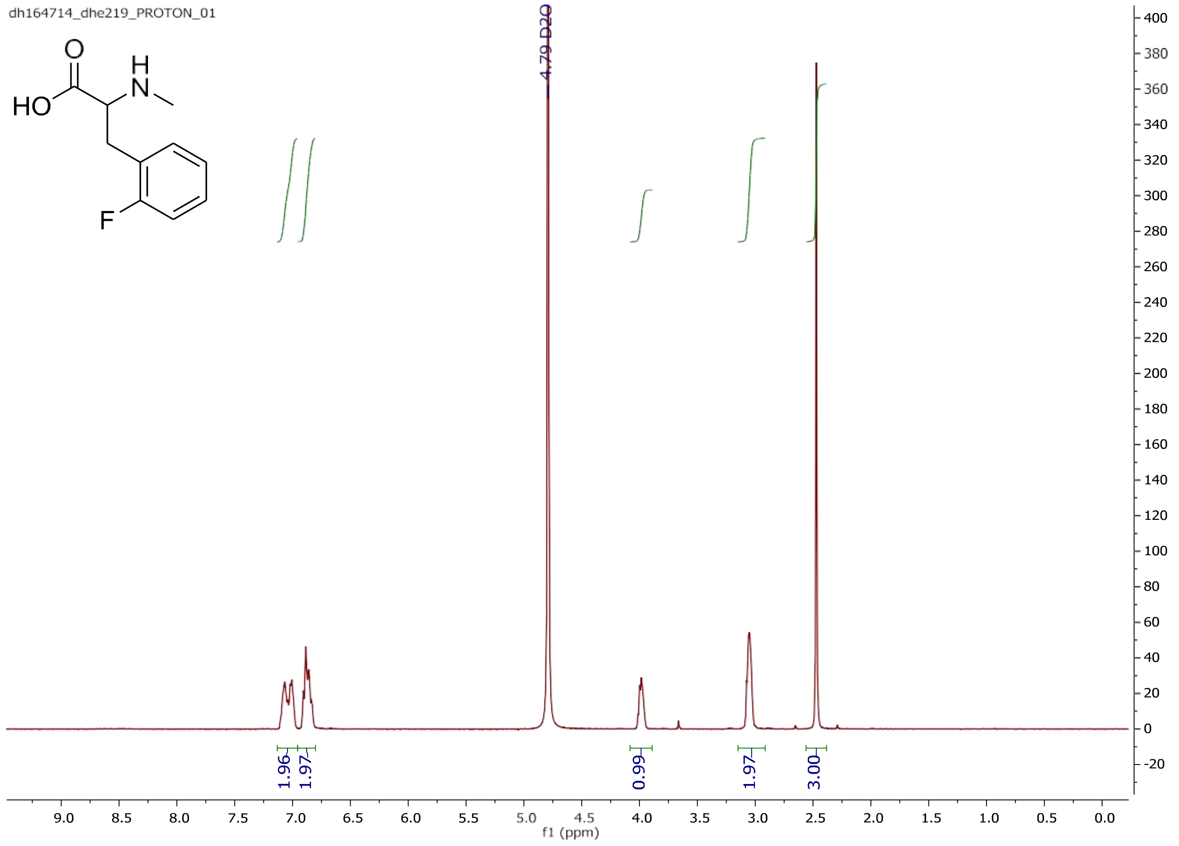

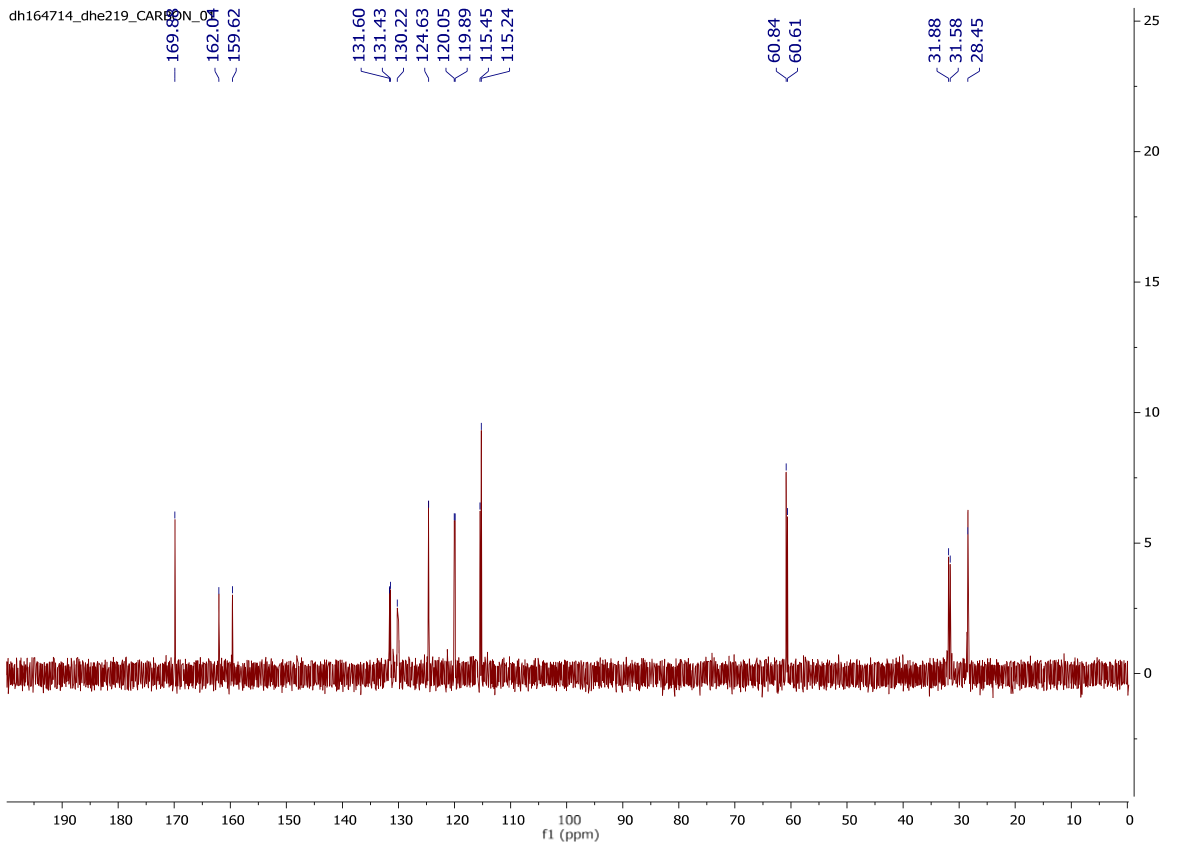

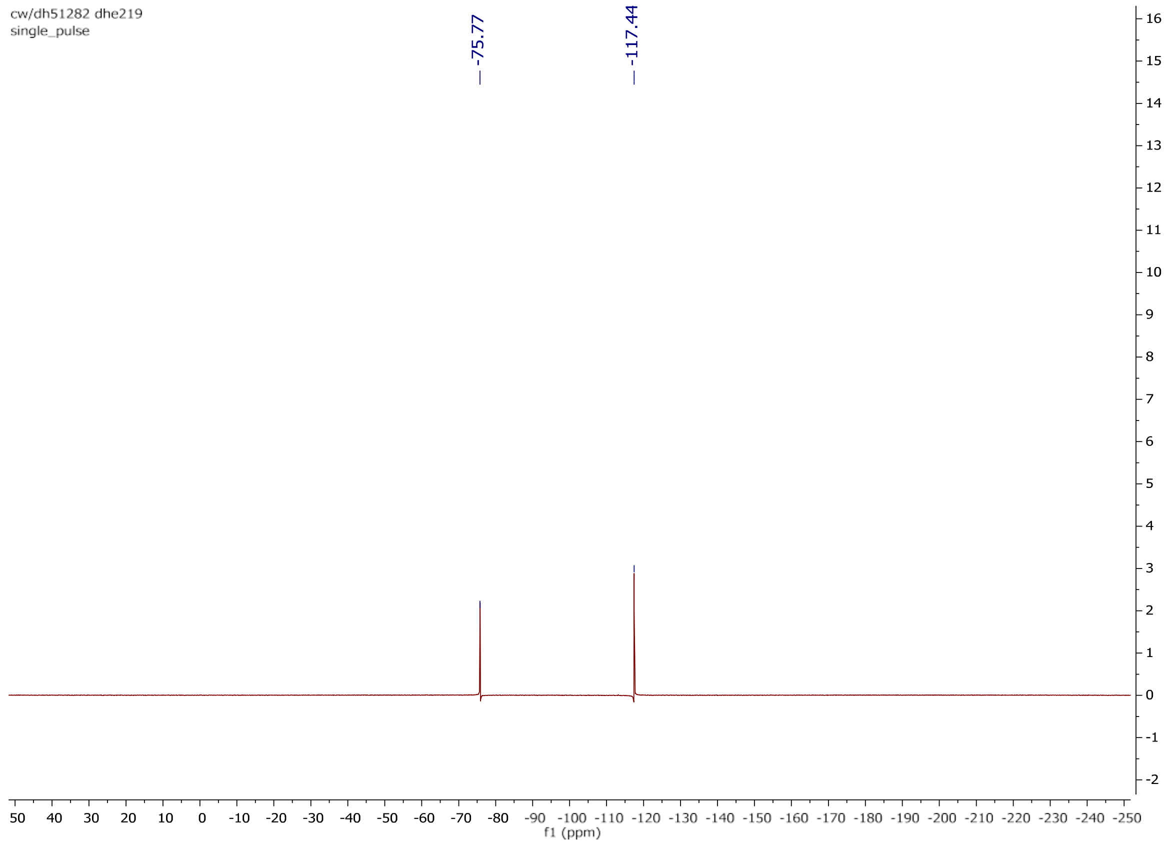

cw/dh51282 dhe219
single_pulse
-75.77
-117.44
f1 (ppm)

(±)-*N*-methyl-*p*-methylphenylalanine

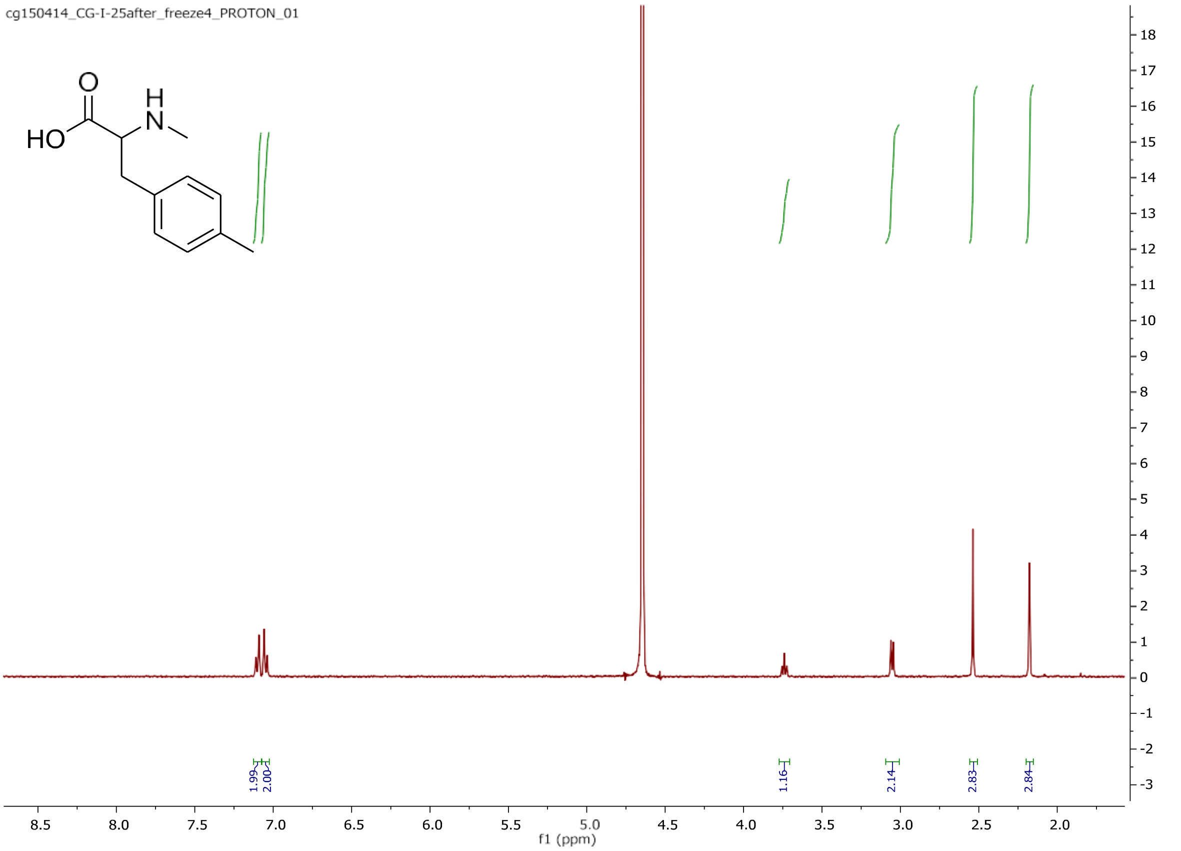

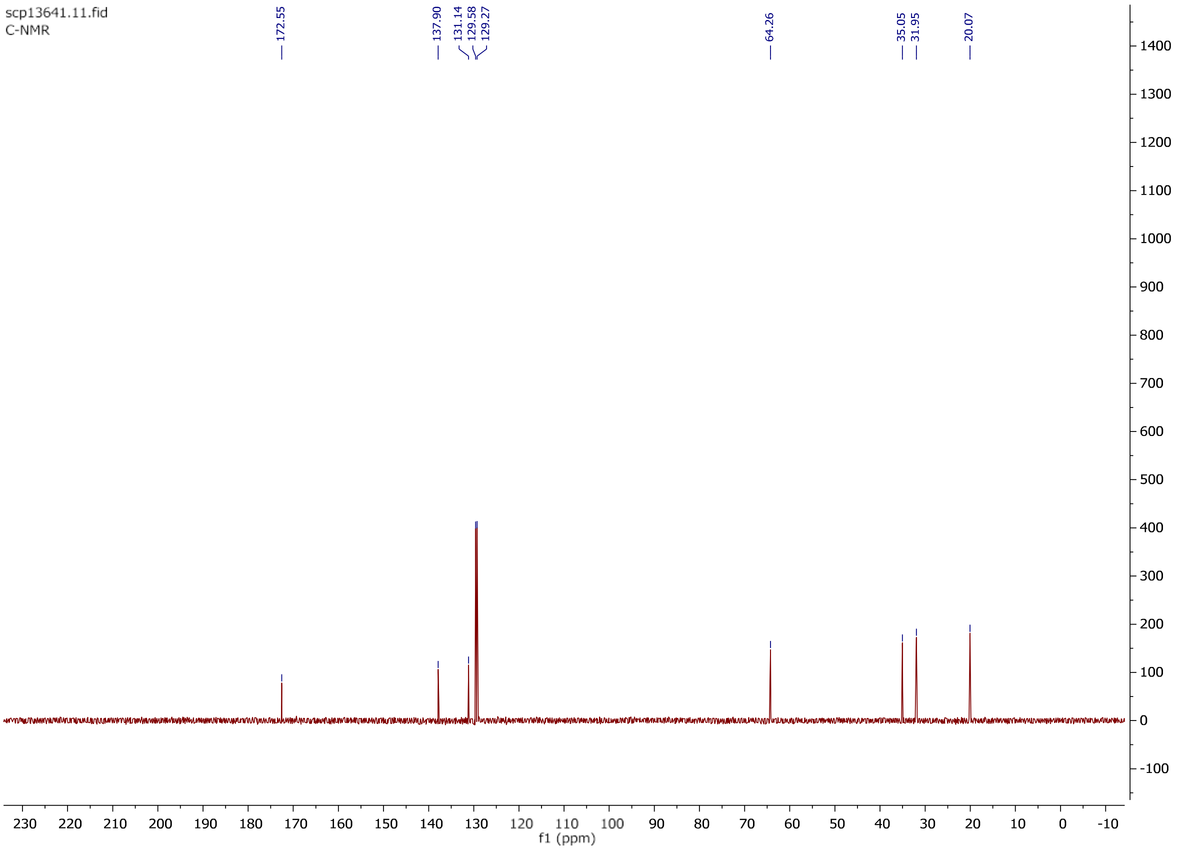

(*S*)-(+)-*N*-ethylphenylalanine

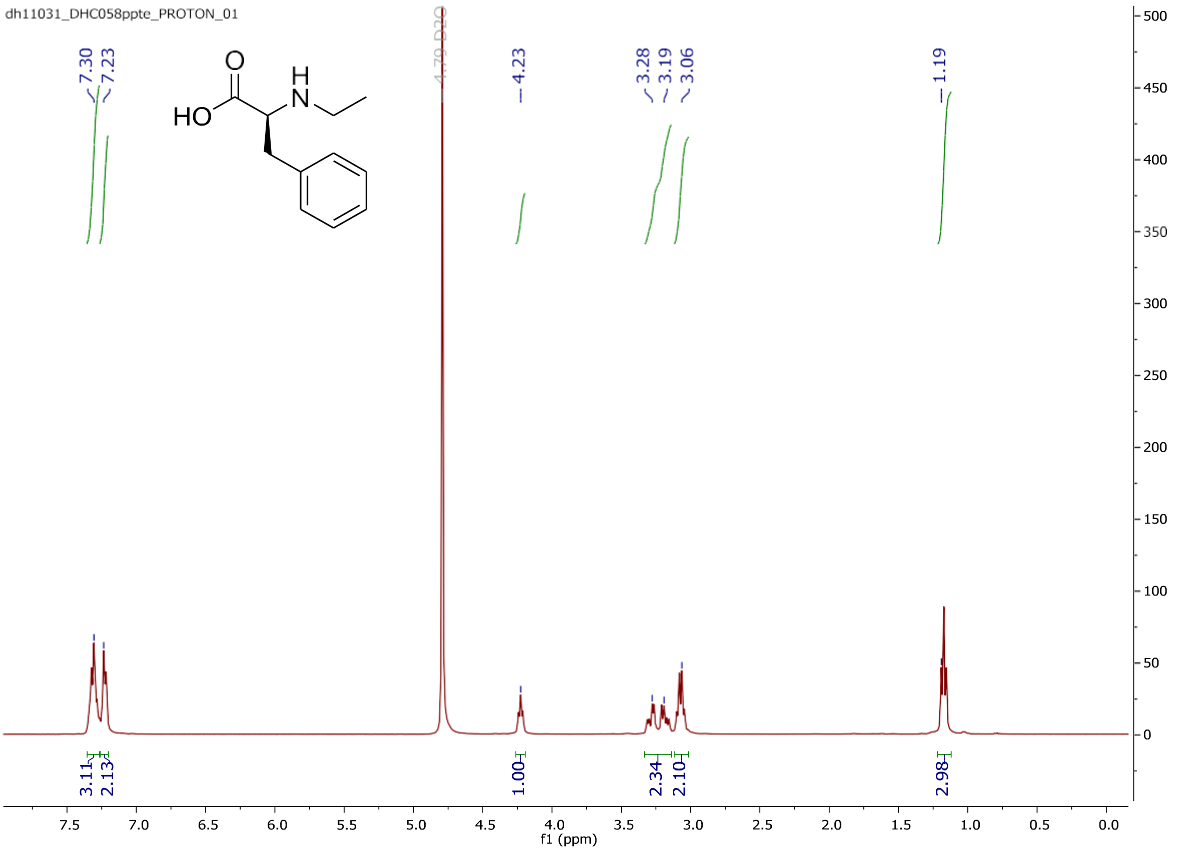
dh11031_DHC058ppte_PROTON_01
7.30
7.23
4.79 D2O
4.23
3.28
3.19
3.06
1.19
HO
O
H
N
3.11
2.13
1.00
2.34
2.10
2.98
f1 (ppm)

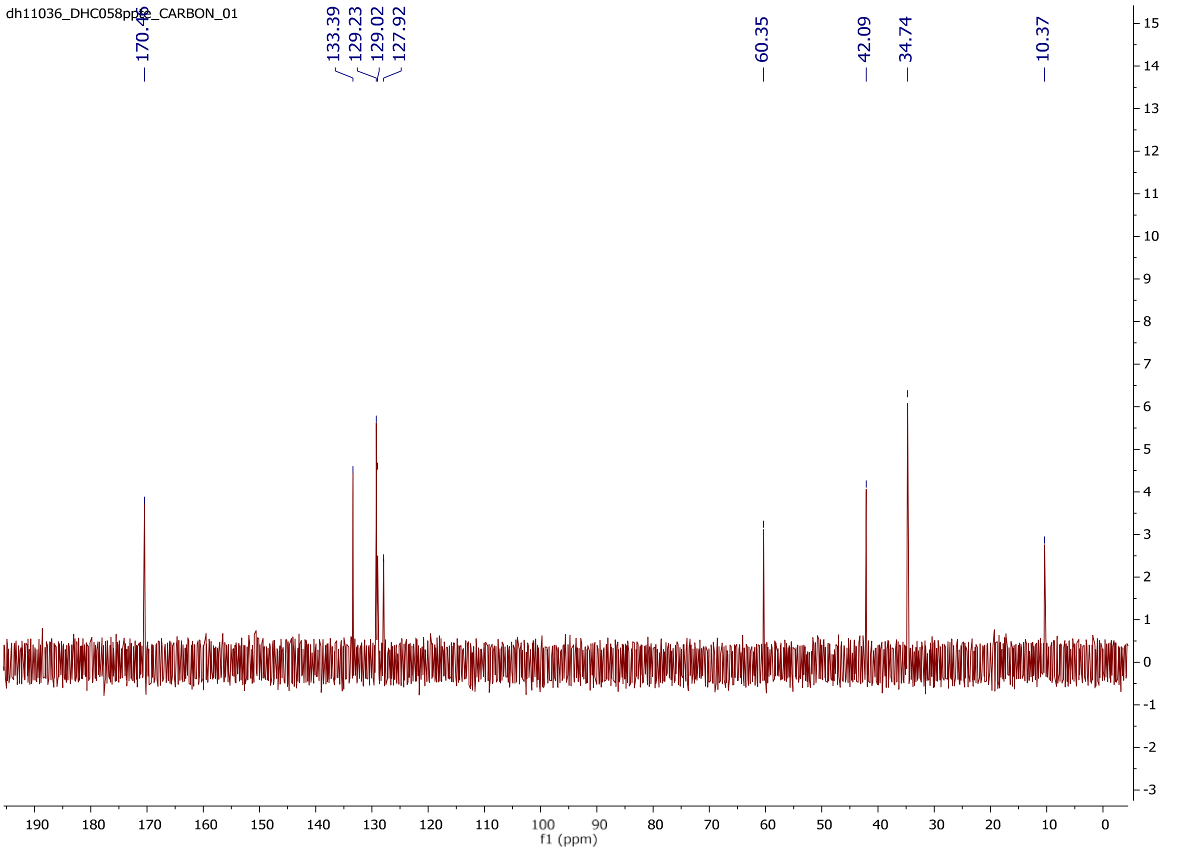
dh11036_DHC058ppte_CARBON_01
170.46
133.39
129.23
129.02
127.92
60.35
42.09
34.74
10.37
f1 (ppm)

(*S*)-(-)-*N*-(*tert*-butoxycarbonyl)-*N*,*O*-dimethyltyrosine

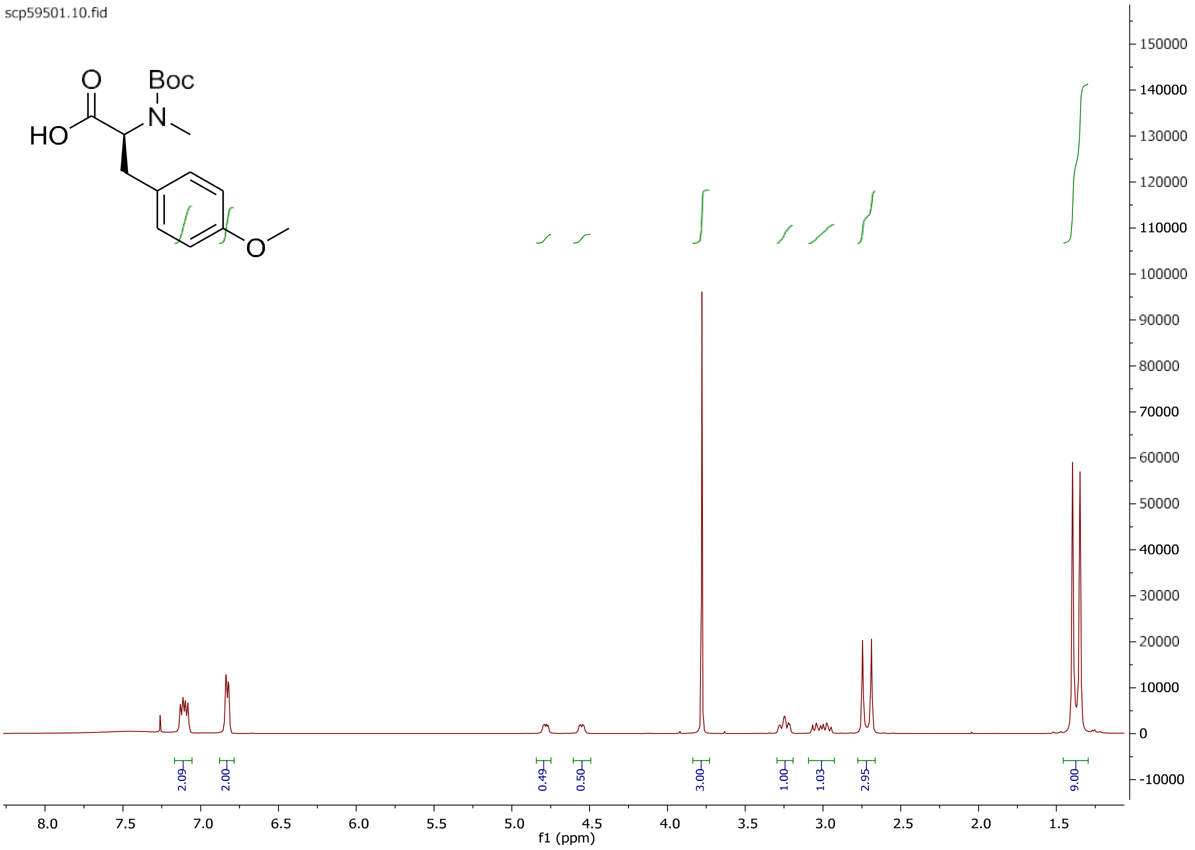

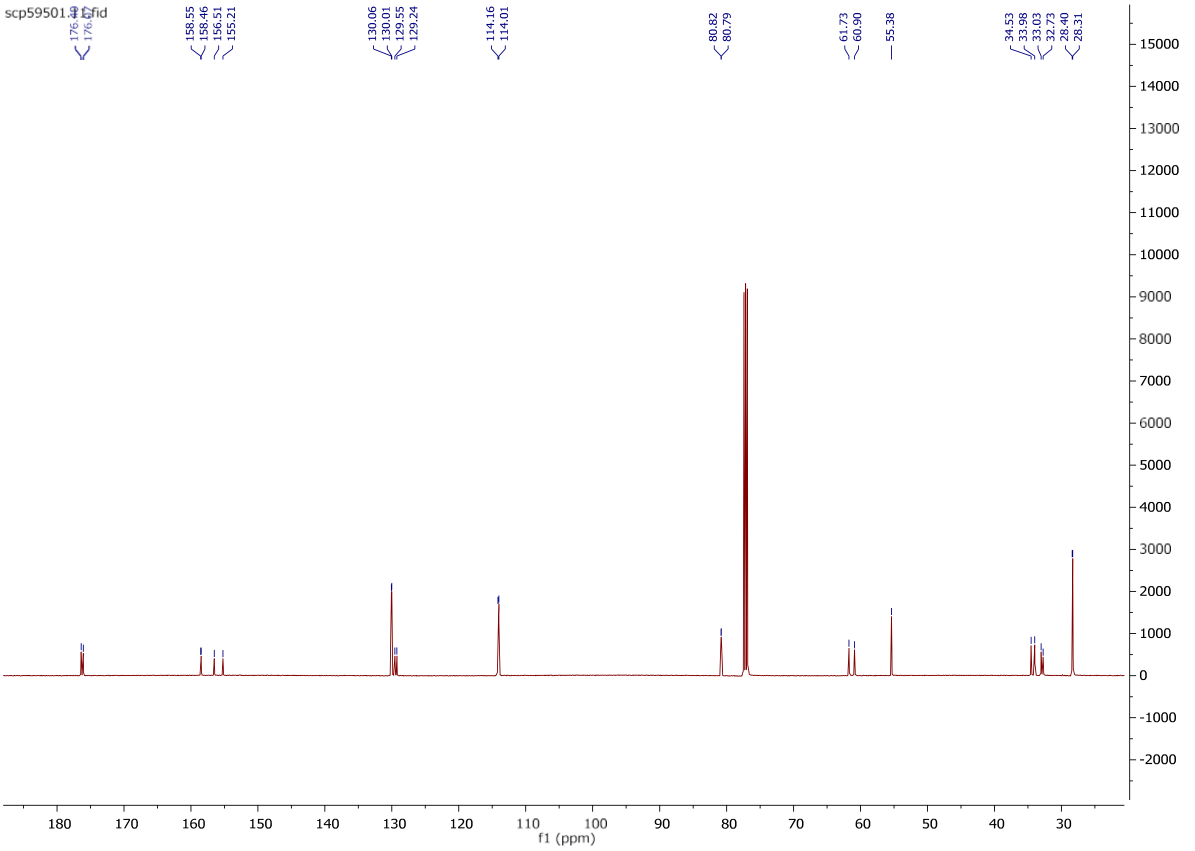

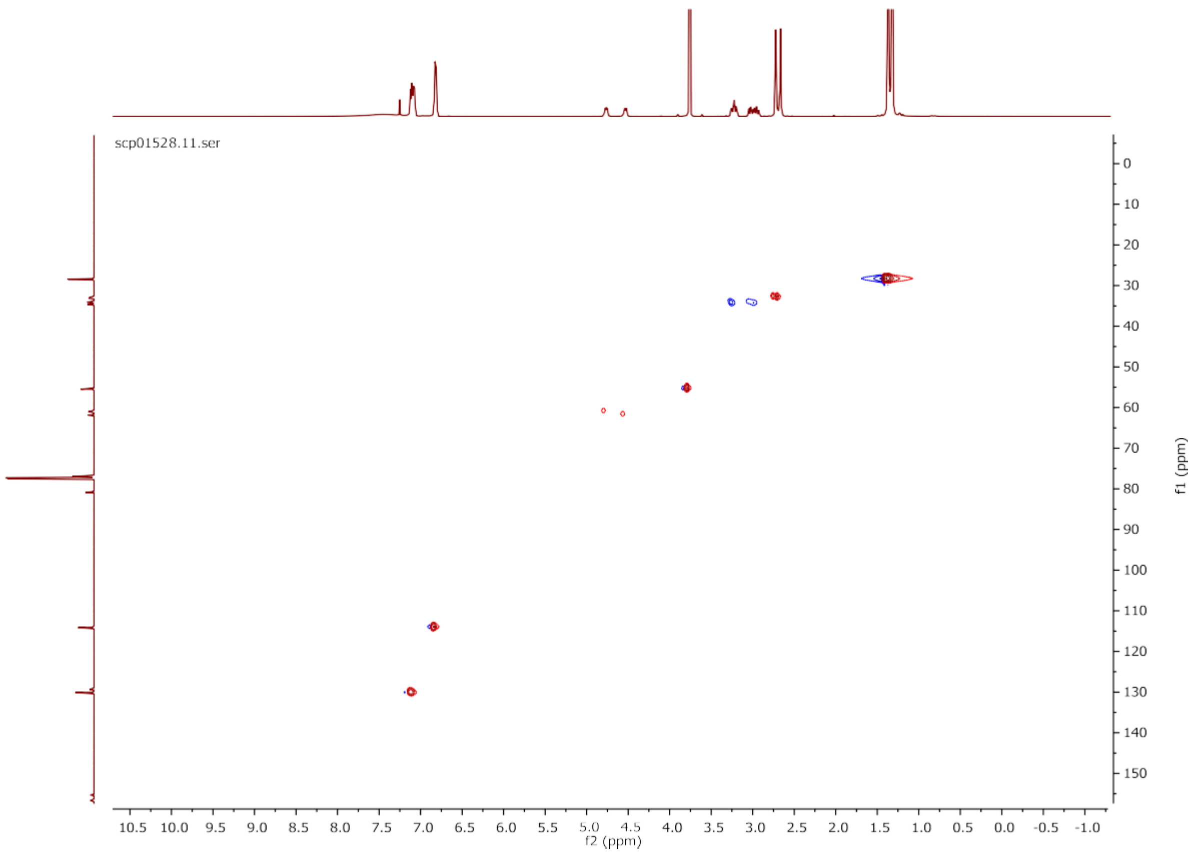

(*S*)-(+)- *N*,*O*-dimethyltyrosine

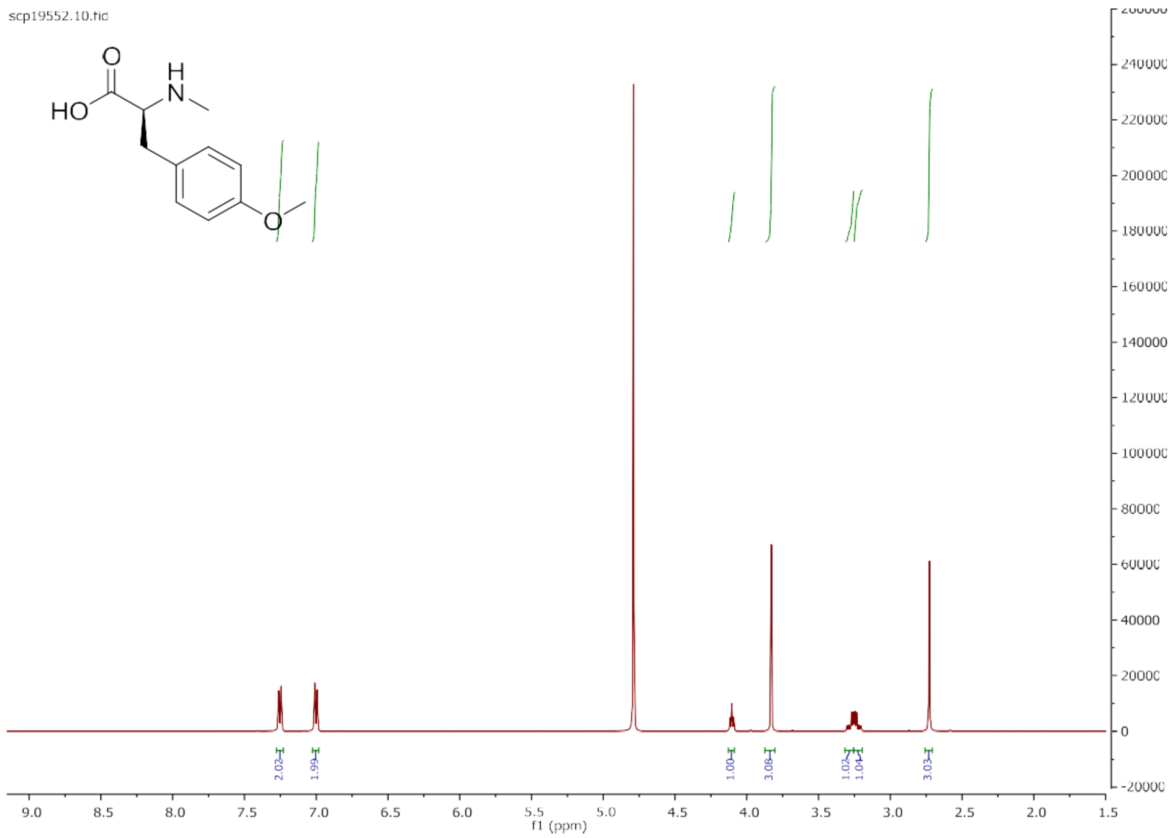

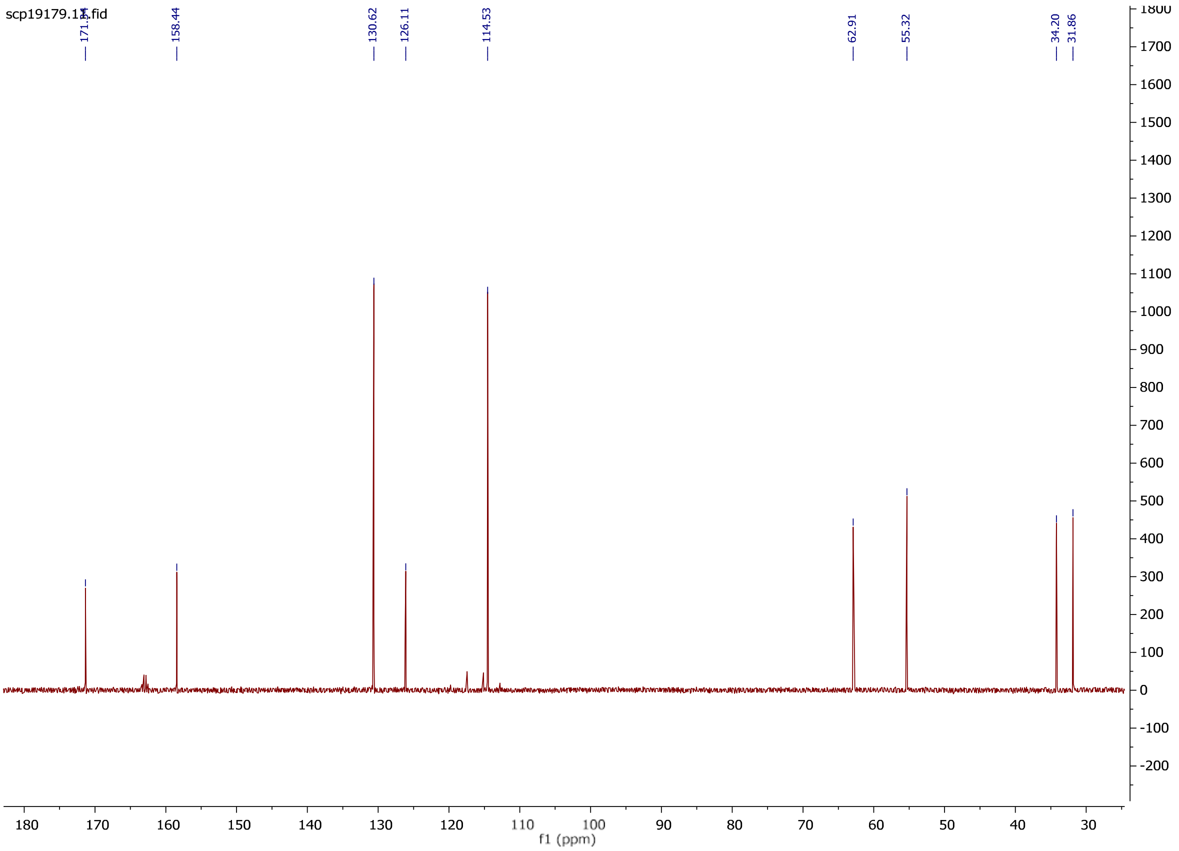
scp19179.11.fid
171.34
158.44
130.62
126.11
114.53
62.91
55.32
34.20
31.86
f1 (ppm)

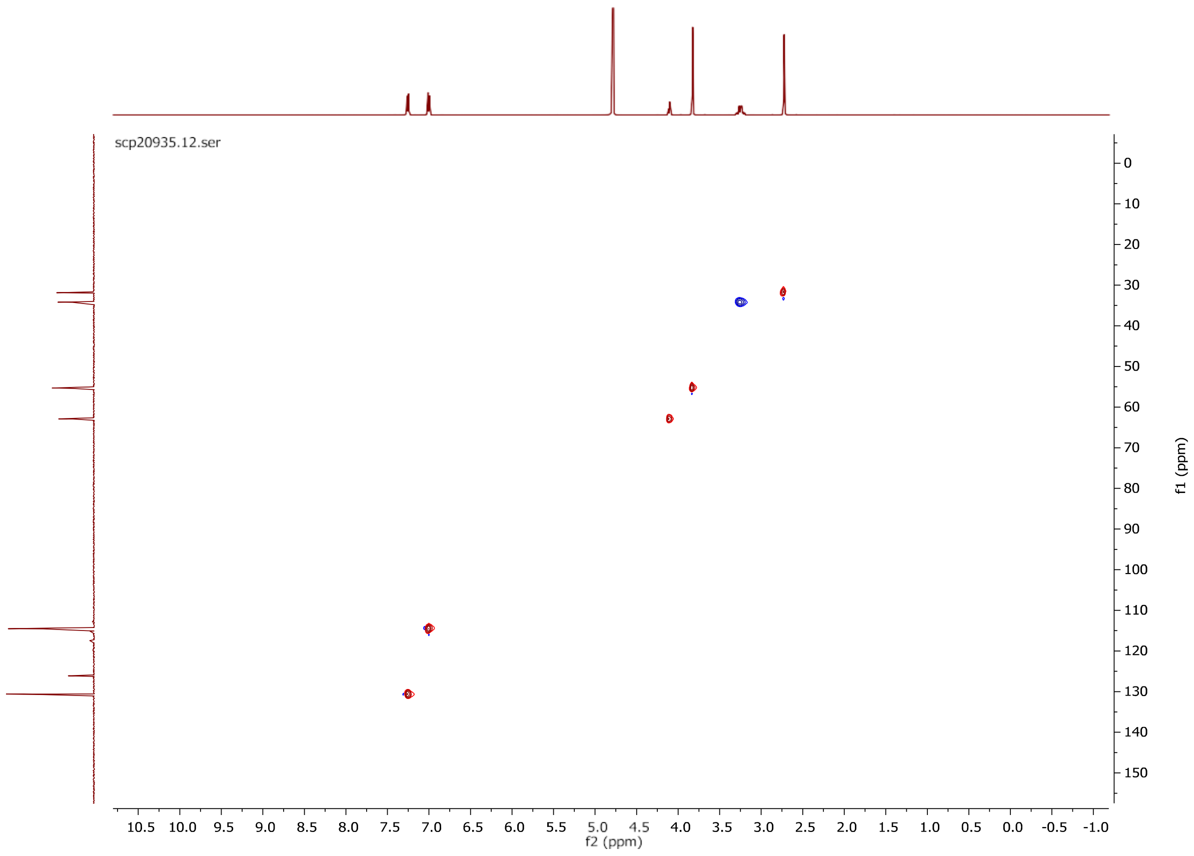
scp20935.12.ser
f2 (ppm)
f1 (ppm)

H. virescens injection assay

Table S12. Percentage mortality of *Heliothis virescens* on days 1, 2, 3 and 7 after injection with cycloaspeptide E **5**, 4F-cycloaspeptide E, spinosad as a positive control, or DMSO as a negative control. No mortality was observed for either of the cycloaspeptides.

		% mortality (days after injection)			
**Treatment**	**Rate**	**1**	**2**	**3**	**7**
Cycloaspeptide E	10 ug ul^{-1} (n = 6)	0	0	0	0
4F-Cycloaspeptide E	10 ug ul^{-1} (n = 6)	0	0	0	0
Spinosad (Positive control)	0.19 ug ul^{-1} (n = 6)	17	67	100	100
DMSO (Negative control)	Neat DMSO (n =6)	0	0	0	0

References

1. F. J. Jin, J. Maruyama, P. R. Juvvadi, M. Arioka and K. Kitamoto, *FEMS Microbiology Letters*, 2004, **239**, 79-85.
2. J. Smedsgaard, *Journal of Chromatography A*, 1997, **760**, 264-270.
3. R. D. Gietz, R. H. Schiestl, A. R. Willems and R. A. Woods, *Yeast*, 1995, **11**, 355-360.
4. K. Williams, A. J. Szwalbe, N. P. Mulholland, J. L. Vincent, A. M. Bailey, C. L. Willis, T. J. Simpson and R. J. Cox, *Angewandte Chemie International Edition*, 2016, **55**, 6784-6788.
5. M. H. Medema, K. Blin, P. Cimermancic, V. de Jager, P. Zakrzewski, M. A. Fischbach, T. Weber, E. Takano and R. Breitling, *Nucleic Acids Research*, 2011, **39**, W339-W346.
6. P. Wiemann, C.-J. Guo, J. M. Palmer, R. Sekonyela, C. C. Wang and N. P. Keller, *Proceedings of the National Academy of Sciences*, 2013, **110**, 17065-17070.
7. Y.-H. Chooi, R. Cacho and Y. Tang, *Chemistry & biology*, 2010, **17**, 483-494.
8. J. Marui, N. Yamane, S. Ohashi-Kunihiro, T. Ando, Y. Terabayashi, M. Sano, S. Ohashi, E. Ohshima, K. Tachibana and Y. Higa, *Journal of bioscience and bioengineering*, 2011, **112**, 40-43.
9. R. D. Finn, T. K. Attwood, P. C. Babbitt, A. Bateman, P. Bork, A. J. Bridge, H.-Y. Chang, Z. Dosztányi, S. El-Gebali, M. Fraser, J. Gough, D. Haft, G. L. Holliday, H. Huang, X. Huang, I. Letunic, R. Lopez, S. Lu, A. Marchler-Bauer, H. Mi, J. Mistry, D. A. Natale, M. Necci, G. Nuka, C. A. Orengo, Y. Park, S. Pesseat, D. Piovesan, S. C. Potter, N. D. Rawlings, N. Redaschi, L. Richardson, C. Rivoire, A. Sangrador-Vegas, C. Sigrist, I. Sillitoe, B. Smithers, S. Squizzato, G. Sutton, N. Thanki, P. D. Thomas, Silvio C. E. Tosatto, C. H. Wu, I. Xenarios, L.-S. Yeh, S.-Y. Young and A. L. Mitchell, *Nucleic Acids Research*, 2017, **45**, D190-D199.
10. A. Marchler-Bauer, Y. Bo, L. Han, J. He, C. J. Lanczycki, S. Lu, F. Chitsaz, M. K. Derbyshire, R. C. Geer, N. R. Gonzales, M. Gwadz, D. I. Hurwitz, F. Lu, G. H. Marchler, J. S. Song, N. Thanki, Z. Wang, R. A. Yamashita, D. Zhang, C. Zheng, L. Y. Geer and S. H. Bryant, *Nucleic Acids Research*, 2017, **45**, D200-D203.
11. T. Saruwatari, F. Yagishita, T. Mino, H. Noguchi, K. Hotta and K. Watanabe, *Chembiochem*, 2014, **15**, 656-659.
12. Y. M. Chiang, E. Szewczyk, T. Nayak, A. D. Davidson, J. F. Sanchez, H. C. Lo, W. Y. Ho, H. Simityan, E. Kuo, A. Praseuth, K. Watanabe, B. R. Oakley and C. C. Wang, *Chem. Biol.*, 2008, **15**, 527-532.
13. M. Umemura, N. Nagano, H. Koike, J. Kawano, T. Ishii, Y. Miyamura, M. Kikuchi, K. Tamano, J. Yu, K. Shin-ya and M. Machida, *Fungal genetics and biology : FG & B*, 2014, **68**, 23-30.
14. K. Koo and W. D. Stuart, *Genome*, 1991, **34**, 644-651.
15. K. Nishiwaki, N. Hayashi, S. Irie, D. H. Chung, S. Harashima and Y. Oshima, *Mol Gen Genet*, 1987, **208**, 159-167.
16. K. A. K. Pahirulzaman, K. Williams and C. M. Lazarus, *Methods in Enzymology*, 2012, **517**, 241-260.
17. G. Schmeda-Hirschmann, E. Hormazabal, J. A. Rodriguez and C. Theoduloz, *Zeitschrift Fur Naturforschung Section C-a Journal of Biosciences*, 2008, **63**, 383-388.
18. P. Lewer, P. R. Graupner, D. R. Hahn, L. L. Karr, D. O. Duebelbeis, J. M. Lira, P. B. Anzeveno, S. C. Fields, J. R. Gilbert and C. Pearce, *Journal of Natural Products*, 2006, **69**, 1506-1510.
19. S.-y. Aoki, T. Oi, K. Shimizu, R. Shiraki, K.-i. Takao and K.-i. Tadano, *Bull. Chem. Soc. Jpn.*, 2004, **77**, 1703-1716.
20. W. Oppolzer, P. Cintas- Moreno, O. Tamura and F. Cardinaux, *Helv. Chim. Acta*, 1993, **76**, 187-196.
21. Y. Ohfune, N. Kurokawa, N. Higuchi, M. Saito, M. Hashimoto and T. Tanaka, *Chem. Lett.*, 1984, **13**, 441-444.
22. D. L. Boger and D. Yohannes, *The Journal of Organic Chemistry*, 1988, **53**, 487-499.
23. S. Matthew, C. Ross, V. J. Paul and H. Luesch, *Tetrahedron*, 2008, **64**, 4081-4089.
